# Synthesis, Antimicrobial Activity and Cytotoxicity of Novel (Piperidin-4-yl)adamantane-1-carboxylate *N*-Substituted Derivatives

**DOI:** 10.3390/molecules31030439

**Published:** 2026-01-27

**Authors:** Kaldybay D. Praliyev, Gulmira S. Akhmetova, Ulzhalgas B. Issayeva, Samir A. Ross, Manas T. Omyrzakov, Ilya S. Korotetskiy, Ardak B. Jumagaziyeva, Aigul E. Malmakova, Tulegen M. Seilkhanov, Ubaidilla M. Datkhayev, Lyudmila N. Ivanova, Zhanar A. Iskakbayeva, Olzhas T. Seilkhanov, Natalya V. Zubenko

**Affiliations:** 1 Laboratory of Synthetic and Natural Medicinal Compounds Chemistry, A.B. Bekturov Institute of Chemical Sciences JSC, 106 Sh. Ualikhanov St., Almaty 050010, Kazakhstan; praliyevkd@mail.ru (K.D.P.); ulyano.iss@gmail.com (U.B.I.); malmakova@mail.ru (A.E.M.); 2 National Center for Natural Products Research, School of Pharmacy, The University of Mississippi, Oxford, MS 38677, USA; 3 School of Pharmacy, Asfendiyarov Kazakh National Medical University, Almaty 050000, Kazakhstan; manaman38@gmail.com (M.T.O.); u.datkhayev@gmail.com (U.M.D.); 4 Scientific Center for Anti-Infectious Drugs JSC, Almaty 050060, Kazakhstan; laeda81@gmail.com (I.S.K.); r_dawa@mail.ru (A.B.J.); lyudmila_69.69@mail.ru (L.N.I.); zhanara_07_74@mail.ru (Z.A.I.); zubenkonatalie@gmail.com (N.V.Z.); 5 Department Biochemical Engineering, LLC International Engineering and Technological University, Almaty 050060, Kazakhstan; 6 LLP Research and Production Association Kazpharmacom, Irgeli 040900, Kazakhstan; 7 The Laboratory of Engineering Profile of NMR Spectroscopy, Sh. Ualikhanov Kokshetau State University, Kokshetau 020000, Kazakhstan; tseilkhanov@mail.ru (T.M.S.); seilkhanov@mail.ru (O.T.S.)

**Keywords:** piperidines, adamantancarboxylates, cytotoxicity, antimicrobial activity, antifungal activity

## Abstract

The cyclic adamantane framework possesses unique properties such as bulkiness, symmetry, and high lipophilicity. Research aimed at discovering new pharmaceutical agents within the adamantane series continues. In the present work, a targeted modification was carried out to combine two pharmacophore fragments—adamantane and piperidine—within a single molecule. Based on a series of *N-*substituted piperidin-4-ones, the corresponding secondary alcohols were obtained by reduction with sodium borohydride in isopropanol and subsequent acylation of these alcohols with adamantane carbonyl chloride yielded the corresponding adamantane-carboxylate esters. The structure of the synthesized compounds was studied by NMR methods, including COSY (^1^H-^1^H), HMQC (^1^H-^13^C) and HMBC (^1^H-^13^C) techniques. The values of chemical shifts, multiplicities, and integrated intensities of ^1^H and ^13^C signals in one-dimensional NMR spectra were determined. The results of COSY (^1^H-^1^H), HMQC (^1^H-^13^C), and HMBC (^1^H-^13^C) revealed homo- and heteronuclear interactions, confirming the structure of the studied compounds. The cytotoxic activities of the synthesized compounds were studied. It was found that the synthesized substituted piperidines bearing an adamantane fragment exhibit in vitro antimicrobial and antifungal activity against museum microbial strains (*Escherichia coli* ATCC 8739, *Staphylococcus aureus* ATCC 6538-P, *Candida albicans* ATCC 10231, *Cryptococcus neoformans*) and demonstrate significant advantages over the reference drugs used in clinical practice, such as fluconazole and ampicillin. These compounds are therefore recommended for further in-depth studies.

## 1. Introduction

Adamantane is an organic compound belonging to the class of polycyclic hydrocarbons. An analysis of recent scientific publications indicates that the search for new adamantane derivatives is one of the most rapidly developing areas in chemical and pharmaceutical research. This growing interest is driven by the wide spectrum of documented biological activities exhibited by compounds of this series [1]. Moreover, it is well known that the incorporation of an adamantane moiety into a molecular structure often leads to an increase in lipophilicity, which in turn may positively influence the bioavailability and enhance the therapeutic potential of newly synthesized substances [1].

Adamantane derivatives exhibit a wide range of biological activities, including antiviral [1,2,3,4], antidiabetic [5,6,7,8,9], antibacterial [10,11,12,13,14], antiplasmodial (antimalarial) [15,16], anticancer [17,18,19,20,21], and anti-inflammatory [22,23] effects.

**Figure 1:**
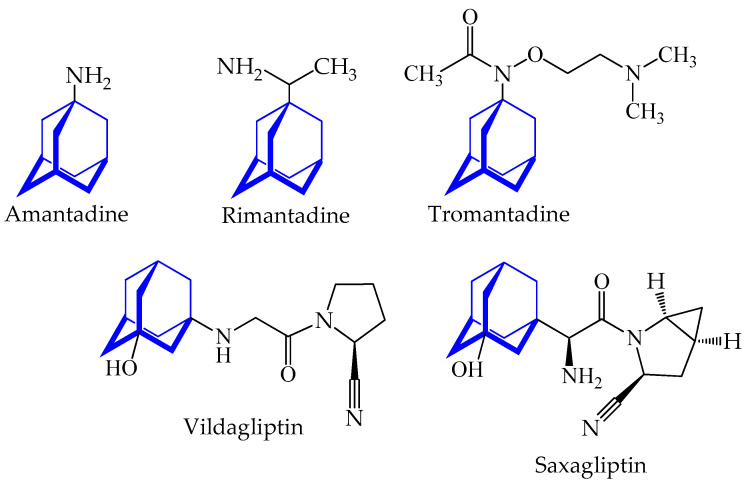
Pharmaceutical drugs containing an adamantane fragment.

The adamantane scaffold is also a structural component of many currently used pharmaceuticals. For instance, amantadine, rimantadine, and tromantadine are amine derivatives of adamantane that demonstrate pronounced antiviral activity. Several other compounds, such as vildagliptin and saxagliptin, are employed as antidiabetic agents in the treatment of type 2 diabetes mellitus, whereas adapalene is well known for its anti-inflammatory properties and is widely used in acne therapy (Figure 1) [9,22].

In recent years, increasing attention has also been directed toward the antimicrobial potential of adamantane-containing compounds, which is associated with the growing resistance of pathogenic microorganisms to commonly used antibacterial agents. This trend greatly complicates the treatment of infections and highlights the urgent need for new biologically active molecules [24,25,26,27].

Popiołek et al. [14] synthesized a series of new isothiourea derivatives containing an adamantane moiety. These compounds demonstrated pronounced antibacterial activity against both Gram-positive and Gram-negative microorganisms. Moreover, the obtained derivatives significantly reduced blood serum glucose levels, surpassing gliclazide in efficacy [14].

In the study by Pham and colleagues [13], new hydrazide–hydrazones bearing a 1-adamantanecarbonyl fragment were synthesized and evaluated against several Gram-positive and Gram-negative bacteria, as well as the fungus *Candida albicans*. The biological screening revealed that four of the compounds exhibited marked antibacterial activity against Gram-positive strains and *Candida albicans*, demonstrating activity comparable to or surpassing that of known reference antimicrobial agents [13]. According to more recent reports, adamantane derivatives are also capable of affecting biofilm formation in *Enterococcus faecalis*, *Pseudomonas aeruginosa*, and methicillin-resistant *Staphylococcus aureus*, which is likely associated with their membranotropic mechanism of action [28,29,30].

For many years, our research group, led by Professor K. D. Praliyev, has been engaged in the search for drug candidates among C- and *N*-substituted piperidine derivatives exhibiting diverse biological activities [31,32,33,34,35,36,37,38,39], including pronounced anti-infective activity [40,41,42].

Research in this field dates back to the 1970s. As a result of these studies, the analgesic prosidol was introduced into medical practice [36,37,38]. Prosidol exceeds morphine and promedol in analgesic activity by 3–30 times and surpasses them in the breadth of pharmacological effects by 2–100 times.

Of particular interest for practical medicine is the drug kazkain [39], which has successfully completed Phase I clinical trials and demonstrated high efficacy for two recommended indications, namely as an anesthetic and an antiarrhythmic agent.

Considering the broad spectrum of biological activities reported for adamantane derivatives in the literature, the aim of the present study was to synthesize new piperidine derivatives incorporating an adamantane fragment and to evaluate their in vitro antimicrobial activity and cytotoxicity.

The objective of this research was to determine how the combination of two pharmacophores—piperidine and adamantine—within a single molecular scaffold influences antibacterial and antifungal activity.

## 2. Results and Discussion

### 2.1. Synthesis of Piperidine-Based Adamantane Carboxylates

**Scheme 1:**
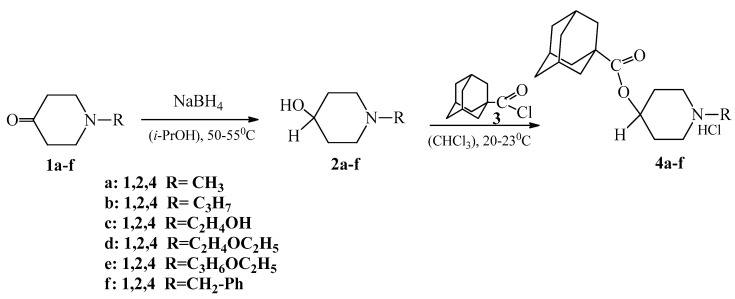
Synthesis of Piperidine-Based Adamantane Carboxylates **4a**–**f**.

For the purpose of searching for new pharmacologically active compounds among adamantane-containing piperidine derivatives, the reduction of [1-alkyl-, 1-hydroxyalkyl-, 1-alkoxyalkyl-, 1-arylalkyl-]-piperidin-4-ones (**1a**–**f**) with sodium borohydride in absolute isopropanol afforded the corresponding secondary alcohols (**2a**–**f**) in good yields (91–95%) [43,44,45,46,47,48]. Subsequent acylation of these alcohols with adamantane-1-carbonyl chloride led to the synthesis of the corresponding hydrochlorides of adamantane carboxylate esters (**4a**–**f**) in satisfactory yields (45.2–81.1%) (Scheme 1). The acylation reaction was carried out in chloroform using a piperidinol–acylating agent molar ratio of 1:3.

The obtained adamantane-1-carboxylate esters (**4a**–**f**) are well-crystallizing compounds with sharp melting points, and their composition and structure were confirmed by elemental analysis, IR and NMR spectroscopy, while their individuality was verified by thin-layer chromatography (Al_2_O_3_, benzene:dioxane 3:2, Rf = 0.81–0.91). (Table S1 in Supplementary Materials).

In the IR spectra of the piperidinols, absorption bands corresponding to the stretching vibrations of the hydroxyl group appear in the range of 3348–3600 cm^−1^. Intense absorption bands at 1721–1727 cm^−1^, attributed to the C=O stretching vibrations of the ester group, confirm the formation of the target piperidinol-4 adamantane carboxylate esters (**4a**–**f**).

In the ^13^C NMR spectra of the adamantane-carbonyl-oxy derivatives (**4a**–**f**), intense singlet signals corresponding to the carbon atoms of the ester carbonyl group are observed in the range of 171.55–172.64 ppm. (Table S1, Figures S1–S6).

The structures of the synthesized compounds (**4a**–**f**) were studied by ^1^H and ^13^C NMR methods, including the COSY (^1^H-^1^H), HMQC (^1^H-^13^C), and HMBC (^1^H-^13^C) techniques. The values of the chemical shifts, multiplicity, and integral intensity of ^1^H and ^13^C signals in one-dimensional NMR spectra were determined. The results of COSY (^1^H-^1^H), HMQC (^1^H-^13^C), and HMBC (^1^H-^13^C) revealed homo- and heteronuclear interactions, confirming the structure of the studied compounds (Table S1 in Supplementary Materials).

The detailed ^1^H and ^13^C NMR spectra of the compounds (**4a**–**f**) are provided in the Supplementary Materials (Figures S7–S35).

In the ^1^H-^1^H COSY spectra of compound **4a**, spin–spin correlations through three bonds of protons of adjacent methylene–methylene and methylene–methine groups are observed H^14ax,16ax,18ax^-H^13,15,17^ (1.62, 1.91 and 1.91, 1.62), H^3ax,5ax^-H^3eq,5eq^ (1.79, 2.11 and 2.11, 1.79), H^3eq,5eq^-H^2ax,6ax^ (2.08, 2.94 and 2.94, 2.08), H^3eq,5eq^-H^2eq,6eq^ (2.08, 3.23 and 3.23, 2.08), H^3eq,5eq^-H^2ax,6ax^ (1.83, 3.05 and 3.05, 1.83), H^3eq,5eq^-H^4^ (1.82, 4.74 and 4.74, 1.82), H^2ax,6ax^-H^2eq,6eq^ (2.90, 3.23 and 3.23, 2.90; 3.07, 3.33 and 3.33, 3.07) ppm.

Heteronuclear interactions of protons with carbon atoms through one bond were determined using ^1^H-^13^C HMQC spectroscopy for the following pairs present in the compound: H^14,16,18^-C^14,16,18^ (1.62, 36.34), H^12,19,20^-C^12,19,20^ (1.80, 35.62), H^13,15,17^-C^13,15,17^ (1.92, 27.94), H^3ax,5ax^-C^3,5^ (1.79, 27.73), H^3eq,5eq^-C^3,5^ (2.10, 27.47), H^2ax,6ax^-C^2,6^ (3.08, 48.85), H^2eq,6eq^-C^2,6^ (3.24, 49.28), H^7^-C^7^ (2.65, 42.62), H^4^-C^4^ (4.88, 63.69) ppm.

Heteronuclear interactions of protons with carbon atoms through two or more bonds were determined using ^1^H-^13^C HMBC spectroscopy for the following pairs present in the compound: H^14ax,16ax,18ax^-C^13,15,17^ (1.62, 27.93); H^12ax,19ax,20ax^-C^13,15,17^ (1.73, 27.64), H^12ax,19ax,20ax^-C^14,16,18^ (1.73, 36.48), H^12ax,19ax,20ax^-C^12,19,20^ (1.73, 38.60), H^12ax,19ax,20ax^-C^9^ (1.73, 176.23); H^12eq,19eq,20eq^-C^13,15,17^ (1.88, 27.77), H^12eq,19eq,20eq^-C^14,16,18^ (1.88, 36.48), H^12eq,19eq,20eq^-C^9^ (1.88, 176.23); H^7^-C^2,6^ (2.62, 51.98; 2.62, 48.56; 2.70, 49.27); H^2eq,6eq^-C^4^ (3.34, 67.37; 3.23, 63.81); H^4^-C^2,6^ (4.88, 49.29) ppm.

In the ^1^H-^1^H COSY spectra of compound **4b**, spin–spin correlations through three bonds of protons of adjacent methyl–methylene, methylene–methylene, and methylene–methine groups are observed, including H^9^-H^8^ (0.83, 1.67 and 1.67, 0.83), H^8^-H^7^ (1.68, 2.91 and 2.91, 1.68), H^16ax,18ax,20ax^-H^15,17,19^ (1.61, 1.92 and 1.92, 1.61), H^3ax,5ax^-H^3eq,5eq^ (1.78, 2.16 and 2.16, 1.78), H^2ax,6ax^-H^2eq,6eq^ (2.92, 3.32 and 3.32, 2.92), H^3eq,5eq^-H^4^ (1.92, 4.75 and 4.75, 1.92) ppm.

Heteronuclear interactions of protons with carbon atoms through one bond were determined using ^1^H-^13^C HMQC spectroscopy for the following pairs present in the compound: H^9^-C^9^ (0.82, 11.64), H^8^-C^8^ (1.68, 17.34), H^7^-C^7^ (2.94, 57.52), H^4^-C^4^ (4.88, 64.30), H^15,17,19^-C^15,17,19^ (1.91, 27.95), H^16,18,20^-C^16,18,20^ (1.61, 36.46), H^14ax,21ax,22ax^-C^14,21,22^ (1.72, 38.70), H^14eq,21eq,22eq^-C^14,21,22^ (1.78, 38.70), H^3ax,5ax^-C^3,5^ (1.74, 27.79), H^3eq,5eq^-C^3,5^ (2.12, 27.79), H^2ax,6ax^-C^2,6^ (32.99, 49.66), H^2eq,6eq^-C^2,6^ (3.39, 49.82) ppm.

In the ^1^H-^1^H COSY spectra of compound **4c**, spin–spin correlations through three bonds of protons of adjacent methylene–methylene and methylene–methine groups are observed, including H^16ax,18ax,20ax^-H^14ax,21ax,22ax^ (1.61, 1.79 and 1.79, 1.61), H^16ax,18ax,20ax^-H^15,17,19^ (1.61, 1.92 and 1.92, 1.61), H^14ax,21ax,22ax^-H^15,17,19^ (1.79, 1.89 and 1.89, 1.79), H^7^-H^8^ (3.31, 4.35 and 4.35, 3.31) ppm.

Heteronuclear interactions of protons with carbon atoms through one bond were determined using ^1^H-^13^C HMQC spectroscopy for the following pairs present in the compound: H^16,18,20^-C^16,18,20^ (1.62, 36.42), H^14,21,22^-C^14,21,22^ (1.78, 38.72), H^15,17,19^-C^15,17,19^ (1.92, 27.78), H^3ax,5ax^-C^3,5^ (1.68, 31.88), H^3eq,5eq^-C^3,5^ (1.91, 31.95), H^2ax,6ax^-C^2,6^ (2.93, 51.08), H^2eq,6eq^-C^2,6^ (3.17, 47.75), H^7^-C^7^ (3.29, 54.29), H^8^-C^8^ (4.33, 59.47), H^4^-C^4^ (4.89, 64.13) ppm.

Heteronuclear interactions of protons with carbon atoms through two or more bonds were determined using ^1^H-^13^C HMBC spectroscopy for the following pairs present in the compound: H^16ax,18ax,20ax^-C^15,17,19^ (1.60, 27.73); H^14ax,21ax,22ax^-C^16,18,20^ (1.78, 36.60); H^15,17,19^-C^14,21,22^ (1.93, 38.51) ppm.

In the ^1^H-^1^H COSY spectra of compound **4d**, spin–spin correlations through three bonds of protons of adjacent methylene–methylene and methylene–methine groups are observed, including H^18ax,20ax,22ax^-H^17,19,21^ (1.62, 1.92 and 1.92, 1.62), H^3ax,5ax^-H^3eq,5eq^ (1.79, 2.16 and 2.16, 1.79), H^11^-H^10^ (1.08, 3.43 and 3.43, 1.08), H^7^-H^8^ (3.25, 3.74 and 3.74, 3.25) ppm.

Heteronuclear interactions of protons with carbon atoms through one bond were determined using ^1^H-^13^C HMQC spectroscopy for the following pairs present in the compound: H^11^-C^11^ (1.08, 15.48), H^18,20,22^-C^18,20,22^ (1.63, 36.41), H^16ax,23ax,24ax^-C^16,23,24^ (1.74, 38.74), H^16eq,23eq,24eq^-C^16,23,24^ (1.81, 38.74), H^17,19,21^-C^17,19,21^ (1.92, 27.85), H^3ax,5ax^-C^3,5^ (1.81, 27.56), H^3eq,5eq^-C^3,5^ (2.14, 27.56), H^2ax,6ax^-C^2,6^ (3.07, 50.49; 3.04, 47.92), H^2eq,6eq^-C^2,6^ (3.33, 48.11; 3.42, 50.58), H^7^-C^7^ (3.21, 55.11), H^8^-C^8^ (3.74, 64.73), H^4^-C^4^ (4.89, 64.07), H^10^-C^10^ (3.42, 66.16) ppm.

Heteronuclear interactions of protons with carbon atoms through two or more bonds were determined using ^1^H-^13^C HMBC spectroscopy for the following pairs present in the compound: H^11^-C^10^ (1.08, 66.29); H^16ax,23ax,24ax^-C^17,19,21^ (1.80, 28.82); H^10^-C^11^ (3.42, 15.63), H^10^-C^8^ (3.42, 64.87); H^8^-C^10^ (3.74, 66.29); H^4^-C^2,6^ (4.88, 48.17) ppm.

In the ^1^H-^1^H COSY spectra of compound **4e**, spin–spin correlations through three bonds of protons of adjacent methylene–methylene and methylene–methine groups are observed, including H^19ax,21ax,23ax^-H^18,20,22^ (1.62, 1.93 and 1.93, 1.62), H^17ax,24ax,25ax^-H^18,20,22^ (1.78, 1.93 and 1.93, 1.78), H^3ax,5ax^-H^3eq,5eq^ (1.80, 2.15 and 2.15, 1.80), H^2ax,6ax^-H^2eq,6eq^ (2.92, 3.34 and 3.34, 2.92; 2.99, 3.41 and 3.41, 2.99), H^8^-H^7^ (1.94, 3.07 and 3.07, 1.94), H^12^-H^11^ (1.06, 3.39 and 3.39, 1.06), H^9^-H^11^ (1.93, 3.37 and 3.37, 1.93) ppm.

Heteronuclear interactions of protons with carbon atoms through one bond were determined using ^1^H-^13^C HMQC spectroscopy for the following pairs present in the compound: H^12^-C^12^ (1.06, 15.66), H^9,11^-C^9,11^ (3.37, 67.31), H^8^-C^8^ (1.93, 24.26), H^7^-C^7^ (3.05, 54.25), H^18,20,22^-C^18,20,22^ (1.92, 27.88), H^17ax,24ax,25ax^-C^17,24,25^ (1.74, 38.78), H^17eq,24eq,25eq^-C^17,24,25^ (1.81, 38.69), H^3ax,5ax^-C^3,5^ (1.62, 27.61), H^3eq,5eq^-C^3,5^ (2.08, 27.79), H^2ax,6ax^-C^2,6^ (2.91, 47.59; 2.99, 49.88), H^2eq,6eq^-C^2,6^ (3.33, 47.54; 3.41, 49.88), H^4^-C^4^ (4.90, 64.17) ppm.

Heteronuclear interactions of protons with carbon atoms through two or more bonds were determined using ^1^H-^13^C HMBC spectroscopy for the following pairs present in the compound: H^12^-C^11^ (1.06, 66.25); H^17ax,24ax,25ax^-C^19,21,23^ (1.75, 36.44), H^17ax,24ax,25ax^-C^18,20,22^ (1.75, 27.70); H^19ax,21ax,23ax^-C^18,20,22^ (1.63, 27.95); H^8^-C^7^ (1.91, 54.18), H^8^-C^9^ (1.91, 67.18); H^11^-C^12^ (3.37, 15.73), H^11^-C^8^ (3.37, 24.44), H^11^-C^7^ (3.37, 54.40) ppm.

In the ^1^H-^1^H COSY spectra of compound **4f**, spin–spin correlations through three bonds of protons of adjacent methylene–methylene, methylene–methine, and methine–methine groups are observed, including H^20ax,22ax,24ax^-H^19,21,23^ (1.58, 1.90 and 1.90, 1.58), H^20eq,22eq,24eq^-H^19,21,23^ (1.72, 1.90 and 1.90, 1.72), H^3ax,5ax^-H^3eq,5eq^ (1.81, 2.13 and 2.13, 1.81), H^3eq,5eq^-H^2ax,6ax^ (2.11, 2.91 and 2.91, 2.11), H^2ax,6ax^-H^2eq,6eq^ (2.93, 3.21 and 3.21, 2.93), H^9,13^-H^10,12^ (7.40, 7.61 and 7,61, 7.40) ppm.

Heteronuclear interactions of protons with carbon atoms through one bond were determined using ^1^H-^13^C HMQC spectroscopy for the following pairs present in the compound: H^20,22,24^-C^20,22,24^ (1.61, 36.50), H^18,25,26^-C^18,25,26^ (1.71, 38.72), H^19,21,23^-C^19,21,23^ (1.91, 27.84), H^3ax,5ax^-C^3,5^ (1.71, 26.80), H^3eq,5eq^-C^3,5^ (2.10, 26.80), H^2ax,6ax^-C^2,6^ (2.89, 46.76), H^2eq,6eq^-C^2,6^ (3.26, 46.81), H^7^-C^7^ (4.29, 59.04; 4.21, 58.70), H^4^-C^4^ (4.87, 64.05), H^10,12^-C^10,12^ (7.60, 132.08), H^11^-C^11^ (7.40, 130.42), H^9,13^-C^9,13^ (7.40, 129.06) ppm.

Heteronuclear interactions of protons with carbon atoms through two or more bonds were determined using ^1^H-^13^C HMBC spectroscopy for the following pairs present in the compound: H^20ax,22ax,24ax^-C^19,21,23^ (1.57, 27.71), H^20ax,22ax,24ax^-C^18,25,26^ (1.57, 38.61); H^18,25,26^-C^19,21,23^ (1.72, 27.82), H^18,25,26^-C^20,22,24^ (1.72, 36.60); H^7^-C^2,6^ (4.29, 47.80), H^7^-C^9,13^ (4.29, 132.06); H^9,13^-C^10,12^ (7.41, 132.66); H^10,12^-C^7^ (7.59, 58.89), H^10,12^-C^9,13^ (7.59, 128.96) ppm.

### 2.2. Study of Antimicrobial Activity in In Vitro Experiments

To evaluate the antimicrobial efficacy of a series of new adamantanecarbonyloxy derivatives (**4a**–**f**), studies were carried out to assess their antibacterial and antifungal activity. Reference strains were used as research objects. The selection of test organisms included both Gram-positive and Gram-negative microorganisms: *Staphylococcus aureus* ATCC 6538-P, *Escherichia coli* ATCC 8739, as well as yeast-like fungi: *Candida albicans* ATCC 10231.

**Figure 2:**
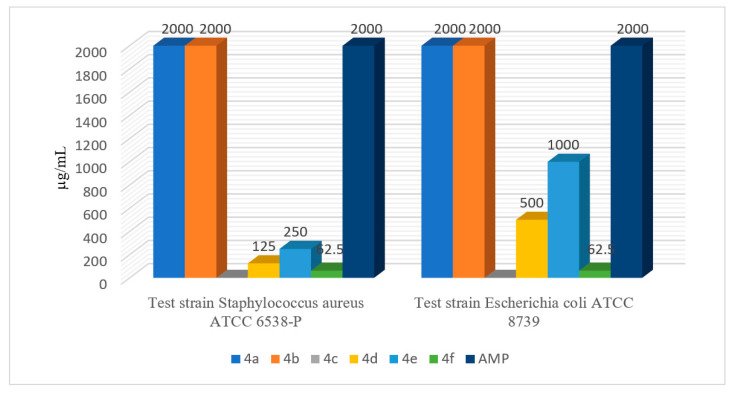
Evaluation of the Minimum Bactericidal Concentration of Compounds (**4a**–**f**) Against *Staphylococcus aureus* ATCC 6538-P and *Escherichia coli* ATCC 8739 (Test Conc, μg/mL).

**Figure 3:**
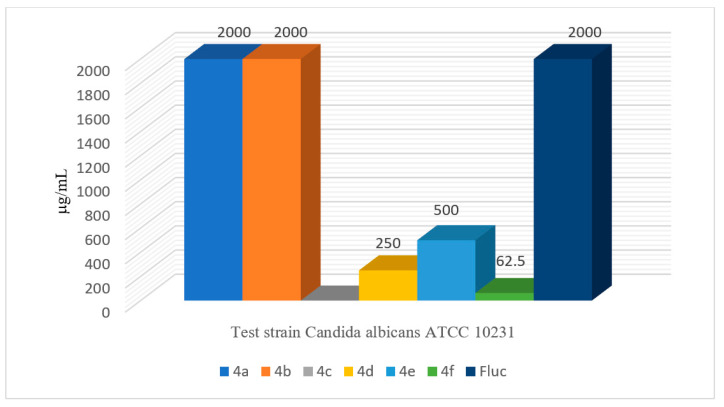
Evaluation of the Minimum Fungicidal Concentration Against *Candida albicans* ATCC 10231. (Test Conc, μg/mL).

The results of the antibacterial and fungicidal activity of the tested compounds (**4a**–**f**) against the susceptible microorganisms *Staphylococcus aureus* ATCC 6538-P, *Escherichia coli* ATCC 8739 and *Candida albicans* ATCC 10231 are presented in Table 1, Table 2 and Table 3, Figure 2 and Figure 3.

It was shown that the lowest activity against the *Staphylococcus aureus* strain was exhibited by samples **4a** and **4b**, whose minimum bactericidal concentration (MBC) was 2000 μg/mL, comparable to that of the reference drug. The highest antimicrobial activity against the tested culture was demonstrated by samples **4d**, **4e** and **4f**, with minimum bactericidal concentrations of 125 μg/mL, 250 μg/mL, and 62.5 μg/mL, respectively. These compounds also exhibited a bacteriostatic effect, i.e., the ability to inhibit bacterial growth. This effect was observed at concentrations of 62.5 μg/mL and 125 μg/mL and 31.25 μg/mL for compounds **4d**, **4e** and **4f** respectively. Compound **4c** did not demonstrate statistically significant antimicrobial activity against *Staphylococcus aureus* ATCC 6538-P (Table 1, Figure 2).

Thus, based on the results of the antimicrobial activity study, it was shown that among the tested compounds, compound **4f** exhibited the highest antimicrobial activity against *Staphylococcus aureus* ATCC 6538-P, surpassing ampicillin in efficacy by a factor of 32. Compound **4d** also exceeded the antimicrobial activity of ampicillin by a factor of 16, and compound **4e** by a factor of 8.

As seen from the data in Table 2, Figure 2, the analysis of the activity of compounds **4a**, **4b**, and the reference drug ampicillin shows that they exhibit bactericidal activity against the *Escherichia coli* ATCC 8739 strain at a concentration of 2000 μg/mL. The heteroorganic compound **4c** did not demonstrate bactericidal or bacteriostatic properties.

It is worth highlighting the adamantane carboxylates **4d**, **4e** and **4f**, whose minimum bactericidal concentrations causing the death of the *Escherichia coli* ATCC 8739 test strain were 500 μg/mL, 1000 μg/mL, and 62.5 μg/mL, respectively. Their bacteriostatic effects were observed at concentrations of 250 μg/mL, 500 μg/mL, and 31.25 μg/mL, respectively.

Thus, the highest efficacy against the *Escherichia coli* ATCC 8739 strain was demonstrated by compound **4f**, surpassing the reference drug ampicillin by a factor of 32.

The data on the fungicidal activity of the samples, compared with the antifungal drug fluconazole, are presented in Table 3, Figure 3. It was shown that the highest activity against yeast-like fungi of the genus *Candida* was exhibited by compounds **4f** and **4d**, for which the mycocidal concentrations were 62.5 μg/mL and 250 μg/mL, respectively. Samples **4a**, **4b**, and **4e** demonstrated comparable fungicidal activity at a concentration of 500 μg/mL. Compound **4c**, as well as the reference drug, did not exhibit fungicidal activity against *Candida albicans* ATCC 10231.

Thus, all compounds except **4c** showed antifungal activity against *Candida albicans* ATCC 10231. The highest efficacy against yeast-like fungi was demonstrated by compound **4f**, surpassing the reference drug fluconazole by a factor of 40.

Compound **4d** should also be highlighted, as it exceeded the activity of fluconazole by a factor of 10.

Thus, based on the microbiological studies of adamantane carboxylates of the piperidine series conducted in vitro, the following compounds can be identified as promising candidates for further testing on resistant museum and clinical strains: **4d**, **4e** and **4f** as agents active against *Staphylococcus aureus*; **4d**, **4e** and **4f** as agents active against *Escherichia coli*; **4a**, **4b**, **4d**, **4e**, and **4f** as agents active against *Candida albicans*.

Compound **4f** exhibited the highest activity against all museum microorganisms included in the experiment, at a concentration of 62.5 μg/mL.

Compound **4f** was additionally evaluated for in vitro antimicrobial activity against a broader spectrum of museum microbial strains. The data were compared with the activity of fluconazole, amphotericin B, ciprofloxacin, vancomycin, methicillin, cefotaxime, and meropenem. The results of the study are presented in Table 4 and Table 5. Compound **4f** demonstrated pronounced activity against the yeast-like fungus *Cryptococcus neoformans*, showing 100% growth inhibition in the primary test (Table 4). In the secondary test (Table 5), its half-maximal inhibitory concentration (IC_50_) was determined to be less than 0.8 μg/mL.

### 2.3. The Determination of Cytotoxicity of the Investigational Drugs In Vitro

**Figure 4:**
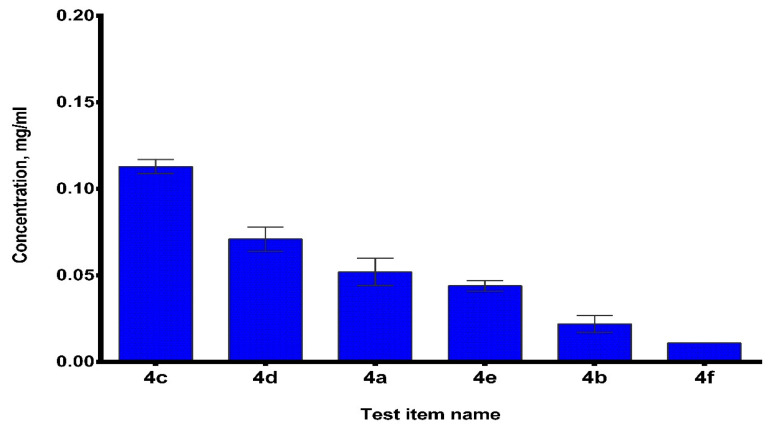
Toxicity series for substances against the MDCK cell culture.

**Figure 5:**
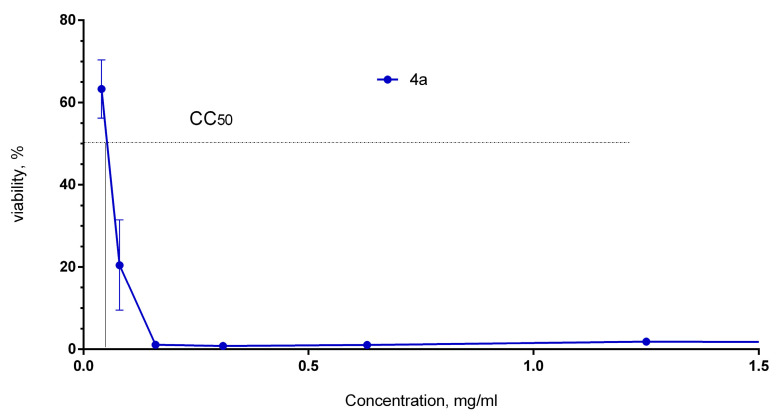
Cytotoxic effect of the Test item **4a** on MDCK cell culture.

**Figure 6:**
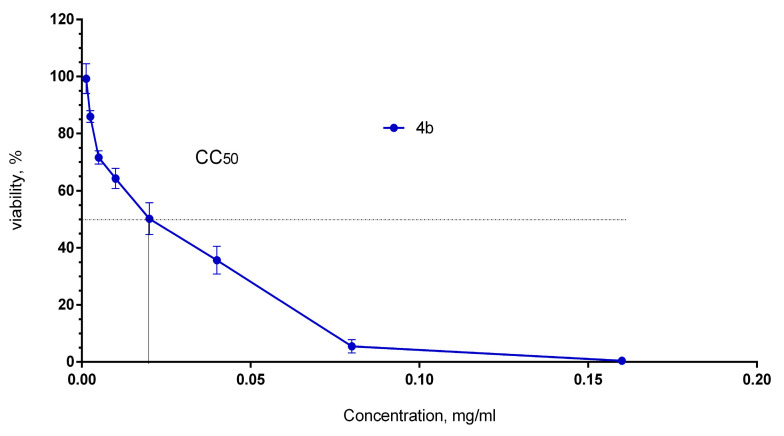
Cytotoxic effect of the substance **4b** on MDCK cell culture.

**Figure 7:**
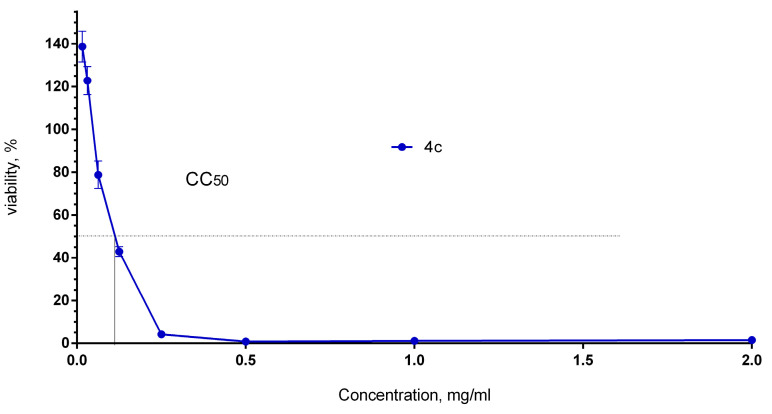
Cytotoxic effect of the substance **4c** on MDCK cell culture.

**Figure 8:**
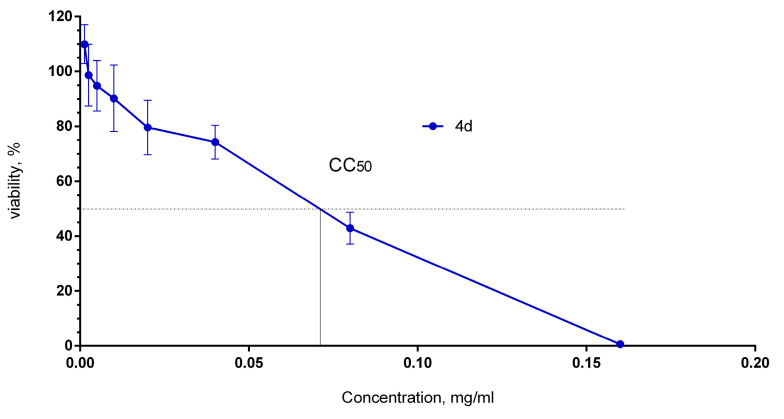
Cytotoxic effect of the substance **4d** on MDCK cell culture.

**Figure 9:**
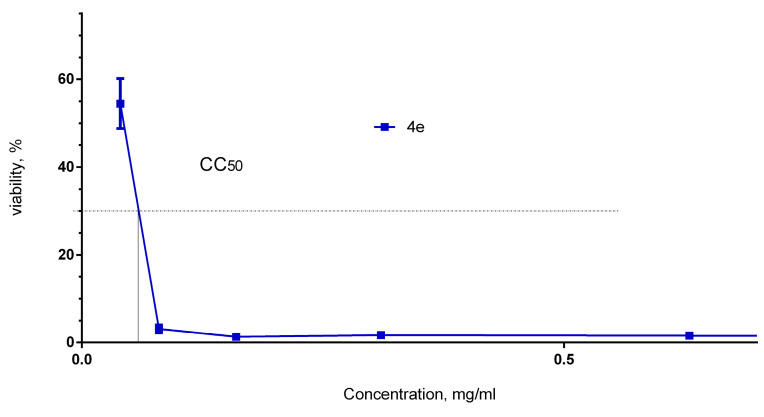
Cytotoxic effect of the Test item **4e** on MDCK cell culture.

**Figure 10:**
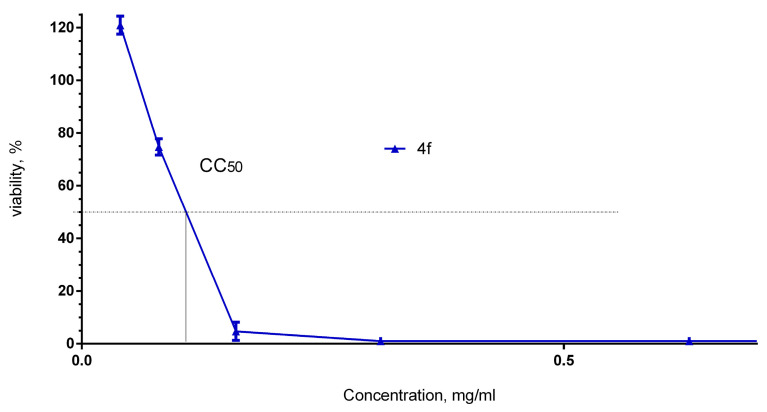
Cytotoxic effect of the substance **4f** on MDCK cell culture.

Drug safety is a key aspect in the study of the properties of drugs under develop- ment. In order to determine the maximum concentrations that did not have toxic effects, the cytotoxic activity of six hetero-organic derivatives (**4a**–**f**), was studied. The quantitative assessment of cytotoxicity of the studied substances was performed using the MTT test. The data were recorded 72 h after exposure to the studied com- pounds. Based on the results obtained, the CC_50_ values were calculated (presented in Table 6 and Figure 4, Figure 5, Figure 6, Figure 7, Figure 8, Figure 9 and Figure 10).

Cytotoxicity is the ability of substances to disrupt vital cellular processes, leading to decreased cell viability or even cell death. Cytotoxicity testing using various in vitro methods plays a key role in toxicological safety assessment and the development of new drugs. When exposed to toxic agents, cells rapidly change their morphological characteristics, proliferation rate, metabolic activity, and other functional parameters.

Determining toxicity at the cellular level is a fundamental step in drug safety assessment. It allows us to identify the concentrations of a substance at which cellular damage begins, which is critical for establishing safe dosage ranges. These data form the basis for planning in vivo studies and clinical trials and help to correctly formulate subsequent experimental strategies.

Monitoring of the drug cytotoxicity results in MDCK cell culture was performed after 72 h of incubation. Based on the data obtained, CC50 values (the CC50 concentration of each drug that inhibits cell growth by 50%) were calculated.

The results of the cytotoxic effects of the substances are presented in Table 3 and Figure 4, Figure 5, Figure 6, Figure 7, Figure 8, Figure 9 and Figure 10. It is shown that substances **4c** and **4d** are less toxic compounds, with CC50 values of 0.113 and 0.072 mg/mL, respectively. Substances **4a**, **4e**, **4b**, and **4f** are more cytotoxic to MDCK cell cultures, with CC50 values of 0.052; 0.044; 0.022; and 0.011 mg/mL, respectively.

A comparative analysis of the cytotoxic effects of substances **4a**, **4e**, **4b** and **4f** with substance **4c** on MDCK cells showed that substances **4a** and **4e** are 2.2 and 2.6 times more toxic than **4c**. At the same time, substances **4b** and **4f** have a significantly more pronounced toxic effect, exceeding the cytotoxicity of substance **4c** by 5.1 and 10.3 times.

A similar comparative analysis of the cytotoxic effects of substances **4a**, **4e**, **4b** and **4f** relative to substance **4d** showed that substances **4a** and **4e** have greater cytotoxicity in relation to MDCK cell culture by 1.4 and 1.6 times, and substances **4b** and **4f** by 3.2 and 6.5 times.

The results obtained are necessary for further work with compounds, as they allow potentially dangerous concentrations to be ruled out in advance and subsequent experiments to be planned more accurately.

Thus, based on the results of in vitro microbiological studies of adamantane carboxylates derived from *N-*substituted piperidines, a certain structure–activity relationship can be observed: the presence of *N-*ethoxyethyl, *N-*ethoxypropyl, and *N-*benzyl fragments in the structure of adamantane carboxylates leads to high antimicrobial/antifungal activity.

The in vitro evaluation of antimicrobial activity showed that a number of the synthesized compounds exhibit pronounced antibacterial and/or antifungal effects, indicating the potential of these molecular systems as candidates for further development of anti-infective agents.

As promising compounds for further evaluation against resistant reference and clinical strains of microorganisms, compounds **4d**, **4e**, and **4f** are proposed as agents active against *Staphylococcus aureus*; compounds **4d**, **4e**, and **4f** as agents active against *Escherichia coli*; and compounds **4a**, **4b**, **4d**, **4e**, and **4f** as agents active against *Candida albicans*.

Among all the tested compounds, compound **4f** demonstrated the highest antimicrobial and antifungal efficacy against all reference strains used in the experiment at a concentration of 62.5 µg/mL, exhibiting particularly pronounced fungicidal activity that exceeded the effect of the reference drug fluconazole by 40-fold. In addition, compound **4f** showed high activity against the yeast-like fungus *Cryptococcus neoformans*, resulting in 100% inhibition of its growth.

## 3. Materials and Methods

### 3.1. Chemical Experimental Part

#### 3.1.1. Reagents and Equipment

Piperidin-4-ones (**1a**–**1f**) and adamantane carbonyl chloride were purchased from Sigma-Aldrich (St. Louis, MO, USA). IR spectra were recorded on a Nicolet 5700 spectrometer using KBr pellets. ^1^H and ^13^C NMR spectra were recorded on a JNM-ECA 400 spectrometer (JEOL, Tokyo, Japan), operating at frequencies of 399.78 MHz for ^1^H and 100.53 MHz for ^13^C, using deuterated chloroform (CDCl_3_) and dimethyl sulfoxide (DMSO-d_6_) as solvents. Elemental analysis was carried out using a FlashSmart analyzer (Thermo Fisher Scientific, Waltham, MA, USA). Melting points were determined using an automatic melting point apparatus (Auto Melting Point Apparatus; power supply 220 Vac ±10%, 50/60 Hz). Column and thin-layer chromatography were performed on alumina (Al_2_O_3_) of activity grade III; Rf values of the compounds were determined using this type of plate. The spots were visualized in iodine vapours. The IR and NMR spectra of the synthesized compounds are provided in the Supplementary Materials.

#### 3.1.2. Synthesis of Piperidine-Based Adamantane Carboxylates (**4a**–**f**)

A solution of 0.01 mol of 1-R-4-hydroxypiperidine in chloroform is treated dropwise, under stirring, with a solution of 0.03 mol of adamantane-1-carbonyl chloride in chloroform. During the addition, heating and a change in the colour of the reaction mixture are observed. The mixture is kept for 24 h at room temperature. The resulting white precipitate is filtered, washed with diethyl ether, and the residue is recrystallized from isopropanol.

(1-methylpiperidin-4-yl) adamantane-1-carboxylate (hydrochloride) (**4a**). White powder, yield 55%, melting point 200–203 °C. IR spectrum (KBr), ν, cm^−1^: 1721.3 (C=O). ^1^H NMR spectrum (DMSO-d_6_), δ, ppm (J, Hz): 1.63–1.68 m (6H, H^14ax,16ax,18ax,14eq,16eq,18eq^), 1.74–1.92 m (8H, H^12ax,19ax,20ax,12eq,19eq,20eq,3ax,5ax^), 1.92–2.13 m (5H, H^13,15,17,3eq,5eq^), 2.61–2.70 m (3H, H^7,7,7^), 2.95–3.08 m (2H, H^2ax,6ax^), 3.23–3.38 m (2H, H^2eq,6eq^), 4.74–5.04 m (1H, H^4^), 10.77–11.22 m (1H, H^21^). ^13^C NMR spectrum (DMSO-d_6_), δC, ppm: 27.97 (C^13,15,17^), 36.39 (C^14,16,18^), 38.68 (C^11,19,20,11^), 42.20 and 43.12 (C^7^), 27.07, 29.85 and 32.26 (C^3,5^), 48.86, 49.20, 51.64 and 52.38 (C^2,6^), 63.62 и 64.23 (C^4^), 176.11 (C^9^). COSY NMR spectrum: H^14ax,16ax,18ax^ → H^13,15.17^, H^3ax,5ax^ → H^3eq,5eq^, H^3eq,5eq^ → H^2ax,6ax^, H^3eq,5eq^ → H^2eq,6eq^, H^3eq,5eq^ → H^2ax,6ax^, H^3eq,5eq^ → H^4^, H^2ax,6ax^ → H^2eq,6eq^. HMQC NMR spectrum: H^14,16,18^ →C^14,16,18^, H^12,19,20^ →C^12,19,20^, H^13,15,17^ → C^13,15,17^, H^3ax,5ax^ → C^3,5^, H^3eq,5eq^ → C^3,5^, H^2ax,6ax^ → C^2,6^, H^2eq,6eq^ → C^2,6^, H^7^ → C^7^, H^4^ → C^4^. HMBC NMR spectrum: H^14ax,16ax,18ax^ → C^13,15,17^; H^12ax,19ax,20ax^ → C^13,15,17^, C^14,16,18^, C^12,19,20^, C^9^; H^12eq,19eq,20eq^ → C^13,15,17^, C^14,16,18^, C^9^; H^7^ → C^2,6^; H^2eq,6eq^ → C^4^; H^4^ → C^2,6^. Found, %: Carbon (C) 65.06; Hydrogen (H) 8.99; Nitrogen (N) 4.46; C_17_H_28_NO_2_Cl. Calculated, %: Carbon (C) 64.82; Hydrogen (H) 8.84; Nitrogen (N) 4.63.

(1-propylpiperidin-4-yl) adamantane-1-carboxylate (hydrochloride) (**4b**). White powder, yield 81%, melting point 199–201 °C. IR spectrum (KBr), ν, cm^−1^: 1727.6 (C=O). ^1^H NMR spectrum (DMSO-d_6_), δ, ppm (J, Hz): 0.82–0.86 m (3H, H^9,9,9^),1.62–1.68 m (8H, H^16ax,18ax,20ax,16eq,18eq,20eq,8,8^), 1.74–1.80 m (8H, H^14ax,21ax,22ax,14eq,21eq,22eq,3ax,5ax^), 1.92–2.20 m (5H, H^15,17,19,3eq,5eq^), 2.88–2.97 m (4H, H^7,7,2ax,6ax^), 3.30–3.39 m (2H, H^2eq,6eq^), 4.75–4.89 m (1H, H^4^), 11.13–11.22 m (1H, H^23^). ^13^C NMR spectrum (DMSO-d_6_), δC, ppm: 27.90 (C^15,17,19^), 36.54 (C^16,18,20^), 38.69 (C^14,21,22,13^), 11.51 (C^9^), 17.30 (C^8^), 26.89 (C^3,5^), 47.49 and 49.80 (C^2,6^), 57.00 and 57.65 (C^7^), 64.26 and 67.67 (C^4^), 175.98 and 176.13 (C^11^). COSY NMR spectrum: H^9^ → H^8^, H^8^ → H^7^, H^16ax,18ax,20ax^ → H^15,17.19^, H^3ax,5ax^ → H^3eq,5eq^, H^2ax,6ax^ → H^2eq,6eq^, H^3eq,5eq^ → H^4^. HMQC NMR spectrum: H^9^ → C^9^, H^8^ → C^8^, H^7^ → C^7^, H^4^ → C^4^, H^15,17,19^ → C^15,17,19^, H^16,18,20^ → C^16,18,20^, H^14ax,21ax,22ax^ → C^14,21,22^, H^14eq,21eq,22eq^ → C^14,21,22^, H^3ax,5ax^ → C^3,5^, H^3eq,5eq^ → C^3,5^, H^2ax,6ax^ → C^2,6^, H^2eq,6eq^ → C^2,6^. Found, %: Carbon (C) 66.75; Hydrogen (H) 9.44; Nitrogen (N) 4.10; C_19_H_32_NO_2_Cl. Calculated, %: Carbon (C) 67.12; Hydrogen (H) 9.66; Nitrogen (N) 4.37.

1-(2-hydroxyethylpiperidin-4-yl)adamantane-1-carboxylate (hydrochloride) (**4c**). White powder, yield 76%, melting point 148–150 °C. IR spectrum (KBr), ν, cm^−1^: 1727.0 (C=O). ^1^H NMR spectrum (DMSO-d_6_), δ, ppm (J, Hz): 1.44–1.62 m (7H, H^16ax,18ax,20ax,16eq,18eq,20eq,3ax^), 1.79–1.86 m (7H, H^14ax,21ax,22ax,14eq,21eq,22eq,,5ax^), 1.99–2.32 m (5H, H^15,17,19,3eq,5eq^), 2.95–3.17 m (4H, H^2ax,6ax,2eq,6eq^), 3.29–3.34 m (2H, H^7,7^), 3.58–3.99 m (1H, H^9^), 4.36 m (2H, H^8,8^), 4.73–5.29 m (1H, H^4^), 11.12–11.61 m (1H, H^23^). ^13^C NMR spectrum (DMSO-d_6_), δC, ppm: 27.85 (C^15,17,19^), 30.08, 31.87 (C^3,5^), 36.37 (C^16,18,20^), 38.77 (C^14,21,22,13^), 47.83, 48.20, 50.33 and 51.25 (C^2,6^), 54.09, 54.56 and 55.73 (C^7^), 59.22 and 59.87 (C^8^), and 64.51 (C^4^), 176.34 (C^11^). COSY NMR spectrum: H^16ax,18ax,20ax^ → H^14ax,21ax,22ax^, H^16ax,18ax,20ax^ → H^15,17,19^, H^14ax,21ax,22ax^ → H^15,17,19^, H^7^ → H^8^. HMQC NMR spectrum: H^16,18,20^ → C^16,18,20^, H^14,21,22^ → C^14,21,22^, H^15,17,19^ → C^15,17,19^, H^3ax,5ax^ → C^3,5^, H^3eq,5eq^ → C^3,5^, H^2ax,6ax^ → C^2,6^, H^2eq,6eq^ → C^2,6^, H^7^ → C^7^, H^8^ → C^8^, H^4^ → C^4^. HMBC NMR spectrum: H^16ax,18ax,20ax^ → C^15,17,19^; H^14ax,21ax,22ax^ → C^16,18,20^; H^15,17,19^ → C^14,21,22^. Found, %: Carbon (C) 62.89; Hydrogen (H) 8.79; Nitrogen (N) 4.07; C_18_H_30_NO_3_Cl. Calculated, %: Carbon (C) 62.44; Hydrogen (H) 8.62; Nitrogen (N) 4.15.

1-(2-ethoxyethylpiperidin-4-yl)adamantane-1-carboxylate (hydrochloride) (**4d**). White powder, yield 73%, melting point 154–157 °C. IR spectrum (KBr), ν, cm^−1^: 1722.5 (C=O). ^1^H NMR spectrum (DMSO-d_6_), δ, ppm (J, Hz): 1.06–1.11 m (3H, H^11,11,11^), 1.64 m (7H, H^18ax,20ax,22ax,18eq,20eq,22eq,3ax^), 1.75–1.81 m (7H, H^16ax,23ax,24ax,16eq,23eq,24eq,,5ax^), 1.93–2.21 m (5H, H^17,19,21,3eq,5eq^), 3.00–3.27 m (4H, H^2ax,6ax,2eq,6eq^), 3.41–3.44 m (4H, H^7,7,10,10^), 3.76 m (2H, H^8,8^), 4.72–4.89 m (1H, H^4^), 11.27–11.33 m (1H, H^22^). ^13^C NMR spectrum (DMSO-d_6_), δC, ppm: 15.45 (C^11^), 26.98 (C^3,5^), 27.84 (C^17,19,21^), 36.55 (C^18,20,22^), 38.70 (C^16,23,24,15^), 48.01 and 50.57 (C^2,6^), 55.89 and 55.36 (C^7^), 64.01 (C^4^), 64.75 (C^8^), 66.20 (C^10^), 176.03 (C^13^). COSY NMR spectrum: H^18ax,20ax,22ax^ → H^17,19,21^, H^3ax,5ax^ → H^3eq,5eq^, H^11^ → H^10^, H^7^ → H^8^. HMQC NMR spectrum: H^11^ → C^11^, H^18,20,22^ → C^18,20,22^, H^16ax,23ax,24ax^ → C^16,23,24^, H^16eq,23eq,24eq^ → C^16,23,24^, H^17,19,21^ → C^17,19,21^, H^3ax,5ax^ → C^3,5^, H^3eq,5eq^ → C^3,5^, H^2ax,6ax^ → C^2,6^, H^2eq,6eq^ → C^2,6^, H^7^ → C^7^, H^8^ → C^8^, H^4^ → C^4^, H^10^ → C^10^. HMBC NMR spectrum: H^11^ → C^10^; H^16ax,23ax,24ax^ → C^17,19,21^; H^10^ → C^11^, C^8^; H^8^ → C^10^; H^4^ → C^2,6^. Found, %: Carbon (C) 64.60; Hydrogen (H) 9.21; Nitrogen (N) 3.77; C_20_H_34_NO_3_Cl. Calculated, %: Carbon (C) 64.57; Hydrogen (H) 9.28; Nitrogen (N) 3.72.

1-(3-ethoxypropylpiperidin-4-yl)adamantane-1-carboxylate (hydrochloride) (**4e**). White powder, yield 68%, melting point 164–167 °C. IR spectrum (KBr), ν, cm^−1^: 1721.9 (C=O). ^1^H NMR spectrum (DMSO-d_6_), δ, ppm (J, Hz): 1.05–1.09 m (3H, H^12,12,12^), 1.64 m (7H, H^19ax,21ax,23x,19eq,21eq,23eq,3ax^), 1.76–1.81 m (7H, H^17ax,24ax,25ax,17eq,24eq,25eq,,5ax^), 1.94–2.19 m (7H, H^18,20,22,3eq,5eq,8,8^), 2.89–3.08 m (4H, H^2ax,6ax,7,7^), 3.33–3.42 m (6H, H^9,9,11,11,2eq,6eq^), 4.76–4.91 m (1H, H^4^), 11.01–11.16 m (1H, H^26^). ^13^C NMR spectrum (DMSO-d_6_), δC, ppm: 15.60 (C^12^), 24.35 (C^8^), 26.98 (C^3,5^), 27.84 (C^18,20,22^), 36.40 (C^19,21,23^), 38.67 (C^17,24,25,16^), 47.65 and 49.99 (C^2,6^), 53.57 and 54.25 (C^7^), 64.08 (C^4^), 66.04 (C^11^), 67.34 and 67.77 (C^9^), 176.11 and 176.35 (C^14^). COSY NMR spectrum: H^19ax,21ax,23ax^ → H^18,20,22^, H^17ax,24ax,25ax^ → H^18,20,22^, H^3ax,5ax^ → H^3eq,5eq^, H^2ax,6ax^ → H^2eq,6eq^, H^8^ → H^7^, H^12^ → H^11^, H^9^ → H^11^. HMQC NMR spectrum: H^12^ → C^12^, H^9,11^ → C^9,11^, H^8^ → C^8^, H^7^ → C^7^, H^18,20,22^ → C^18,20,22^, H^17ax,24ax,25ax^ → C^17,24,25^, H^17eq,24eq,25eq^ → C^17,24,25^, H^3ax,5ax^ → C^3,5^, H^3eq,5eq^ → C^3,5^, H^2ax,6ax^ → C^2,6^, H^2eq,6eq^ → C^2,6^, H^4^ → C^4^. HMBC NMR spectrum: H^12^ → C^11^; H^17ax,24ax,25ax^ → C^19,21,23^, C^18,20,22^; H^19ax,21ax,23ax^ → C^18,20,22^; H^8^ → C^7^, C^9^; H^11^ → C^12^, C^8^, C^7^. Found, %: Carbon (C) 65.35; Hydrogen (H) 9.41; Nitrogen (N) 3.63; C_21_H_36_NO_3_Cl. Calculated, %: Carbon (C) 65.72; Hydrogen (H) 9.53; Nitrogen (N) 3.61.

(1-benzylpiperidin-4-yl)adamantane-1-carboxylate (hydrochloride) (**4f**). White powder, yield 45%, melting point 203–205 °C. IR spectrum (KBr), ν, cm^−1^: 1722.2 (C=O). ^1^H NMR spectrum (DMSO-d_6_), δ, ppm (J, Hz): 1.58–1.66 m (6H, H^20ax,22ax,24ax,20eq,22eq,24eq^), 1.72–1.74 m (6H, H^18ax,25ax,26ax,18eq,25eq,26eq^), 1.81–1.99 m (5H, H^19,21,23,3ax,5ax^), 2.09–2.16 m (2H, H^3eq,5eq^), 2.87–3.06 m (2H, H^2ax,6ax^), 3.20–3.28 m (2H, H^2eq,6eq^), 4.21–4.31 m (2H, H^7,7^), 4.72–4.88 m (1H, H^4^), 7.41–7.42 m (3H, H^9,11,13^), 7.56–7.62 m (2H, H^10,12^), 11.13–11.24 m (1H, H^27^). ^13^C NMR spectrum (DMSO-d_6_), δC, ppm: 26.81 (C^3,5^), 27.77 (C^19,21,23^), 36.53 (C^20,22,24^), 38.68 (C^18,25,26^), 38.78 (C^17^), 46.76 (C^2,6^), 58.62 and 58.97 (C^7^), 64.08 (C^4^), 129.29 (C^9,13^), 129.94 (C^11^), 131.91 (C^8^), 132.28 (C^10,12^), 175.91 (C^15^). COSY NMR spectrum: H^20ax,22ax,24ax^ → H^19,21,23^, H^20eq,22eq,24eq^ → H^19,21,23^, H^3ax,5ax^ → H^3eq,5eq^, H^3eq,5eq^ → H^2ax,6ax^, H^2ax,6ax^ → H^2eq,6eq^, H^9,13^ → H^10,12^. HMQC NMR spectrum: H^20,22,24^ → C^20,22,24^, H^18,25,26^ → C^18,25,26^, H^19,21,23^ → C^19,21,23^, H^3ax,5ax^ → C^3,5^, H^3eq,5eq^ → C^3,5^, H^2ax,6ax^ → C^2,6^, H^2eq,6eq^ → C^2,6^, H^7^ → C^7^, H^4^ → C^4^, H^10,12^ → C^10,12^, H^11^ → C^11^, H^9,13^ → C^9,13^. HMBC NMR spectrum: H^20ax,22ax,24ax^ → C^19,21,23^, C^18,25,26^; H^18,25,26^ → C^19,21,23^, C^20,22,24^; H^7^ → C^2,6^, C^9,13^; H^9,13^ → C^10,12^; H^10,12^ → C^7^, C^9,13^. Found, %: Carbon (C) 70.83; Hydrogen (H) 8.27; Nitrogen (N) 3.59; C_23_H_32_NO_2_Cl. Calculated, %: Carbon (C) 70.61; Hydrogen (H) 8.32; Nitrogen (N) 3.64.

### 3.2. Biological Experimental Part

#### 3.2.1. The Cell Culture

The monolayer transient cell culture MDCK (Madin Darby Canine Kidney cells) obtained from the Laboratory of Cell Biotechnology of the Research Institute of Biological Safety Problems at the National Center of Biotechnology of the Ministry of Education and Science of the Republic of Kazakhstan was used.

#### 3.2.2. Determination of Cytotoxicity of Substances In Vitro

The cytotoxicity of the investigated substances in vitro was assessed, using the MTT test. The plates were incubated in the thermostat at 37 °C and 5.0% CO_2_. After 72 h, optical density was recorded on a Tecan Sunrise RC. 4 microplate reader (Austria) at a wavelength of 540 nm for the main filter and 620 nm for the reference filter. The CC_50_ value was calculated using the following Equation (1):

(1) $${\mathrm{CC}}_{50}=\frac{X_{1}-50}{X_{1}-X_{2}}\times \left(Mx_{2}-Mx_{1}\right)+Mx_{1}$$

where $X_{1}$—more than 50% of surviving cells; $X_{2}$—less than 50% of surviving cells; $Mx_{1}$—concentration of substance where more than 50% of cells survived; $Mx_{2}$—concentration of substance where less than 50% of cells survived.

#### 3.2.3. Statistics

The results of the conducted quantitative studies were processed using the one-way ANOVA method of one-factor analysis of variance, with further analysis using the GraphPad Prism 5 application software package.

#### 3.2.4. Determination of Antimicrobial (Antibacterial and Antifungal) Activity In Vitro

The study was carried out using the broth microdilution method with twofold serial dilutions in liquid growth media. Mueller–Hinton broth was used to determine antimicrobial activity, while Sabouraud broth was used to determine fungicidal activity. The media used for susceptibility testing were standardized and recommended by CLSI.

Into Eppendorf-type tubes, 0.5 mL of the corresponding growth medium was added. Then, 0.5 mL of the test compound solution was added to the first tube in the series. After mixing, a series of twofold dilutions of the tested substances was prepared. Each series included four control tubes: medium control, solvent control (negative control, used to verify the effect of ethanol and DMSO at different concentrations on cell viability), positive control (reference drug), and culture growth control. A 0.05 mL aliquot of the microbial test strain suspension was added to all tubes except the medium control.

The samples inoculated with bacterial cultures were incubated for 18–24 h at 37 ± 1 °C. After the incubation period, subculturing was performed onto Petri dishes containing solid nutrient medium. The inoculated plates were then placed in an incubator and incubated for 18–24 h at 37 ± 1 °C.

The samples inoculated with C. albicans were incubated for 48–72 h at 22 ± 1 °C. After the incubation period, subculturing onto Petri dishes with solid nutrient medium was performed. Following inoculation, the plates were placed in an incubator and kept for 48–72 h at 22 ± 1 °C.

All experiments were performed in triplicate.

The results were evaluated based on the presence or absence of visible microbial growth on the surface of the solid medium. The minimum bactericidal concentration (MBC) was defined as the lowest concentration that resulted in complete inhibition of microbial growth. The minimum bacteriostatic concentration was defined as the concentration that inhibited microbial growth.

#### 3.2.5. Determination of Antimicrobial Activity by the Serial Dilution Method

All microorganisms were obtained from the American Type Culture Collection (ATCC), Manassas, Virginia. The biological activity studies were performed according to a modified version of the Clinical and Laboratory Standards Institute (CLSI, formerly NCCLS) protocol. Optical density measurement was used to monitor microbial growth.

The samples (dissolved in DMSO) were serially diluted in a 20% DMSO solution prepared in physiological saline and transferred (10 μL per well) in duplicate into 96-well microtiter plates. Inocula were prepared to achieve the desired number of colony-forming units per millilitre (CFU/mL) by adjusting microbial suspensions to an OD_630_ (optical density) reading. The final concentration of the test samples corresponded to 1/100 of the residual DMSO concentration. Control samples were included in each assay. IC_50_ values (the concentration at which microbial growth is inhibited by 50%) were calculated using XLfit 4.2 software (IDBS, Alameda, CA, USA) with the appropriate Model 201.

## 4. Conclusions

Based on a series of (N-alkyl-, N-alkoxyalkyl-, N-hydroxyalkyl-, N-arylalkyl-) piperidin-4-ones, the corresponding secondary alcohols were obtained by reduction with sodium borohydride in absolute isopropanol, followed by acylation of these alcohols with adamantane-1-carbonyl chloride to synthesize a series of new adamantane-1-carboxylate esters.

The synthesized compounds were then characterized using various spectroscopic techniques like 1H and 13C NMR, as well as advanced techniques like COSY, HMQC, and HMBC. This study highlights the importance of structural characterization in identifying novel compounds with valuable biological activities.

The synthesized compounds were investigated for their cytotoxic activities. It was shown that among all the tested substances, compound **4c** exhibited the lowest cytotoxicity toward MDCK cells.

Based on the microbiological studies of piperidine-based adamantane carboxylates conducted in vitro, the following compounds were identified as promising candidates for further evaluation against resistant museum and clinical microbial strains: **4d**, **4e**, and **4f** as agents active against *Staphylococcus aureus*; **4d**, **4e**, and **4f** as agents active against *Escherichia coli*; and **4a**, **4b**, **4d**, **4e**, and **4f** as agents active against *Candida albicans*. Among all the tested compounds, compound **4f** demonstrated the highest antimicrobial and antifungal effectiveness against all museum strains used in the experiment at a concentration of 62.5 μg/mL, showing particularly pronounced fungicidal activity, exceeding the effect of the reference drug fluconazole by a factor of 40. Moreover, compound **4f** exhibited strong activity against the yeast-like fungus *Cryptococcus neoformans*, resulting in 100% growth inhibition of this microorganism.

Thus, the presence of N-ethoxyethyl, N-ethoxypropyl and N-benzyl fragments in the structure of piperidine-based adamantane carboxylates resulted in high antimicrobial activity. It is noteworthy that combining an N-benzyl fragment and an adamantane fragment within a single molecule led to pronounced antimicrobial and antifungal activities. The in vitro evaluation of antimicrobial activity showed that a number of the obtained compounds exhibit marked antibacterial and/or antifungal effects, indicating the potential of these molecular systems as candidates for further development of anti-infective agents.

## Figures and Tables

**Table 1:** Evaluation of the Minimum Bactericidal Concentration of Compounds (**4a**–**f**) Against Staphylococcus aureus ATCC 6538-P.

Concentration, μg/mL	Test Strain *Staphylococcus aureus* ATCC 6538-P						
	4a	4b	4c	4d	4e	4f	AMP
2000	-	-	+	-	-	-	-
1000	+	+	+	-	-	-	+
500	+	+	+	-	-	-	+
250	+	+	+	-	-	-	+
125	+	+	+	-	+ *	-	+
62.5	+	+	+ *	+ *	+	-	+
31.25	+	+	+	+	+	+ *	+
15.6	+	+	+	+	+	+	+
7.8	+	+	+	+	+	+	+
3.9	+	+	+	+	+	+	+
2	+	+	+	+	+	+	+
1	+	+	+	+	+	+	+
0.5	+	+	+	+	+	+	+
0.25	+	+	+	+	+	+	+
0.125	+	+	+	+	+	+	+

Notes: 1. AMP ampicillin; 2. “+”—growth present; 3. “-”—no growth; 4. “*”—bacteriostatic effect.

**Table 2:** Evaluation of the Minimum Bactericidal Concentration Against *Escherichia coli* ATCC 8739.

Concentration, μg/mL	Test Strain *Escherichia coli* ATCC 8739						
	4a	4b	4c	4d	4e	4f	AMP
2000	-	-	+	-	-	-	-
1000	+	+	+	-	-	-	+
500	+	+	+	-	+ *	-	+
250	+	+	+	+ *	+	-	+
125	+	+	+	+	+	-	+
62.5	+	+	+	+	+	-	+
31.25	+	+	+	+	+	+	+
15.6	+	+	+	+	+	+	+
7.8	+	+	+	+	+	+	+
3.9	+	+	+	+	+	+	+
2	+	+	+	+	+	+	+
1	+	+	+	+	+	+	+
0.5	+	+	+	+	+	+	+
0.25	+	+	+	+	+	+	+
0.125	+	+	+	+	+	+	+

Notes: 1. AMP ampicillin; 2. “+”—growth present; 3. “-”—no growth; 4. “*”—bacteriostatic effect.

**Table 3:** Evaluation of the Minimum Fungicidal Concentration Against *Candida albicans* ATCC 10231.

Concentration, μg/mL	Test Strain *Candida albicans* ATCC 10231						
	4a	4b	4c	4d	4e	4f	Fluc
2000	-	-	+	-	-	-	+
1000	-	-	+	-	-	-	+
500	-	-	+	-	-	-	+
250	+	+	+	-	+	-	+
125	+	+	+	+	+	-	+
62.5	+	+	+	+	+	-	+
31.25	+	+	+	+	+	+	+
15.6	+	+	+	+	+	+	+
7.8	+	+	+	+	+	+	+
3.9	+	+	+	+	+	+	+
2	+	+	+	+	+	+	+
1	+	+	+	+	+	+	+
0.5	+	+	+	+	+	+	+
0.25	+	+	+	+	+	+	+
0.125	+	+	+	+	+	+	+

Notes: 1. Fluc—fluconazole; 2. “+”—growth present, 3. “-”—no growth.

**Table 4:** Results of the Primary Test (presented as percentages of microbial growth inhibition).

Sample Name	Sample Code	*Candida albicans*	*Aspergillus fumigatus*	*Cryptococcus neoformans*	*MRS*	*Escherichia coli*	*Pseudomonas aeruginosa*	*Klebsiella pneumoniae*	*VRE*	*Test Conc*
Fluconazole	FLU	82	0	87	0	1	5	6	10	100 μg/mL
Amphotericin B (New Lot)	AMB	97	96	98	0	0	0	2	0	100 μg/mL
Ciprofloxacin (New Lot)	CIPRO	19	0	82	0	97	100	15	0	10 μg/mL
Vancomycin	Vanco	0	0	21	99	51	0	19	14	100 μg/mL
Methicillin	METH	5	4	6	99	15	0	15	12	100 μg/mL
Cefotaxime	CEFO	5	0	46	98	36	0	5	8	100 μg/mL
Meropenem	MERO	6	7	33	46	97	33	52	7	100 μg/mL
Compound **4f**	**4f**	24	8	100	0	100	0	0	0	20 μg/mL

**Table 5:** Results of the Secondary Test (Half-maximal Inhibitory Concentration, i.e., the concentration at which microbial growth is inhibited by 50%).

Sample Name	Sample Code	*Candida albicans* IC50	*Aspergillus fumigatus* IC50	*Cryptococcus neoformans* IC50	*MRS* IC50	*Escherichia coli* IC50	*Pseudomonas aeruginosa* IC50	*Klebsiella pneumoniae* IC50	*VRE* IC50	*Test Conc*
Fluconazole	FLU	<0.1	>100	0.76	>100	>100	>100	>100	>100	100–4 μg/mL
Amphotericin B (New Lot)	AMB	0.11	0.36	0.44	>100	>100	>100	>100	>100	100–4 μg/mL
Ciprofloxacin (New Lot)	CIPRO	>10	>10	0	>10	<0.01	0.16	>10	>10	10–0.4 μg/mL
Vancomycin	Vanco	>100	>100	>100	0.45	98.9	>100	>100	>100	100–4 μg/mL
Methicillin	METH	>100	>100	>100	46.77	>100	>100	>100	>100	100–4 μg/mL
Cefotaxime	CEFO	>100	>100	>100	23.82	>100	>100	>100	>100	100–4 μg/mL
Meropenem	MERO	>100	>100	>100	51.1	67.94	>100	>100	>100	100–4 μg/mL
Compound **4f**	**4f**	>20	>20	<0.8	>20	>20	>20	>20	>20	20–0.8 μg/mL

**Table 6:** Evaluation of cytotoxic action of the investigated Test item on culture cells MDCK.

Name of Substance	CC_50_ ± SD, mg/mL
**4a**	0.052 ± 0.008
**4b**	0.022 ± 0.005
**4c**	0.113 ± 0.004
**4d**	0.071 ± 0.007
**4e**	0.044 ± 0.003
**4f**	0.011 ± 0.0
Name of Substance	CC_50_ ± SD, mg/mL

## Data Availability

The datasets used and/or analyzed during the present study are available from the corresponding author upon reasonable request.

## Supplementary Materials

The following supporting information can be downloaded at: https://www.mdpi.com/article/10.3390/molecules31030439/s1, Table S1: Spectroscopic and physical data; Figure S1: IR spectra of compound **4a**; Figure S2: IR spectra of compound **4b**; Figure S3: IR spectra of compound **4c**; Figure S4: IR spectra of compound **4d**; Figure S5: IR spectra of compound **4e**; Figure S6: IR spectra of compound **4f**; Figure S7: ^1^H NMR spectra of compound **4a** (in DMSO); Figure S8: ^13^C NMR spectra of compound **4a** (in DMSO); Figure S9: COSY of compound **4a** (in DMSO); Figure S10: HMQC of compound **4a** (in DMSO); Figure S11: HMBC of compound **4a** (in DMSO); Figure S12: ^1^H NMR spectra of compound **4b** (in DMSO); Figure S13: ^13^C NMR spectra of compound **4b** (in DMSO); Figure S14: COSY of compound **4b** (in DMSO); Figure S15: HMQC of compound **4b** (in DMSO); Figure S16: ^1^H NMR spectra of compound **4c** (in DMSO); Figure S17: ^13^C NMR spectra of compound **4c** (in DMSO); Figure S18: COSY spectra of compound **4c** (in DMSO); Figure S19: HMQC of compound **4c** (in DMSO); Figure S20: HMBC of compound **4c** (in DMSO); Figure S21: ^1^H NMR spectra of compound **4d** (in DMSO); Figure S22: ^13^C NMR spectra of compound **4d** (in DMSO); Figure S23: COSY of compound **4d** (in DMSO); Figure S24: HMQC of compound **4d** (in DMSO); Figure S25: HMBC of compound **4d** (in DMSO); Figure S26: ^1^H NMR spectra of compound **4e** (in DMSO); Figure S27: ^13^C NMR spectra of compound **4e** (in DMSO); Figure S28: COSY of compound **4e** (in DMSO); Figure S29: HMQC of compound **4e** (in DMSO); Figure S30: HMBC of compound **4e** (in DMSO); Figure S31: ^1^H NMR spectra of compound **4f** (in DMSO); Figure S32: ^13^C NMR spectra of compound **4f** (in DMSO); Figure S33: COSY of compound **4f** (in DMSO); Figure S34: HMQC of compound **4f** (in DMSO); Figure S35: HMBC of compound **4f** (in DMSO).
