# Supplementary material for: Synthesis, Antimicrobial Activity and Cytotoxicity of Novel (Piperidin-4-yl)adamantane-1-carboxylate N-Substituted Derivatives
Source: Molecules. 2026 Jan 27;31(3):439. doi: 10.3390/molecules31030439 (PMC12899892; doi:10.3390/molecules31030439)
Supplement: Supplementary file 1 [file molecules-31-00439-s001.zip › molecules-4045809-Supplementary.pdf]

Supporting information

« Synthesis, Antimicrobial Activity and Cytotoxicity of Novel (Piperidin-4-yl)adamantane-1-carboxylate N-Substituted Derivatives»

Table of contents

Experimental 1
General Information..1
Experimental Procedures 1
Spectroscopic and physical data 2
Copies of IR Spectra of Products 6
Copies of NMR Spectra of Products 9

Experimental

General Information

Piperidin-4-ones (1a–1f) and adamantane carbonyl chloride were purchased from Sigma-Aldrich (St. Louis, MO, USA). IR spectra were recorded on a Nicolet 5700 spec-trometer using KBr pellets. ^{1}H and ^{13}C NMR spectra were recorded on a JNM-ECA 400 spectrometer (JEOL, Tokyo, Japan), operating at frequencies of 399.78 MHz for ^{1}H and 100.53 MHz for ^{13}C, using deuterated chloroform ($CDCl_3$) and dimethyl sulfoxide (DMSO-d_6) as solvents. Elemental analysis was carried out using a FlashSmart analyzer (Thermo Fisher Scientific, USA). Melting points were determined using an automatic melting point apparatus (Auto Melting Point Apparatus; power supply 220 Vac ±10%, 50/60 Hz). Column and thin-layer chromatography were performed on alumina (Al_2O_3) of activity grade III; Rf values of the compounds were determined using this type of plate. The spots were visualized in iodine vapors. The IR and NMR spectra of the synthesized compounds are provided in the Supplementary Materials.

Experimental Procedures

(1-methylpiperidin-4-yl) adamantane-1-carboxylate (hydrochloride) (4a), (fig. S1, fig. S7- fig. S11). A solution of 5.18 g (0.0261 mol) of adamantane carbonyl chloride **(3)** in chloroform was added dropwise to a stirred solution of 1 g (0.0087 mol) of 1-methyl-4-hydroxypiperidine **(1a)**. During the addition, heating of the reaction mixture was observed. The mixture was maintained at room temperature for 24 h. The resulting precipitate was washed with diethyl ether, and the residue was recrystallized from isopropanol. Yield: 1.5 g (55 % of the theoretical) (1-methylpiperidin-4-yl) adamantane-1-carboxylate (hydrochloride) **(4a)**, m.p. 200–203°C, Rf 0.84 (Al_2O_3, eluent — benzene : dioxane = 4:1).

(1-propylpiperidin-4-yl) adamantane-1-carboxylate (hydrochloride) (4b), (fig. S2, fig. S12- fig. S15). A solution of 2.9 g (0.03 mol) of adamantane carbonyl chloride **(3)** in chloroform was added dropwise, with stirring, to a solution of 1.5 g (0.01 mol) of 1-propyl-4-hydroxypiperidine **(1b)** in chloroform. Heating and a color change of the reaction mixture were observed during the addition. The mixture was kept at room temperature for 2 days. The resulting white precipitate was filtered off, washed with diethyl ether, and the residue was recrystallized from isopropanol. Yield: 2.9 g (81 % of the theoretical) (1-propylpiperidin-4-yl) adamantane-1-carboxylate (hydrochloride) **(4b)**, m.p. 199–201°C, Rf 0.81 (Al_2O_3, eluent — benzene : dioxane = 4:1).

1-(2-hydroxyethylpiperidin-4-yl)adamantane-1-carboxylate (hydrochloride) (4c), (fig. S3, fig. S16- fig. S20). A solution of 1.01 g (0.0051 mol) of adamantane carbonyl chloride **(3)** in chloroform was added dropwise, with stirring, to a solution of 0.74 g (0.0051 mol) of 1-(2-hydroxyethyl)-4-hydroxypiperidine **(1c)** in chloroform. Heating and a color change of the reaction mixture were observed during the addition. The mixture was maintained at room temperature for 24 h. The resulting white precipitate was filtered off, washed with diethyl ether, and the residue was recrystallized from isopropanol. Yield: 1.01 g (76 % of the theoretical) 1-(2-

hydroxyethylpiperidin-4-yl)adamantane-1-carboxylate (hydrochloride) **(4c)**, m.p. 148–150°C, Rf 0.85 (Al_2O_3, eluent — benzene : dioxane = 4:1).

1-(2-ethoxyethylpiperidin-4-yl)adamantane-1-carboxylate (hydrochloride) (4d), (fig. S4, fig. S21- fig. S25). A solution of 3.44 g (0.0174 mol) of adamantane carbonyl chloride **(3)** in chloroform was added dropwise, with stirring, to a solution of 1.0 g (0.0058 mol) of 1-(2-ethoxyethyl)-4-hydroxypiperidine **(1d)** in chloroform. Heating and a color change of the reaction mixture were observed during the addition. The mixture was kept at room temperature for 12 h. The resulting white precipitate was filtered off, washed with diethyl ether, and the residue was recrystallized from isopropanol. Yield: 1.58 g (73 % of the theoretical) 1-(2-ethoxyethylpiperidin-4-yl)adamantane-1-carboxylate (hydrochloride) **(4d)**, m.p. 154–157°C, Rf 0.84 (Al_2O_3, eluent — benzene : dioxane = 4:1).

1-(3-ethoxypropylpiperidin-4-yl)adamantane-1-carboxylate (hydrochloride) (4e), (fig. S5, fig. S26- fig. S30). A solution of 3.18 g (0.0160 mol) of adamantane carbonyl chloride **(3)** in chloroform was added dropwise, with stirring, to a solution of 1.0 g (0.00534 mol) of 1-(3-ethoxypropyl)-4-hydroxypiperidine **(1e)** in chloroform. Heating and a color change of the reaction mixture were observed during the addition. The mixture was kept at room temperature for 12 h. The resulting white precipitate was filtered off, washed with diethyl ether, and the residue was recrystallized from isopropanol. Yield: 1.4 g (68 % of the theoretical) 1-(3-ethoxypropylpiperidin-4-yl)adamantane-1-carboxylate (hydrochloride) **(4e)**, m.p. 164–167°C, Rf 0.84 (Al_2O_3, eluent — benzene : dioxane = 4:1).

(1-benzylpiperidin-4-yl)adamantane-1-carboxylate (hydrochloride) (4f), (fig. S6, fig. S31- fig. S35). A solution of 3 g (0.0157 mol) of 1-benzyl-4-hydroxypiperidine **(1f)** in chloroform was mixed with a solution of 3.43 g (0.0173 mol) of adamantane carbonyl chloride **(3)** in chloroform. A slight heating of the reaction mixture was observed. The mixture was kept at room temperature for 12 h. The resulting white precipitate was filtered off, washed with diethyl ether, and recrystallized from isopropanol. Yield: 2.76 g (45 % of the theoretical) (1-benzylpiperidin-4-yl)adamantane-1-carboxylate (hydrochloride) **(4f)**, m.p. 203–205°C, Rf 0.91 (Al_2O_3, eluent — benzene : dioxane = 4:1).

Table 1. Spectroscopic and physical data

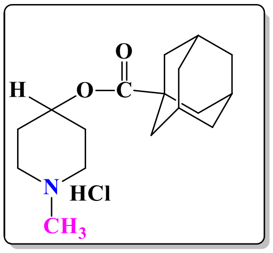<br><br>**4a**<br>**Fig.S1, Fig.S7- Fig.S11**<br>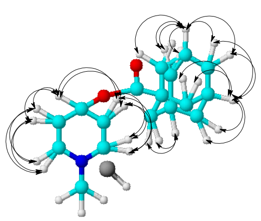<br>COSY<br>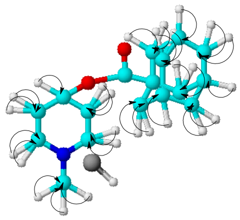<br>HMQC	**(1-methylpiperidin-4-yl) adamantane-1-carboxylate (hydrochloride) (4a).**<br>White powder, yield 55 %, melting point 200-203°C.<br>IR spectrum (KBr), ν, см$^{-1}$: 1721.3 (C=O).<br><br>^{1}H NMR spectrum (DMSO-d$_6$), δ, ppm (J, Hz): 1.63–1.68 m (6H, $H^{14ax,16ax,18ax,14eq,16eq,18eq}$), 1.74–1.92 m (8H, $H^{12ax,19ax,20ax,12eq,19eq,20eq,3ax,5ax}$), 1.92–2.13 m (5H, $H^{13,15,17,3eq,5eq}$), 2.61–2.70 m (3H, $H^{7,7,7}$), 2.95–3.08 m (2H, $H^{2ax,6ax}$), 3.23–3.38 m (2H, $H^{2eq,6eq}$), 4.74–5.04 m (1H, H^4), 10.77–11.22 m (1H, H^{21}).<br>^{13}C NMR spectrum (DMSO-d$_6$), δC, ppm: 27.97 ($C^{13,15,17}$), 36.39 ($C^{14,16,18}$), 38.68 ($C^{11,19,20,11}$), 42.20 and 43.12 (C^7), 27.07, 29.85 and 32.26 ($C^{3,5}$), 48.86, 49.20, 51.64 and 52.38 ($C^{2,6}$), 63.62 и 64.23 (C^4), 176.11 (C^9).<br>COSY NMR spectrum: $H^{14ax,16ax,18ax}$→$H^{13,15.17}$, $H^{3ax,5ax}$→$H^{3eq,5eq}$, $H^{3eq,5eq}$→$H^{2ax,6ax}$, $H^{3eq,5eq}$→$H^{2eq,6eq}$, $H^{3eq,5eq}$→$H^{2ax,6ax}$, $H^{3eq,5eq}$→H^4, $H^{2ax,6ax}$→$H^{2eq,6eq}$.<br>HMQC NMR spectrum: $H^{14,16,18}$→$C^{14,16,18}$, $H^{12,19,20}$→$C^{12,19,20}$, $H^{13,15,17}$→$C^{13,15,17}$, $H^{3ax,5ax}$→$C^{3,5}$, $H^{3eq,5eq}$→$C^{3,5}$, $H^{2ax,6ax}$→$C^{2,6}$, $H^{2eq,6eq}$→$C^{2,6}$, H^7→C^7, H^4→C^4.<br>HMBC NMR spectrum: $H^{14ax,16ax,18ax}$→$C^{13,15,17}$; $H^{12ax,19ax,20ax}$→$C^{13,15,17}$, $C^{14,16,18}$, $C^{12,19,20}$, C^9; $H^{12eq,19eq,20eq}$→$C^{13,15,17}$, $C^{14,16,18}$, C^9; H^7→$C^{2,6}$; $H^{2eq,6eq}$→C^4; H^4→$C^{2,6}$.<br><br>Found, %: Carbon (C) 65,06; Hydrogen (H) 8,99; Nitrogen (N) 4,46; $C_{17}H_{28}ClNO_2$.

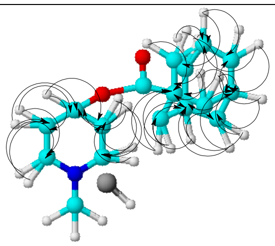

HMBC

Calculated, %: Carbon (C) 64,82; Hydrogen (H) 8,84; Nitrogen (N) 4,63.

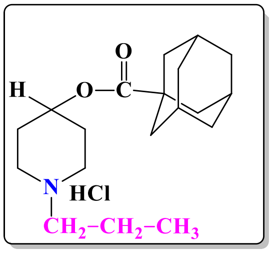

4b

Fig.S2, Fig.S12- Fig.S15

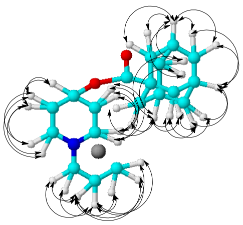

COSY

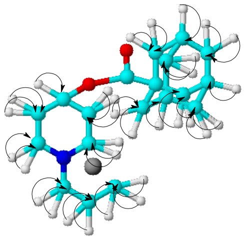

HMQC

(1-propylpiperidin-4-yl) adamantane-1-carboxylate (hydrochloride) (4b).

White powder, yield 81 %, melting point 199-201°C.

IR spectrum (KBr), ν, см$^{-1}$: 1727.6 (C=O).

^{1}H NMR spectrum (DMSO-d$_6$), δ, ppm (J, Hz): 0.82-0.86 m (3H, $H^{9,9,9}$),1.62-1.68 m (8H, $H^{16ax,18ax,20ax,16eq,18eq,20eq,8,8}$), 1.74-1.80 m (8H, $H^{14ax,21ax,22ax,14eq,21eq,22eq,3ax,5ax}$), 1.92-2.20 m (5H, $H^{15,17,19,3eq,5eq}$), 2.88-2.97 m (4H, $H^{7,7,2ax,6ax}$), 3.30-3.39 m (2H, $H^{2eq,6eq}$), 4.75-4.89 m (1H, H^{4}), 11.13-11.22 m (1H, H^{23}).

^{13}C NMR spectrum (DMSO-d$_6$), δC, ppm: 27.90 ($C^{15,17,19}$), 36.54 ($C^{16,18,20}$), 38.69 ($C^{14,21,22,13}$), 11.51 (C^{9}), 17.30 (C^{8}), 26.89 ($C^{3,5}$), 47.49 and 49.80 ($C^{2,6}$), 57.00 and 57.65 (C^{7}), 64.26 and 67.67 (C^{4}), 175.98 and 176.13 (C^{11}).

COSY NMR spectrum: $H^{9}\rightarrow H^{8}$, $H^{8}\rightarrow H^{7}$, $H^{16ax,18ax,20ax}\rightarrow H^{15,17.19}$, $H^{3ax,5ax}\rightarrow H^{3eq,5eq}$, $H^{2ax,6ax}\rightarrow H^{2eq,6eq}$, $H^{3eq,5eq}\rightarrow H^{4}$.

HMQC NMR spectrum: $H^{9}\rightarrow C^{9}$, $H^{8}\rightarrow C^{8}$, $H^{7}\rightarrow C^{7}$, $H^{4}\rightarrow C^{4}$, $H^{15,17,19}\rightarrow C^{15,17,19}$, $H^{16,18,20}\rightarrow C^{16,18,20}$, $H^{14ax,21ax,22ax}\rightarrow C^{14,21,22}$, $H^{14eq,21eq,22eq}\rightarrow C^{14,21,22}$, $H^{3ax,5ax}\rightarrow C^{3,5}$, $H^{3eq,5eq}\rightarrow C^{3,5}$, $H^{2ax,6ax}\rightarrow C^{2,6}$, $H^{2eq,6eq}\rightarrow C^{2,6}$.

Found, %: Carbon (C) 66,75; Hydrogen (H) 9,44; Nitrogen (N) 4,10; $C_{19}H_{32}ClNO_2$.

Calculated, %: Carbon (C) 67,12; Hydrogen (H) 9,66; Nitrogen (N) 4,37.

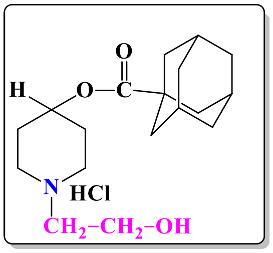

4c

Fig.S3, Fig.S16- Fig.S20

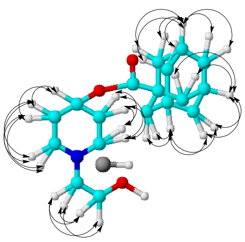

COSY

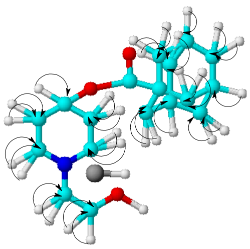

HMQC

1-(2-hydroxyethylpiperidin-4-yl)adamantane-1-carboxylate (hydrochloride) (4c).

White powder, yield 76 %, melting point 148-150°C.

IR spectrum (KBr), ν, см$^{-1}$: 1727.0 (C=O).

^{1}H NMR spectrum (DMSO-d$_6$), δ, ppm (J, Hz): 1.44-1.62 m (7H, $H^{16ax,18ax,20ax,16eq,18eq,20eq,3ax}$), 1.79-1.86 m (7H, $H^{14ax,21ax,22ax,14eq,21eq,22eq,,5ax}$), 1.99-2.32 m (5H, $H^{15,17,19,3eq,5eq}$), 2.95-3.17 m (4H, $H^{2ax,6ax,2eq,6eq}$), 3.29-3.34 m (2H, $H^{7,7}$), 3.58-3.99 m (1H, H^{9}), 4.36 m (2H, $H^{8,8}$), 4.73-5.29 m (1H, H^{4}), 11.12-11.61 m (1H, H^{23}).

^{13}C NMR spectrum (DMSO-d$_6$), δC, ppm: 27.85 ($C^{15,17,19}$), 30.08, 31.87 ($C^{3,5}$), 36.37 ($C^{16,18,20}$), 38.77 ($C^{14,21,22,13}$), 47.83, 48.20, 50.33 and 51.25 ($C^{2,6}$), 54.09, 54.56 and 55.73 (C^{7}), 59.22 and 59.87 (C^{8}), and 64.51 (C^{4}), 176.34 (C^{11}).

COSY NMR spectrum: $H^{16ax,18ax,20ax}\rightarrow H^{14ax,21ax,22ax}$, $H^{16ax,18ax,20ax}\rightarrow H^{15,17,19}$, $H^{14ax,21ax,22ax}\rightarrow H^{15,17,19}$, $H^{7}\rightarrow H^{8}$.

HMQC NMR spectrum: $H^{16,18,20}\rightarrow C^{16,18,20}$, $H^{14,21,22}\rightarrow C^{14,21,22}$, $H^{15,17,19}\rightarrow C^{15,17,19}$, $H^{3ax,5ax}\rightarrow C^{3,5}$, $H^{3eq,5eq}\rightarrow C^{3,5}$, $H^{2ax,6ax}\rightarrow C^{2,6}$, $H^{2eq,6eq}\rightarrow C^{2,6}$, $H^{7}\rightarrow C^{7}$, $H^{8}\rightarrow C^{8}$, $H^{4}\rightarrow C^{4}$.

HMBC NMR spectrum: $H^{16ax,18ax,20ax}\rightarrow C^{15,17,19}$; $H^{14ax,21ax,22ax}\rightarrow C^{16,18,20}$; $H^{15,17,19}\rightarrow C^{14,21,22}$.

Found, %: Carbon (C) 62,89; Hydrogen (H) 8,79; Nitrogen (N) 4,07; $C_{18}H_{30}ClNO_3$.

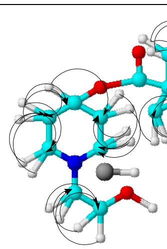

HMBC

Calculated, %: Carbon (C) 62,44; Hydrogen (H) 8,62; Nitrogen (N) 4,15.

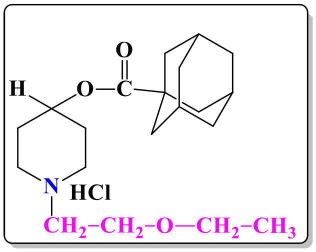

4d

Fig.S4, Fig.S21- Fig.S25

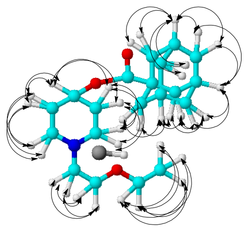

COSY

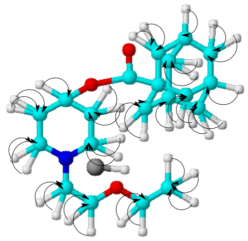

HMQC

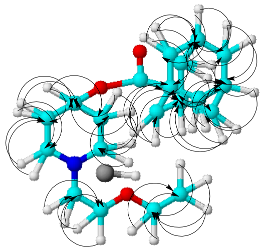

HMBC

1-(2-ethoxyethylpiperidin-4-yl)adamantane-1-carboxylate (hydrochloride) (4d).

White powder, yield 73 %, melting point 154-157°C.

IR spectrum (KBr), ν, см$^{-1}$: 1722.5 (C=O).

^{1}H NMR spectrum (DMSO-d$_6$), δ, ppm (J, Hz): 1.06-1.11 m (3H, $H^{11,11,11}$), 1.64 m (7H, $H^{18ax,20ax,22ax,18eq,20eq,22eq,3ax}$), 1.75-1.81 m (7H, $H^{16ax,23ax,24ax,16eq,23eq,24eq,,5ax}$), 1.93-2.21 m (5H, $H^{17,19,21,3eq,5eq}$), 3.00-3.27 m (4H, $H^{2ax,6ax,2eq,6eq}$), 3.41-3.44 m (4H, $H^{7,7,10,10}$), 3.76 m (2H, $H^{8,8}$), 4.72-4.89 m (1H, H^4), 11.27-11.33 m (1H, H^{22}).

^{13}C NMR spectrum (DMSO-d$_6$), δC, ppm: 15.45 (C^{11}), 26.98 ($C^{3,5}$), 27.84 ($C^{17,19,21}$), 36.55 ($C^{18,20,22}$), 38.70 ($C^{16,23,24,15}$), 48.01 and 50.57 ($C^{2,6}$), 55.89 and 55.36 (C^7), 64.01 (C^4), 64.75 (C^8), 66.20 (C^{10}), 176.03 (C^{13}).

COSY NMR spectrum: $H^{18ax,20ax,22ax}\rightarrow H^{17,19,21}$, $H^{3ax,5ax}\rightarrow H^{3eq,5eq}$, $H^{11}\rightarrow H^{10}$, $H^7\rightarrow H^8$.

HMQC NMR spectrum: $H^{11}\rightarrow C^{11}$, $H^{18,20,22}\rightarrow C^{18,20,22}$, $H^{16ax,23ax,24ax}\rightarrow C^{16,23,24}$, $H^{16eq,23eq,24eq}\rightarrow C^{16,23,24}$, $H^{17,19,21}\rightarrow C^{17,19,21}$, $H^{3ax,5ax}\rightarrow C^{3,5}$, $H^{3eq,5eq}\rightarrow C^{3,5}$, $H^{2ax,6ax}\rightarrow C^{2,6}$, $H^{2eq,6eq}\rightarrow C^{2,6}$, $H^7\rightarrow C^7$, $H^8\rightarrow C^8$, $H^4\rightarrow C^4$, $H^{10}\rightarrow C^{10}$.

HMBC NMR spectrum: $H^{11}\rightarrow C^{10}$; $H^{16ax,23ax,24ax}\rightarrow C^{17,19,21}$; $H^{10}\rightarrow C^{11}$, C^8; $H^8\rightarrow C^{10}$; $H^4\rightarrow C^{2,6}$.

Found, %: Carbon (C) 64,60; Hydrogen (H) 9,21; Nitrogen (N) 3,77; $C_{20}H_{34}ClNO_3$.

Calculated, %: Carbon (C) 64,57; Hydrogen (H) 9,28; Nitrogen (N) 3,72.

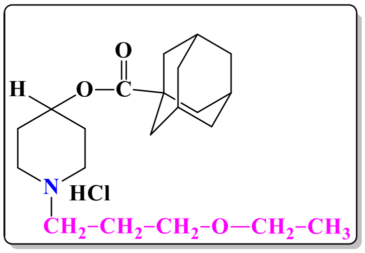

4e

Fig.S5, Fig.S26 – Fig.S30

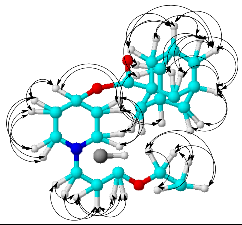

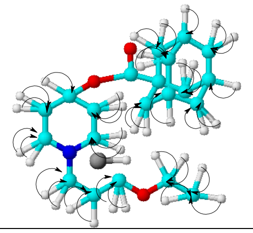

1-(3-ethoxypropylpiperidin-4-yl)adamantane-1-carboxylate (hydrochloride) (4e).

White powder, yield 68 %, melting point 164-167°C.

IR spectrum (KBr), ν, см$^{-1}$: 1721.9 (C=O).

^{1}H NMR spectrum (DMSO-d$_6$), δ, ppm (J, Hz): 1.05-1.09 m (3H, $H^{12,12,12}$), 1.64 m (7H, $H^{19ax,21ax,23x,19eq,21eq,23eq,3ax}$), 1.76-1.81 m (7H, $H^{17ax,24ax,25ax,17eq,24eq,25eq,,5ax}$), 1.94-2.19 m (7H, $H^{18,20,22,3eq,5eq,8,8}$), 2.89-3.08 m (4H, $H^{2ax,6ax,7,7}$), 3.33-3.42 m (6H, $H^{9,9,11,11,2eq,6eq}$), 4.76-4.91 m (1H, H^4), 11.01-11.16 m (1H, H^{26}).

^{13}C NMR spectrum (DMSO-d$_6$), δC, ppm: 15.60 (C^{12}), 24.35 (C^8), 26.98 ($C^{3,5}$), 27.84 ($C^{18,20,22}$), 36.40 ($C^{19,21,23}$), 38.67 ($C^{17,24,25,16}$), 47.65 and 49.99 ($C^{2,6}$), 53.57 and 54.25 (C^7), 64.08 (C^4), 66.04 (C^{11}), 67.34 and 67.77 (C^9), 176.11 and 176.35 (C^{14}).

COSY NMR spectrum: $H^{19ax,21ax,23ax}\rightarrow H^{18,20,22}$, $H^{17ax,24ax,25ax}\rightarrow H^{18,20,22}$, $H^{3ax,5ax}\rightarrow H^{3eq,5eq}$, $H^{2ax,6ax}\rightarrow H^{2eq,6eq}$, $H^8\rightarrow H^7$, $H^{12}\rightarrow H^{11}$, $H^9\rightarrow H^{11}$.

HMQC NMR spectrum: $H^{12}\rightarrow C^{12}$, $H^{9,11}\rightarrow C^{9,11}$, $H^8\rightarrow C^8$, $H^7\rightarrow C^7$, $H^{18,20,22}\rightarrow C^{18,20,22}$, $H^{17ax,24ax,25ax}\rightarrow C^{17,24,25}$, $H^{17eq,24eq,25eq}\rightarrow C^{17,24,25}$, $H^{3ax,5ax}\rightarrow C^{3,5}$, $H^{3eq,5eq}\rightarrow C^{3,5}$, $H^{2ax,6ax}\rightarrow C^{2,6}$, $H^{2eq,6eq}\rightarrow C^{2,6}$, $H^4\rightarrow C^4$.

COSY HMQC<br>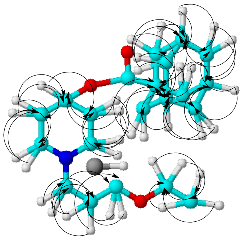<br>HMBC	HMBC NMR spectrum: $H^{12}\rightarrow C^{11}$; $H^{17ax,24ax,25ax}\rightarrow C^{19,21,23}$, $C^{18,20,22}$; $H^{19ax,21ax,23ax}\rightarrow C^{18,20,22}$; $H^{8}\rightarrow C^{7}$, C^{9}; $H^{11}\rightarrow C^{12}$, C^{8}, C^{7}.<br><br>Found, %: Carbon (C) 65,35; Hydrogen (H) 9,41; Nitrogen (N) 3,63; $C_{21}H_{36}ClNO_3$.<br>Calculated, %: Carbon (C) 65,72; Hydrogen (H) 9,53; Nitrogen (N) 3,61.
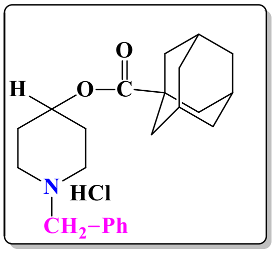<br>**4f**<br><br>**Fig.S6, Fig.S31 – Fig.S35**<br><br>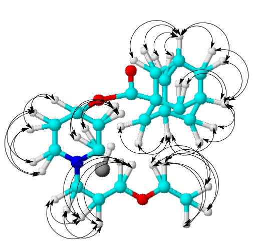 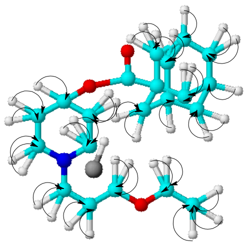<br>COSY HMQC<br><br>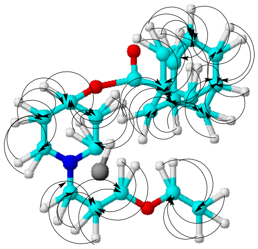<br>HMBC	**(1-benzylpiperidin-4-yl)adamantane-1-carboxylate (hydrochloride) (4f).**<br>White powder, yield 45 %, melting point 203-205°C.<br>IR spectrum (KBr), ν, см$^{-1}$: 1722.2 (C=O).<br><br>^{1}H NMR spectrum (DMSO-d_6), δ, ppm (J, Hz): 1.58–1.66 m (6H, $H^{20ax,\ 22ax,\ 24ax,\ 20eq,\ 22eq,\ 24eq}$), 1.72–1.74 m (6H, $H^{18ax,\ 25ax,\ 26ax,\ 18eq,\ 25eq,\ 26eq}$), 1.81–1.99 m (5H, $H^{19,\ 21,\ 23,\ 3ax,\ 5ax}$), 2.09–2.16 m (2H, $H^{3eq,\ 5eq}$), 2.87–3.06 m (2H, $H^{2ax,\ 6ax}$), 3.20–3.28 m (2H, $H^{2eq,\ 6eq}$), 4.21–4.31 m (2H, $H^{7,7}$), 4.72–4.88 m (1H, H^{4}), 7.41–7.42 m (3H, $H^{9,11,13}$), 7.56–7.62 m (2H, $H^{10,12}$), 11.13–11.24 m (1H, H^{27}).<br>^{13}C NMR spectrum (DMSO-d_6), δC, ppm: 26.81 ($C^{3,5}$), 27.77 ($C^{19,21,23}$), 36.53 ($C^{20,22,24}$), 38.68 ($C^{18,25,26}$), 38.78 (C^{17}), 46.76 ($C^{2,6}$), 58.62 and 58.97 (C^{7}), 64.08 (C^{4}), 129.29 ($C^{9,13}$), 129.94 (C^{11}), 131.91 (C^{8}), 132.28 ($C^{10,12}$), 175.91 (C^{15}).<br>COSY NMR spectrum: $H^{20ax,22ax,24ax}\rightarrow H^{19,21,23}$, $H^{20eq,22eq,24eq}\rightarrow H^{19,21,23}$, $H^{3ax,5ax}\rightarrow H^{3eq,5eq}$, $H^{3eq,5eq}\rightarrow H^{2ax,6ax}$, $H^{2ax,6ax}\rightarrow H^{2eq,6eq}$, $H^{9,13}\rightarrow H^{10,12}$.<br>HMQC NMR spectrum: $H^{20,22,24}\rightarrow C^{20,22,24}$, $H^{18,25,26}\rightarrow C^{18,25,26}$, $H^{19,21,23}\rightarrow C^{19,21,23}$, $H^{3ax,5ax}\rightarrow C^{3,5}$, $H^{3eq,5eq}\rightarrow C^{3,5}$, $H^{2ax,6ax}\rightarrow C^{2,6}$, $H^{2eq,6eq}\rightarrow C^{2,6}$, $H^{7}\rightarrow C^{7}$, $H^{4}\rightarrow C^{4}$, $H^{10,12}\rightarrow C^{10,12}$, $H^{11}\rightarrow C^{11}$, $H^{9,13}\rightarrow C^{9,13}$.<br>HMBC NMR spectrum: $H^{20ax,22ax,24ax}\rightarrow C^{19,21,23}$, $C^{18,25,26}$; $H^{18,25,26}\rightarrow C^{19,21,23}$, $C^{20,22,24}$; $H^{7}\rightarrow C^{2,6}$, $C^{9,13}$; $H^{9,13}\rightarrow C^{10,12}$; $H^{10,12}\rightarrow C^{7}$, $C^{9,13}$.<br><br>Found, %: Carbon (C) 70,83; Hydrogen (H) 8,27; Nitrogen (N) 3,59; $C_{23}H_{32}ClNO_2$.<br>Calculated, %: Carbon (C) 70,61; Hydrogen (H) 8,32; Nitrogen (N) 3,64.

Copies of IR Spectra of Products

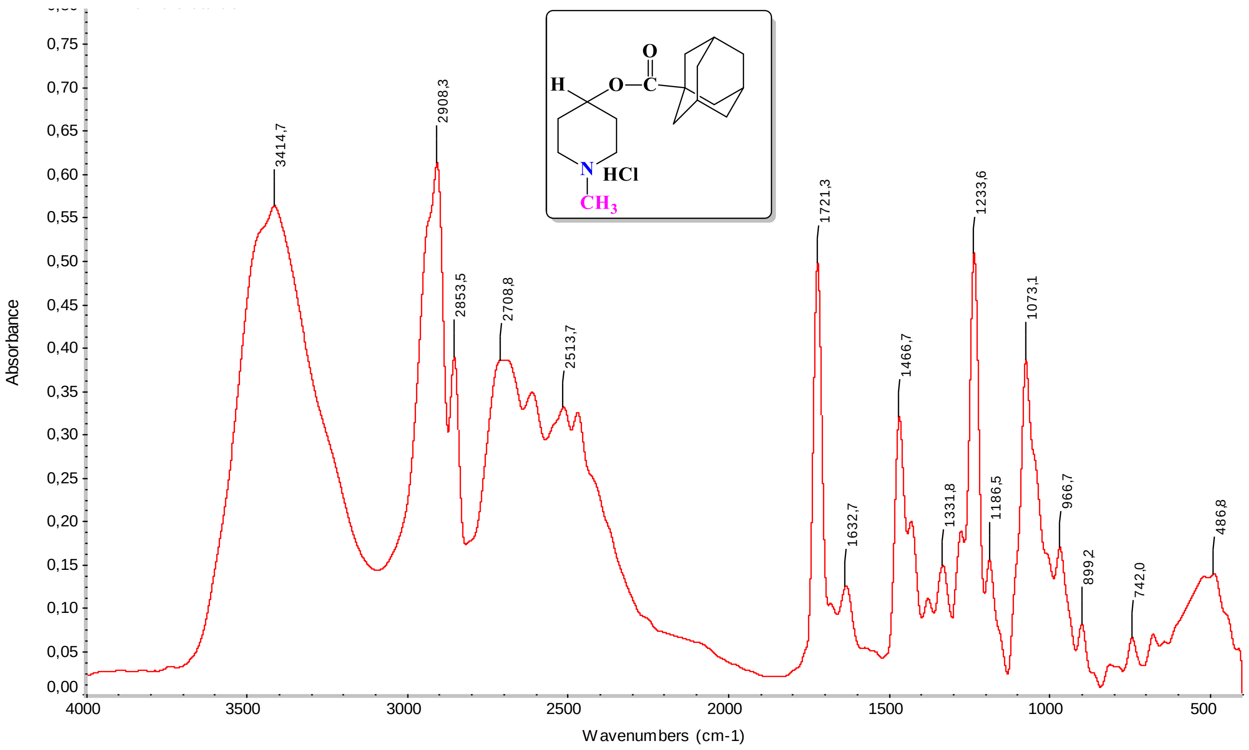

Fig. S1. IR spectra of compound **4a**

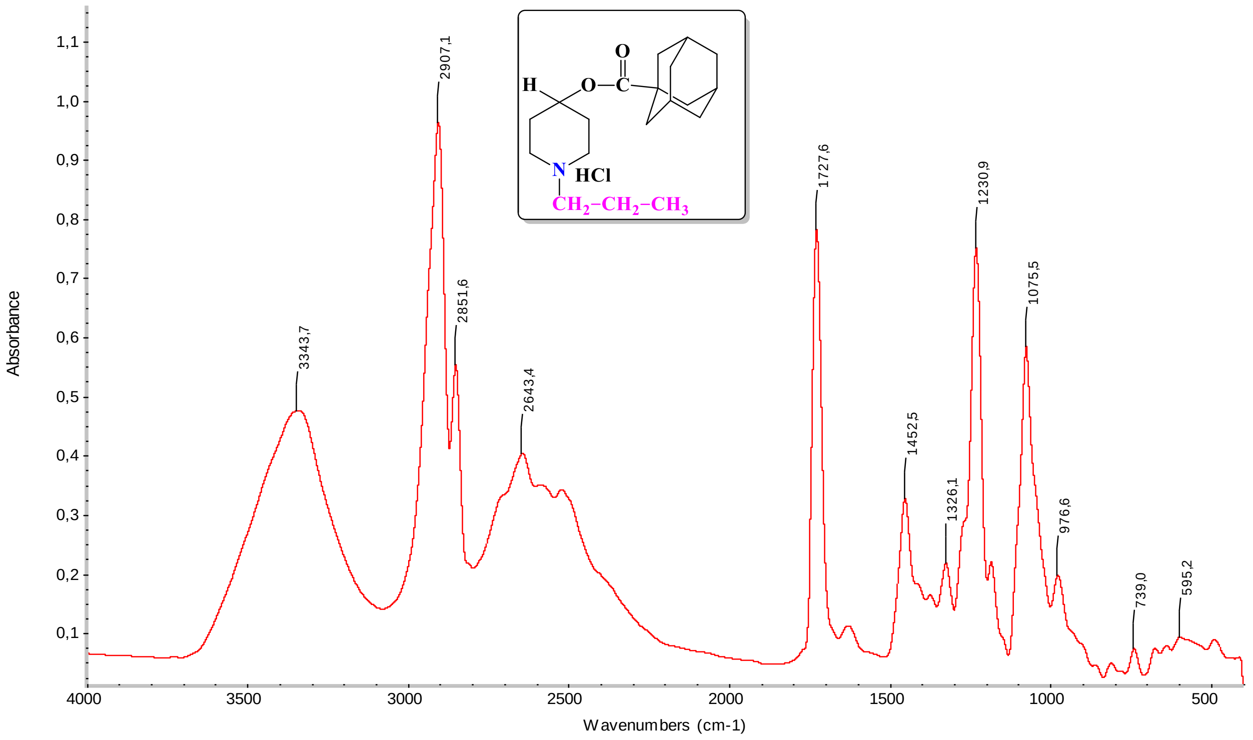

Fig. S2. IR spectra of compound **4b**

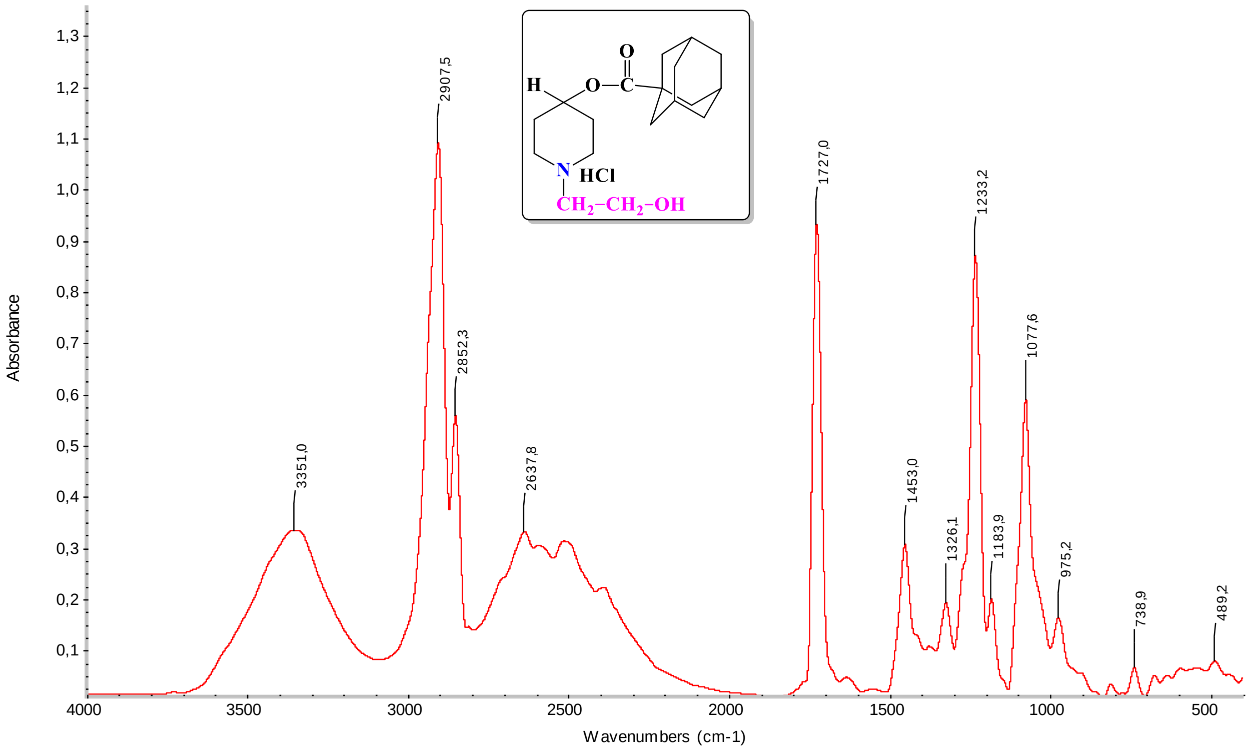

Fig. S3. IR spectra of compound **4c**

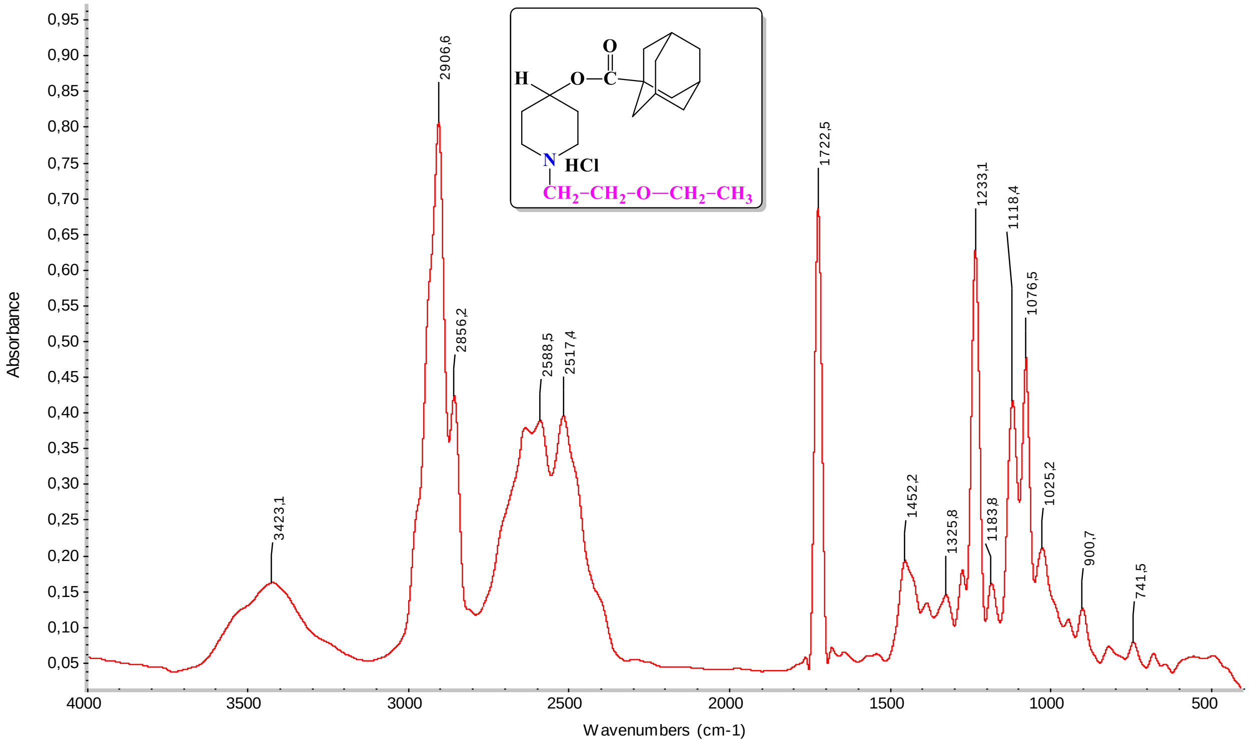

Fig. S4. IR spectra of compound **4d**

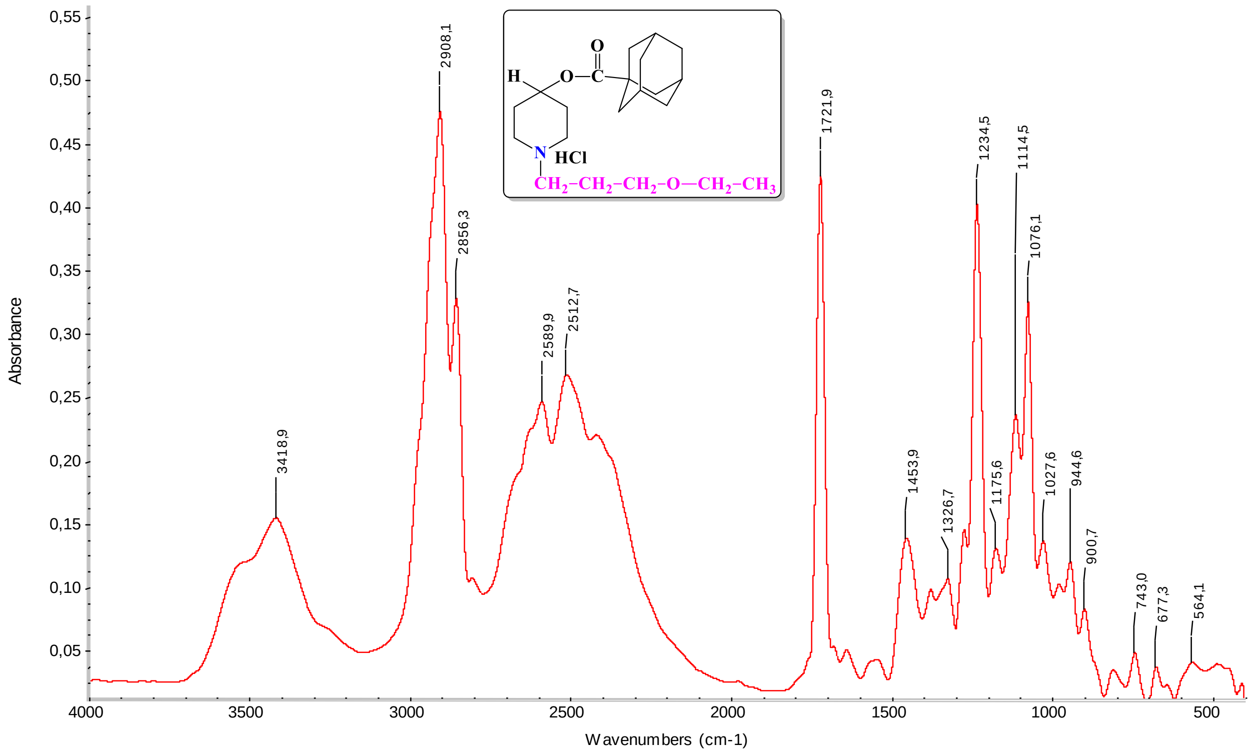

Fig. S5. IR spectra of compound **4e**

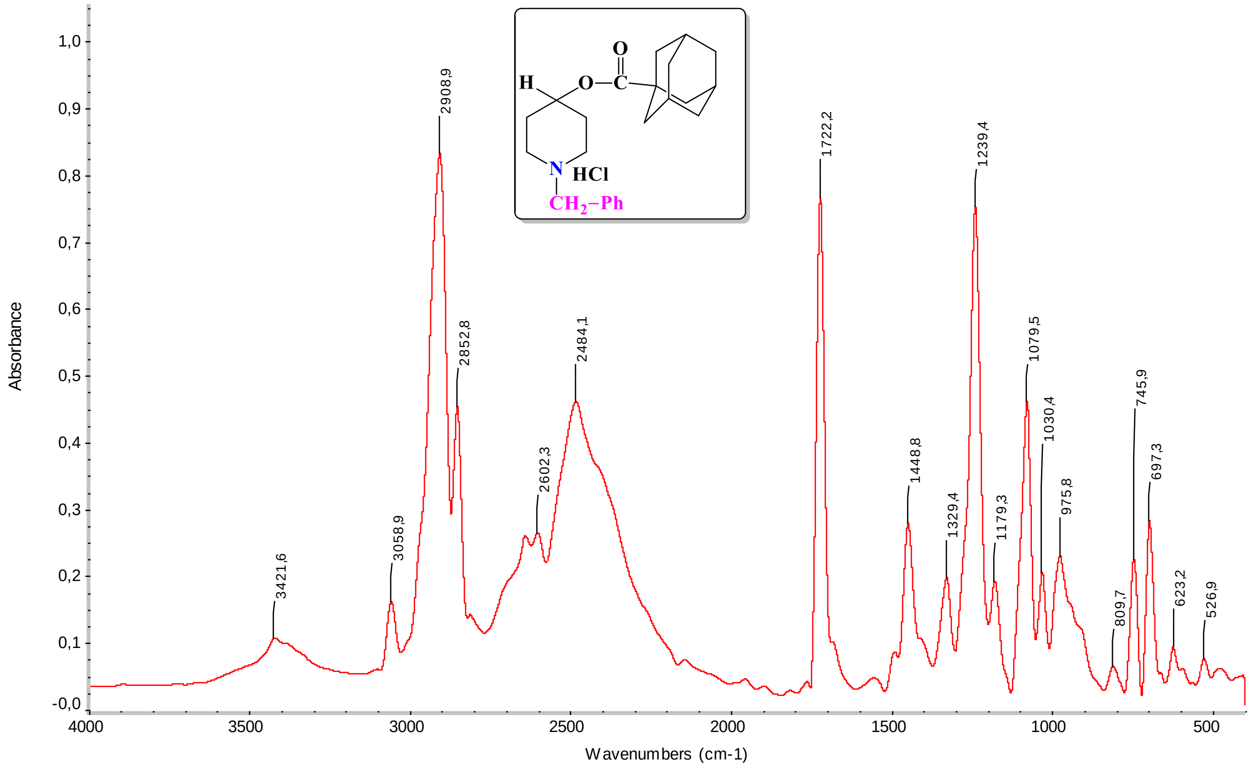

Fig. S6. IR spectra of compound **4f**

Copies of NMR Spectra of Products

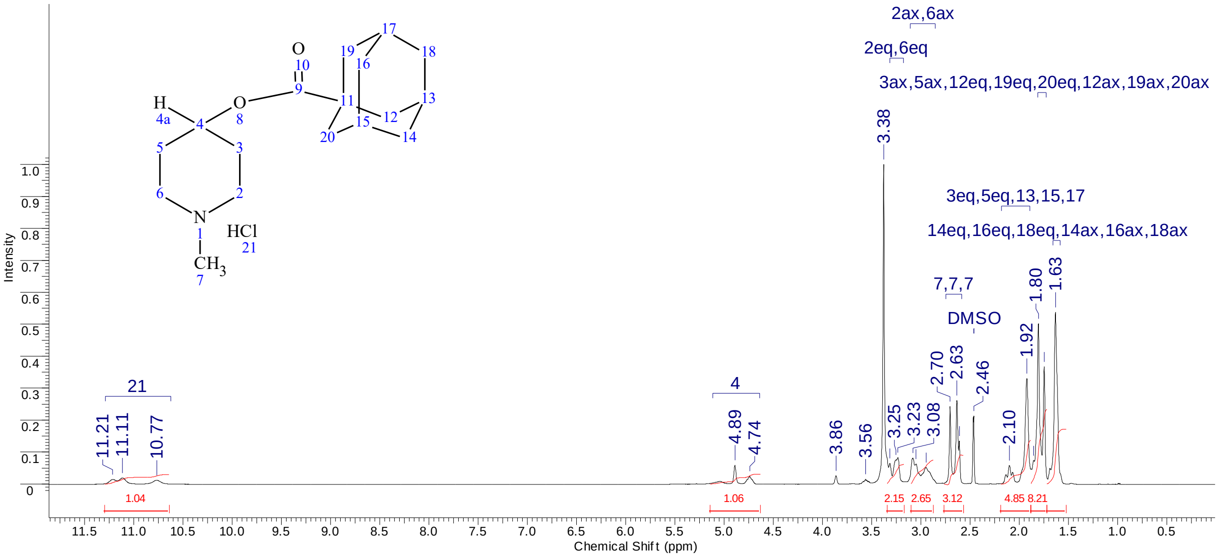

No.	(ppm)	(Hz)	Height
1	1.63	652.1	0.5371
2	1.75	697.9	0.3671
3	1.80	721.2	0.5014
4	1.85	740.5	0.0738
5	1.92	768.4	0.3301
6	2.10	838.9	0.0582
7	2.46	984.1	0.2131
8	2.61	1042.7	0.1353
9	2.63	1052.8	0.2610
10	2.70	1080.3	0.2421
11	2.95	1177.8	0.0515
12	3.05	1218.1	0.0625

No.	(ppm)	(Hz)	Height
13	3.08	1231.4	0.0814
14	3.23	1292.8	0.0833
15	3.25	1300.1	0.0762
16	3.31	1324.4	0.0645
17	3.38	1350.0	1.0000
18	3.56	1422.8	0.0156
19	3.86	1543.7	0.0257
20	4.74	1894.0	0.0246
21	4.89	1954.5	0.0587
22	10.77	4303.8	0.0119
23	11.11	4443.4	0.0190
24	11.21	4482.8	0.0145

No.	Annotation	(ppm)
1	14eq,16eq,18eq,14ax,16ax,18ax	[1.58 .. 1.66]
2	3ax,5ax,12eq,19eq,20eq,12ax,19ax,20ax	[1.73 .. 1.81]
3	3eq,5eq,13,15,17	[1.89 .. 2.18]
4	DMSO	2.46
5	7,7,7	[2.58 .. 2.75]
6	2ax,6ax	[2.85 .. 3.11]
7	2eq,6eq	[3.18 .. 3.31]
8	4	[4.64 .. 5.12]
9	21	[10.62 .. 11.29]

No.	(ppm)	Value	Absolute Value
1	[10.64 .. 11.30]	1.035	8.11719e+0
2	[4.63 .. 5.14]	1.063	8.33918e+0
3	[3.17 .. 3.34]	2.151	1.68652e+1
4	[2.87 .. 3.10]	2.645	2.07420e+1
5	[2.57 .. 2.76]	3.119	2.44582e+1
6	[1.88 .. 2.19]	4.849	3.80246e+1
7	[1.52 .. 1.72]	6.000	4.70492e+1
8	[1.72 .. 1.88]	8.214	6.44088e+1

Fig. S7. ^{1}H NMR spectra of compound **4a** (in DMSO)

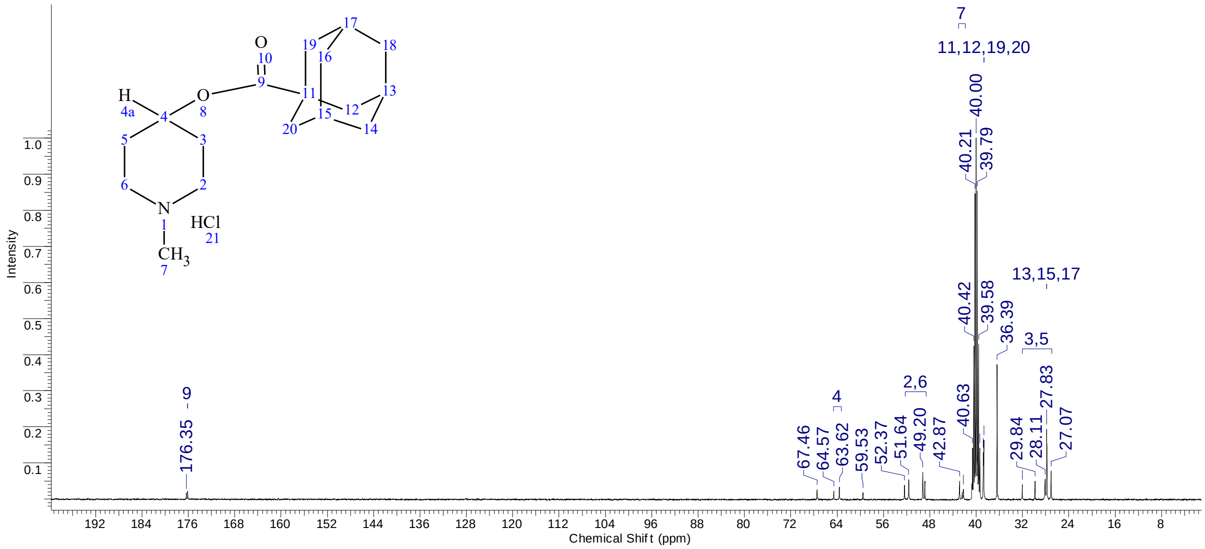

No.	(ppm)	(Hz)	Height
1	27.07	2721.2	0.0779
2	27.83	2797.9	0.1935
3	28.11	2825.7	0.0552
4	29.84	3000.1	0.0491
5	32.04	3220.6	0.0408
6	36.39	3657.6	0.3730
7	38.68	3888.6	0.1631
8	38.76	3896.3	0.1290
9	39.37	3957.6	0.1413
10	39.58	3978.7	0.4285
11	39.79	3999.8	0.8528
12	40.00	4020.9	1.0000
13	40.21	4042.0	0.8463

No.	(ppm)	(Hz)	Height
14	40.42	4063.1	0.4240
15	40.63	4084.2	0.1418
16	42.20	4242.3	0.0264
17	42.87	4309.4	0.0495
18	49.20	4945.8	0.0748
19	51.64	5191.2	0.0533
20	52.37	5265.0	0.0379
21	59.53	5983.8	0.0176
22	63.62	6395.0	0.0330
23	64.57	6490.9	0.0218
24	67.46	6781.3	0.0254
25	176.35	17727.9	0.0141

No.	Annotation	(ppm)
1	3,5	[26.91 .. 32.04]
2	13,15,17	27.83
3	11,12,19,20	38.68
4	7	[41.85 .. 43.05]
5	2,6	[48.64 .. 52.26]
6	4	[63.27 .. 64.64]
7	9	176.11

Fig. S8. ^{13}C NMR spectra of compound **4a** (in DMSO)

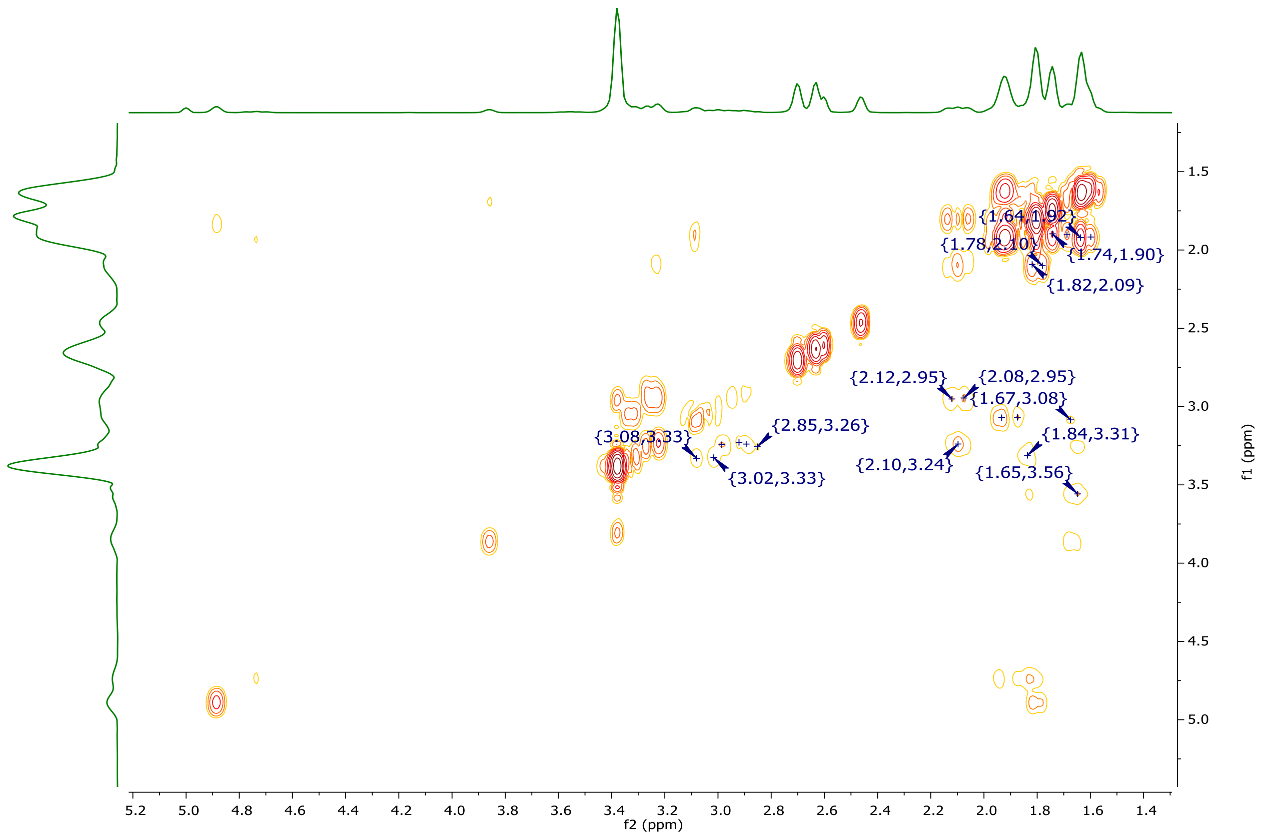

Fig. S9. COSY of compound **4a** (in DMSO)

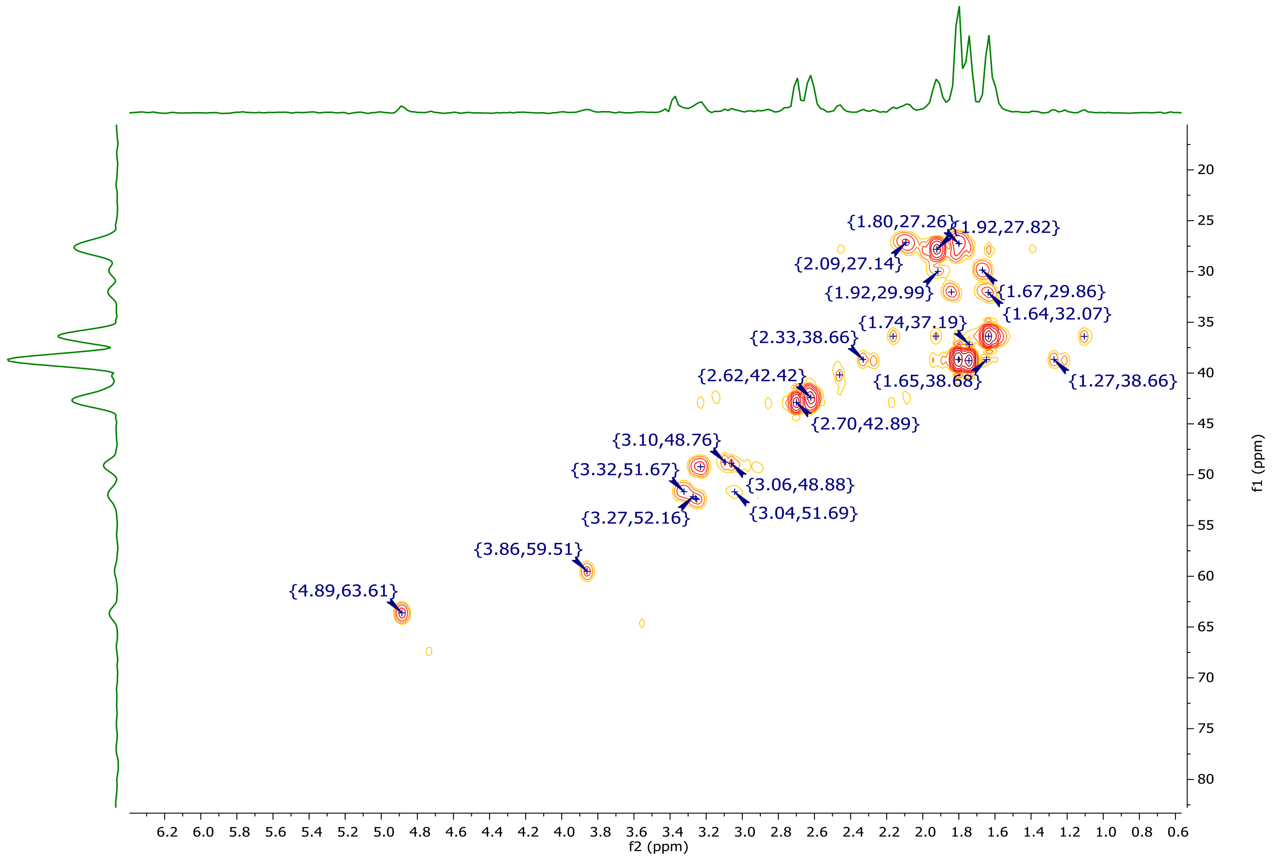

Fig. S10. HMQC of compound **4a** (in DMSO)

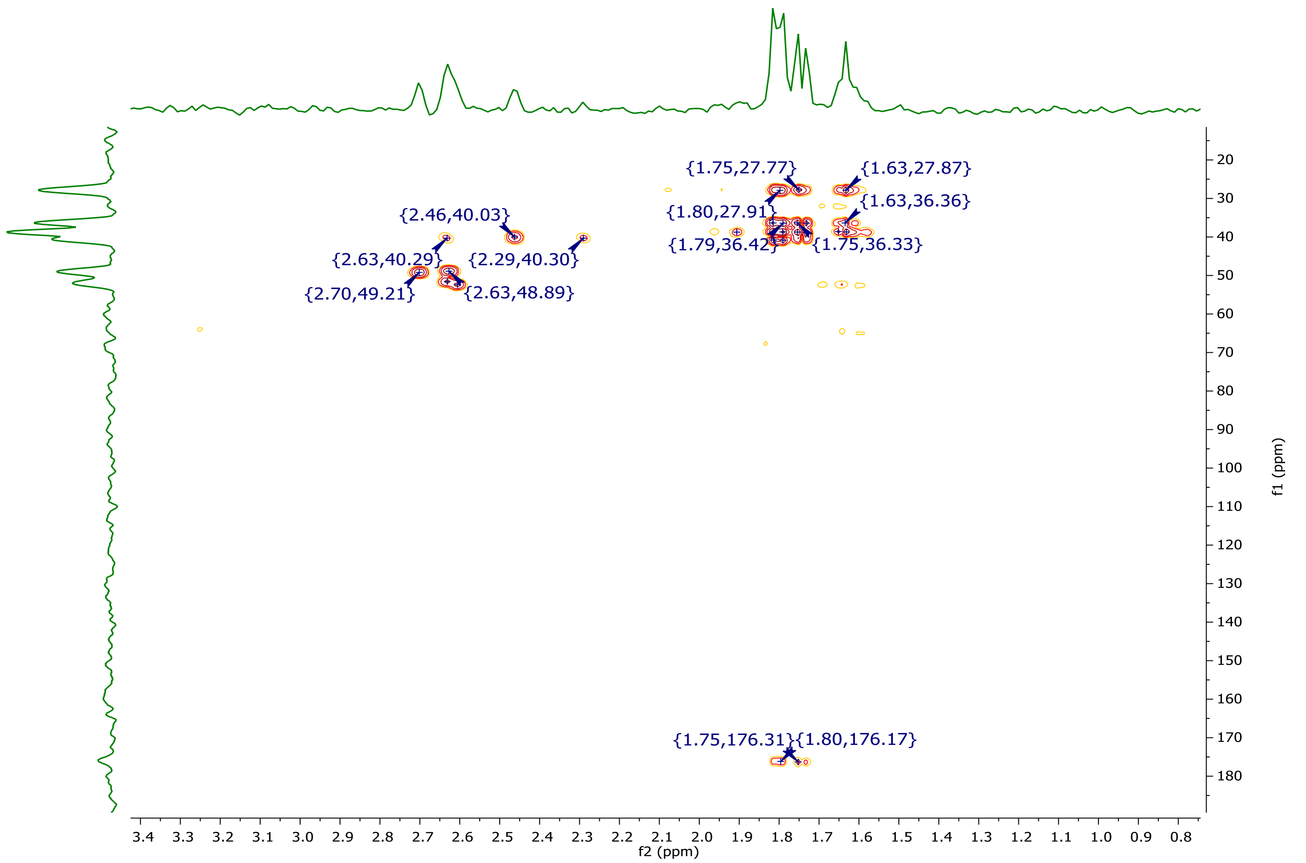

Fig. S11. HMBC of compound **4a** (in DMSO)

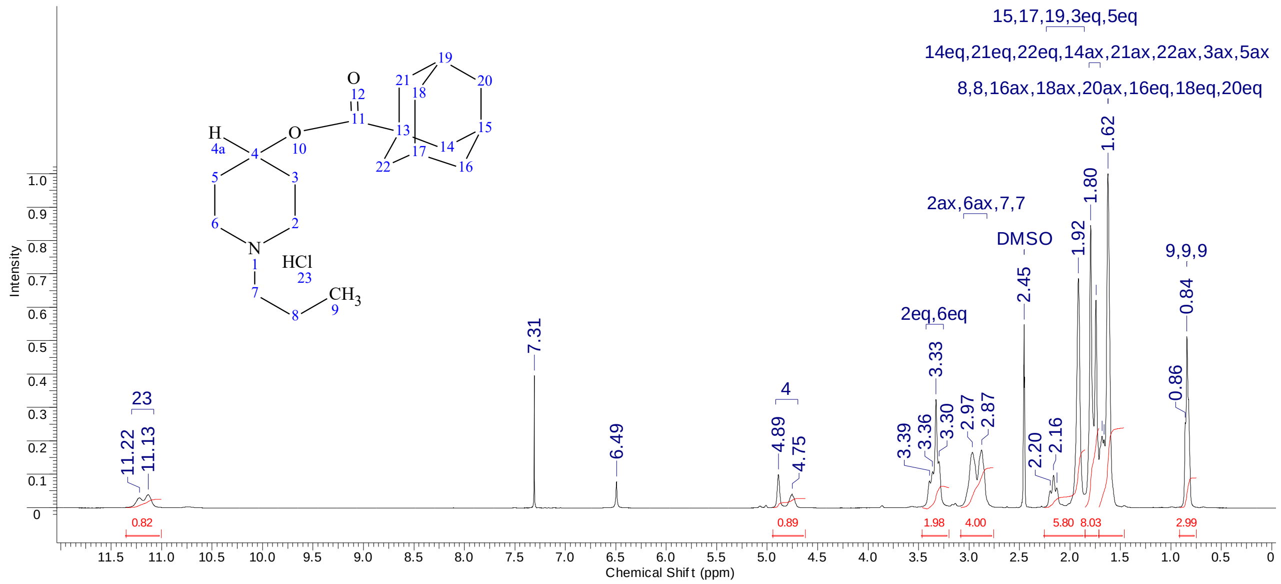

No.	(ppm)	(Hz)	Height
1	0.84	336.3	0.5125
2	0.86	343.0	0.2539
3	1.62	649.4	1.0000
4	1.66	664.7	0.2030
5	1.68	672.6	0.2144
6	1.74	696.4	0.6207
7	1.80	717.8	0.8448
8	1.92	766.0	0.6863
9	2.13	851.5	0.0586
10	2.16	864.3	0.0962
11	2.20	877.7	0.0504
12	2.45	980.8	0.5477

No.	(ppm)	(Hz)	Height
13	2.87	1149.3	0.1728
14	2.97	1185.9	0.1656
15	3.30	1318.4	0.1386
16	3.33	1330.0	0.3242
17	3.36	1344.0	0.1075
18	3.39	1356.2	0.0797
19	4.75	1900.0	0.0406
20	4.89	1954.4	0.0996
21	6.49	2595.8	0.0783
22	7.31	2921.2	0.3946
23	11.13	4451.3	0.0385
24	11.22	4485.5	0.0290

No.	Annotation	(ppm)
1	9,9,9	0.84
2	8,8,16ax,18ax,20ax,16eq,18eq,20eq	1.62
3	14eq,21eq,22eq,14ax,21ax,22ax,3ax,5ax	[1.70 .. 1.81]
4	15,17,19,3eq,5eq	[1.86 .. 2.23]
5	DMSO	2.45
6	2ax,6ax,7,7	[2.82 .. 3.05]
7	2eq,6eq	[3.25 .. 3.43]
8	4	[4.69 .. 4.92]
9	23	[11.08 .. 11.30]

No.	(ppm)	Value	Absolute Value
1	[11.00 .. 11.35]	0.823	7.30415e+0
2	[4.62 .. 4.94]	0.895	7.93429e+0
3	[3.20 .. 3.46]	1.985	1.76039e+1
4	[0.75 .. 0.91]	2.986	2.64821e+1
5	[2.76 .. 3.08]	4.000	3.54793e+1
6	[1.85 .. 2.25]	5.801	5.14520e+1
7	[1.46 .. 1.71]	8.005	7.10071e+1
8	[1.71 .. 1.85]	8.026	7.11887e+1

Fig. S12. ^{1}H NMR spectra of compound **4b** (in DMSO)

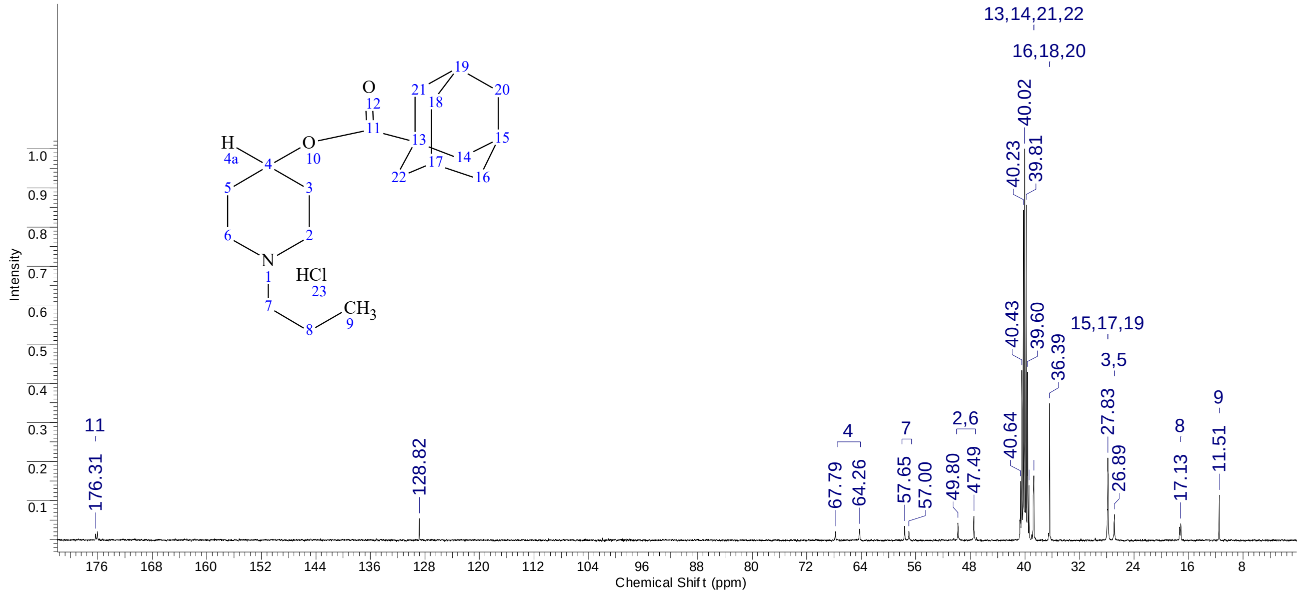

No.	(ppm)	(Hz)	Height
1	11.51	1157.0	0.1140
2	17.13	1721.5	0.0401
3	26.89	2703.0	0.0641
4	27.83	2797.9	0.2087
5	36.39	3657.6	0.3487
6	38.69	3889.6	0.1637
7	39.39	3959.6	0.1385
8	39.60	3980.6	0.4291
9	39.81	4001.7	0.8565
10	40.02	4022.8	1.0000
11	40.23	4043.9	0.8433

No.	(ppm)	(Hz)	Height
12	40.43	4064.0	0.4327
13	40.64	4085.1	0.1492
14	47.49	4774.3	0.0601
15	49.80	5006.2	0.0422
16	57.00	5729.8	0.0210
17	57.65	5795.0	0.0342
18	64.26	6460.2	0.0274
19	67.79	6814.8	0.0211
20	128.82	12950.0	0.0541
21	176.31	17724.1	0.0147

No.	Annotation	(ppm)
1	9	11.51
2	8	17.13
3	3,5	26.89
4	15,17,19	27.83
5	16,18,20	36.39
6	13,14,21,22	38.69
7	2,6	[47.23 .. 49.99]
8	7	[56.62 .. 58.00]
9	4	[64.08 .. 67.53]
10	11	176.31

Fig. S13. ^{13}C NMR spectra of compound **4b** (in DMSO)

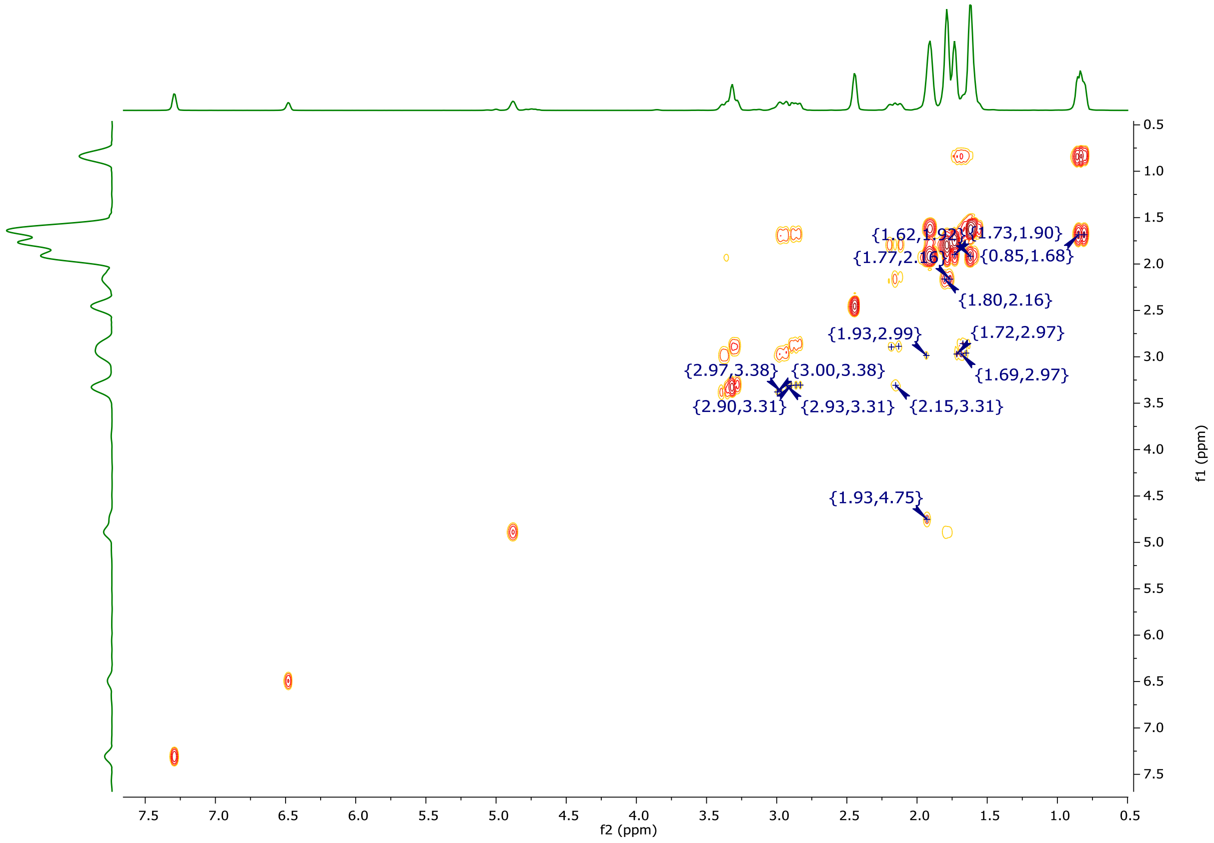

Fig. S14. COSY of compound **4b** (in DMSO)

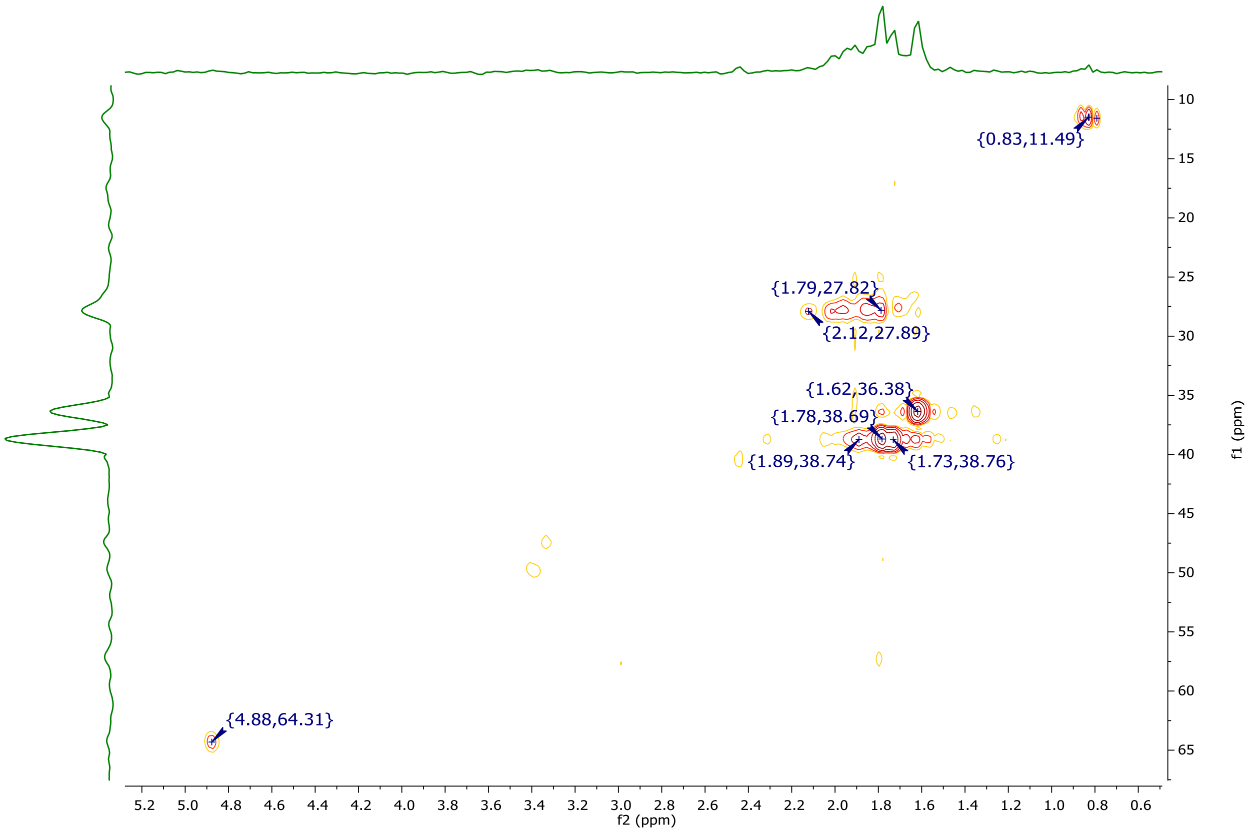

Fig. S15. HMQC of compound **4b** (in DMSO)

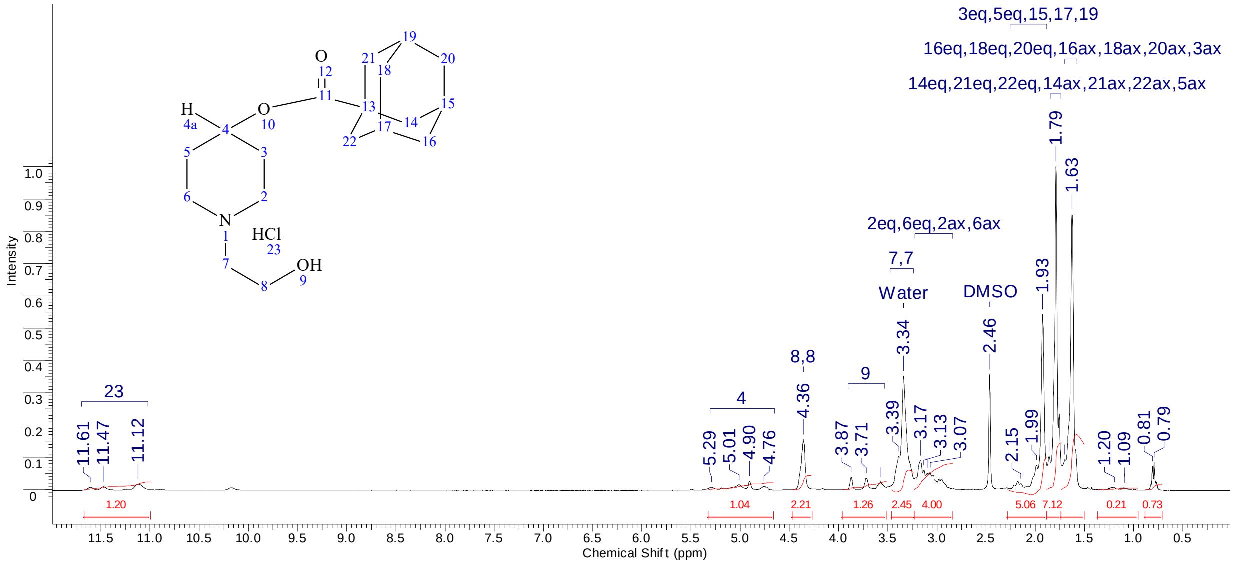

No.	(ppm)	(Hz)	Height
1	0.79	316.9	0.0839
2	0.81	323.3	0.0719
3	1.09	435.5	0.0059
4	1.20	478.5	0.0076
5	1.63	649.8	0.8527
6	1.70	679.6	0.0934
7	1.76	702.9	0.2364
8	1.79	715.3	1.0000
9	1.86	743.7	0.1024
10	1.93	769.8	0.5430

No.	(ppm)	(Hz)	Height
11	1.99	795.0	0.0750
12	2.15	857.7	0.0186
13	2.46	985.0	0.3563
14	3.07	1227.3	0.0529
15	3.10	1238.7	0.0512
16	3.13	1252.5	0.0607
17	3.17	1266.2	0.0896
18	3.34	1335.3	0.3517
19	3.39	1354.6	0.1030
20	3.58	1429.7	0.0241

No.	(ppm)	(Hz)	Height
21	3.71	1483.7	0.0349
22	3.87	1547.8	0.0393
23	4.36	1742.9	0.1556
24	4.76	1901.8	0.0109
25	4.90	1960.9	0.0259
26	5.01	2003.0	0.0150
27	5.29	2115.7	0.0075
28	11.12	4446.2	0.0166
29	11.47	4587.2	0.0092
30	11.61	4640.3	0.0069

No.	Annotation	(ppm)
1	16eq,18eq,20eq,16ax,18ax,20ax,3ax	[1.57 .. 1.70]
2	14eq,21eq,22eq,14ax,21ax,22ax,5ax	[1.74 .. 1.85]
3	3eq,5eq,15,17,19	[1.88 .. 2.26]
4	DMSO	2.46
5	2eq,6eq,2ax,6ax	[2.84 .. 3.22]
6	7,7	[3.24 .. 3.48]
7	Water	3.34
8	9	[3.53 .. 3.90]
9	8,8	4.36
10	4	[4.65 .. 5.31]
11	23	[11.02 .. 11.70]

No.	(ppm)	Value	Absolute Value
1	[0.96 .. 1.37]	0.211	1.43018e+0
2	[0.71 .. 0.88]	0.728	4.92696e+0
3	[4.66 .. 5.32]	1.043	7.05615e+0
4	[11.00 .. 11.67]	1.199	8.11084e+0
5	[3.51 .. 3.96]	1.258	8.51076e+0
6	[4.27 .. 4.47]	2.210	1.49450e+1
7	[3.23 .. 3.46]	2.448	1.65579e+1
8	[2.84 .. 3.23]	4.000	2.70529e+1
9	[1.89 .. 2.28]	5.061	3.42291e+1
10	[1.50 .. 1.74]	6.930	4.68661e+1
11	[1.74 .. 1.88]	7.116	4.81255e+1

Fig. S16. ^{1}H NMR spectra of compound **4c** (in DMSO)

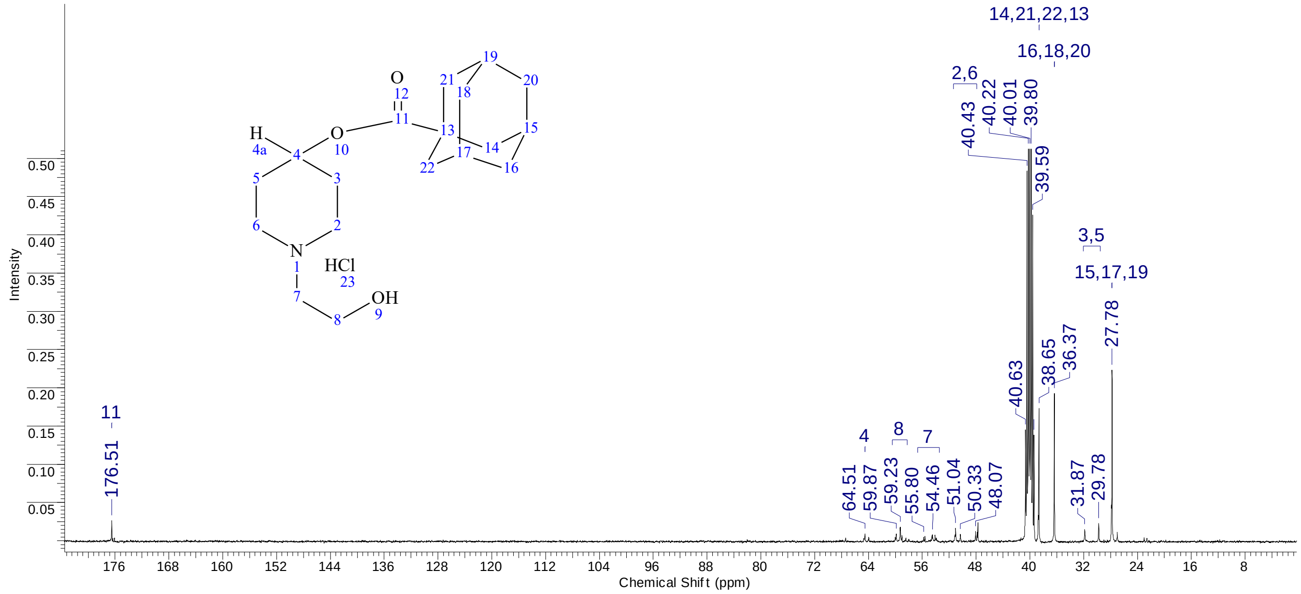

No.	(ppm)	(Hz)	Height
1	27.78	2792.1	0.2232
2	29.78	2993.4	0.0226
3	31.87	3203.3	0.0148
4	36.37	3655.7	0.1928
5	38.65	3885.8	0.1732
6	39.38	3958.6	0.1381
7	39.59	3979.7	0.4262
8	39.80	4000.8	0.8506
9	40.01	4021.9	1.0000
10	40.22	4042.9	0.8507
11	40.43	4064.0	0.4837

No.	(ppm)	(Hz)	Height
12	40.63	4084.2	0.1454
13	48.07	4831.8	0.0122
14	50.33	5059.9	0.0084
15	51.04	5130.8	0.0163
16	54.46	5474.9	0.0077
17	55.80	5609.1	0.0046
18	59.23	5954.1	0.0180
19	59.87	6018.3	0.0089
20	64.51	6485.1	0.0088
21	176.51	17743.3	0.0262

No.	Annotation	(ppm)
1	15,17,19	27.78
2	3,5	[29.54 .. 32.06]
3	16,18,20	36.37
4	14,21,22,13	38.65
5	2,6	[47.86 .. 51.36]
6	7	[53.46 .. 56.67]
7	8	[58.21 .. 60.45]
8	4	64.51
9	11	176.51

Fig. S17. ^{13}C NMR spectra of compound 4**c** (in DMSO)

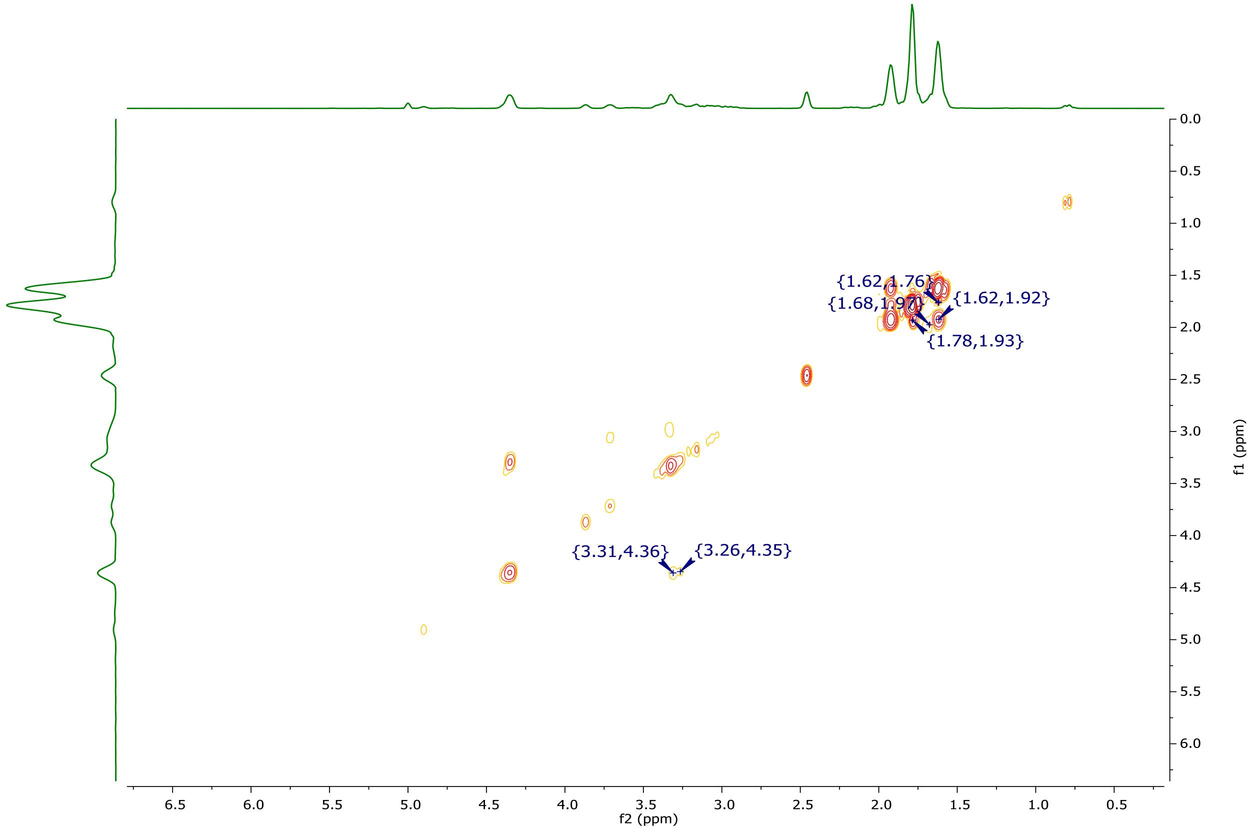

Fig. S18. COSY spectra of compound **4c** (in DMSO)

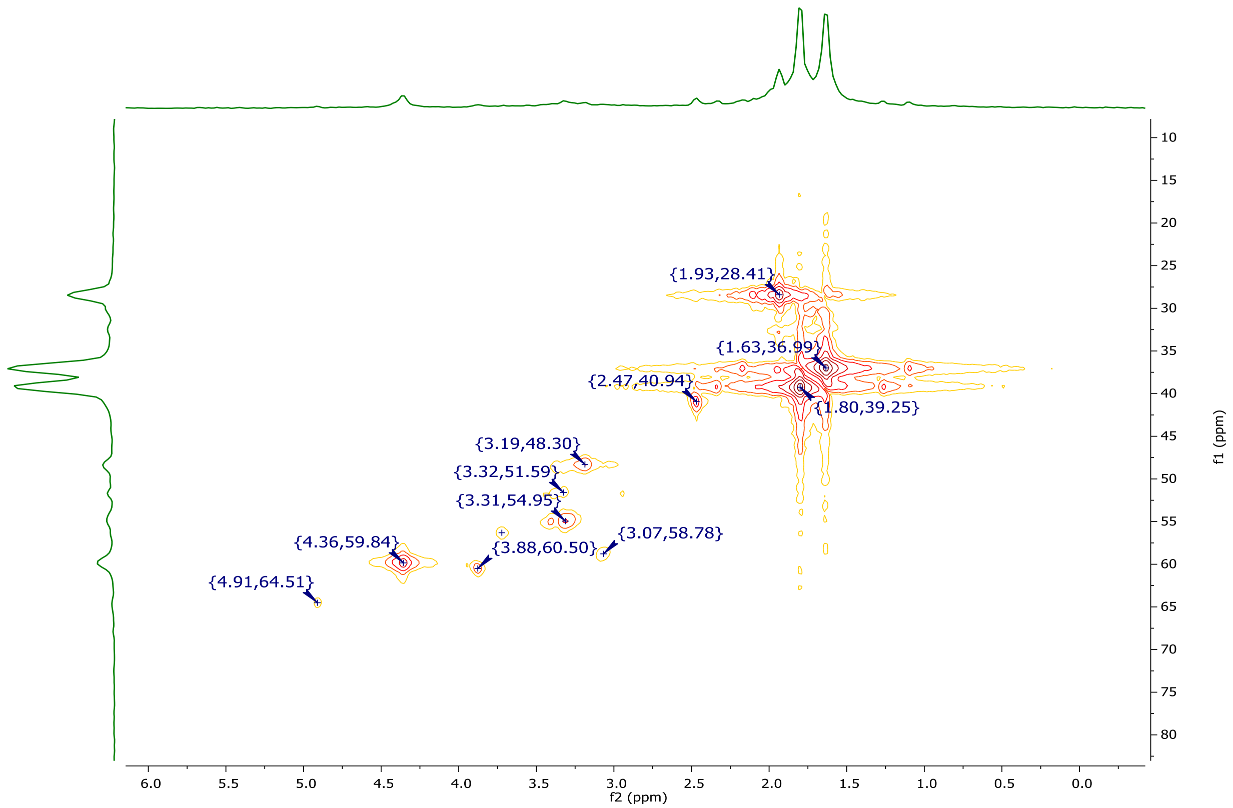

Fig. S19. HMQC of compound **4c** (in DMSO)

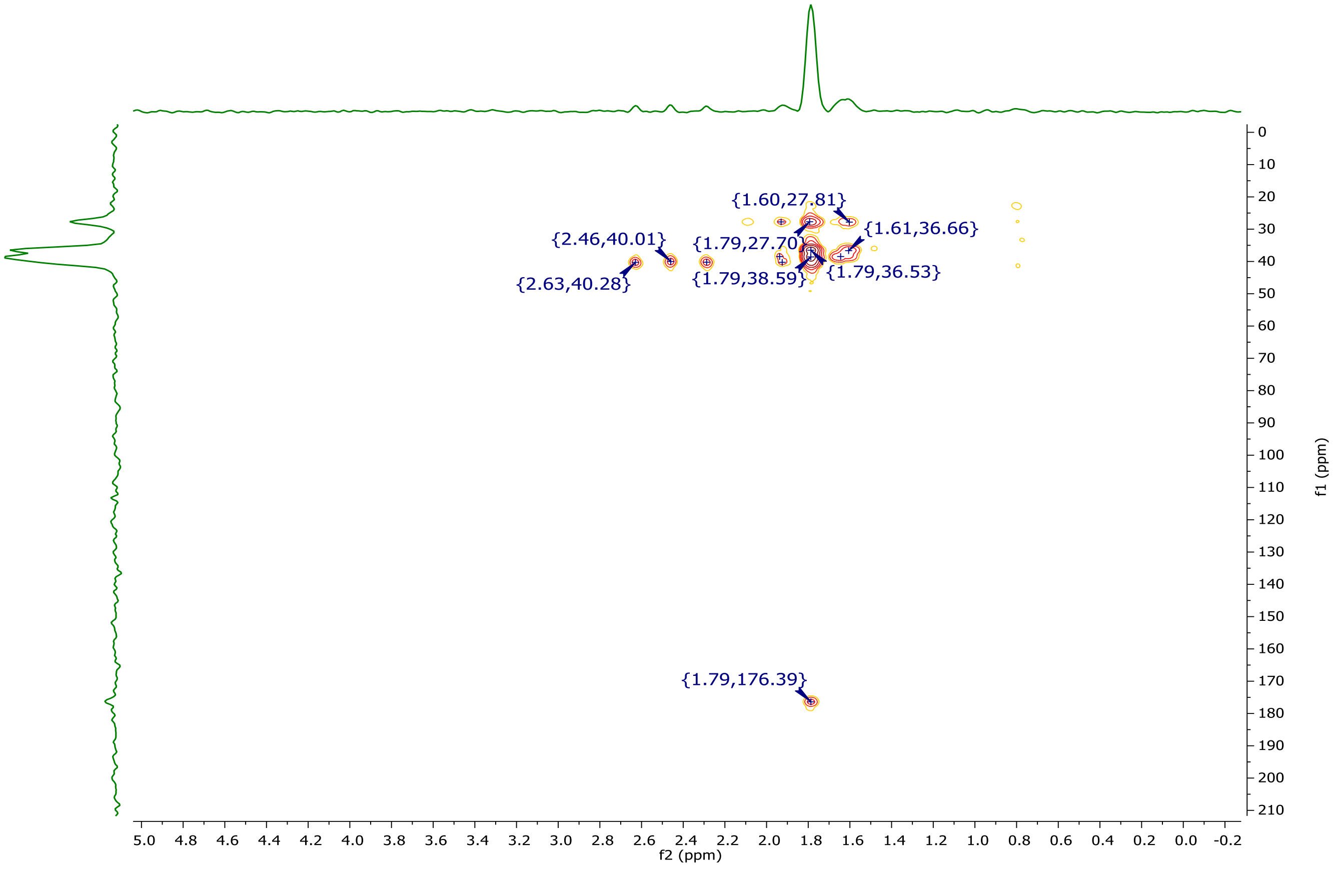

Fig. S20. HMBC of compound **4c** (in DMSO)

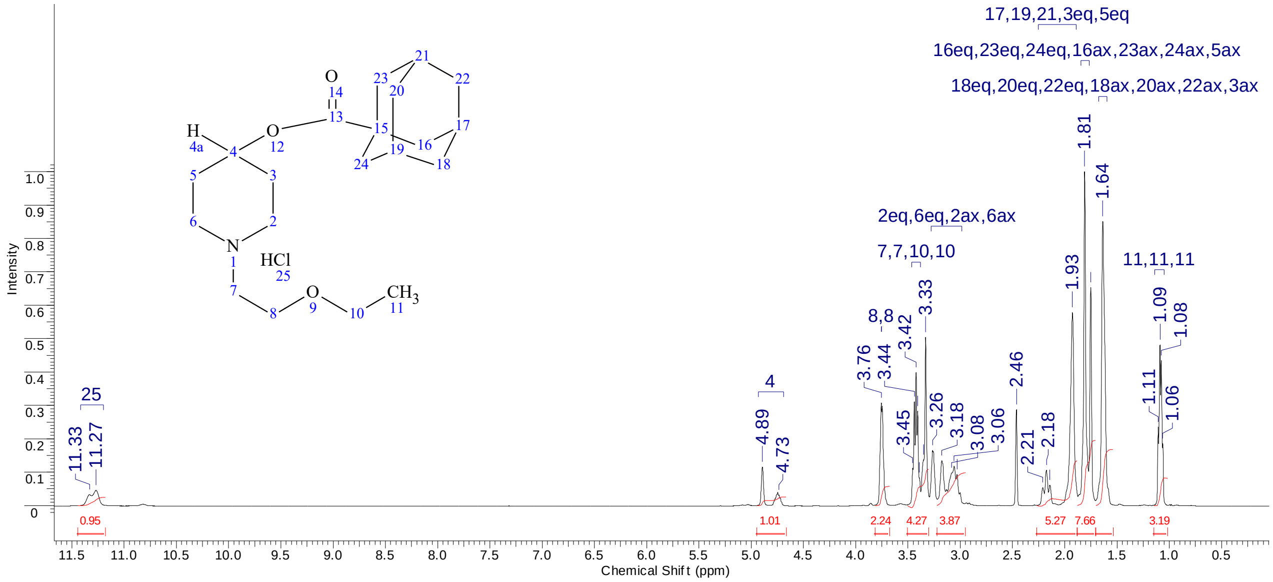

No.	(ppm)	(Hz)	Height
1	1.06	424.9	0.1850
2	1.08	430.4	0.4341
3	1.09	435.0	0.4821
4	1.11	441.9	0.2354
5	1.64	654.4	0.8505
6	1.75	700.2	0.6534
7	1.81	723.5	1.0000
8	1.93	770.2	0.5782
9	2.14	856.3	0.0609
10	2.18	869.6	0.1059
11	2.21	883.4	0.0536

No.	(ppm)	(Hz)	Height
12	2.46	984.1	0.2875
13	3.03	1211.7	0.0931
14	3.06	1222.7	0.1182
15	3.08	1232.3	0.0983
16	3.18	1269.9	0.1344
17	3.26	1302.4	0.1618
18	3.26	1305.1	0.1649
19	3.33	1331.7	0.5037
20	3.35	1339.9	0.1372
21	3.39	1356.0	0.0838
22	3.41	1361.9	0.2823

No.	(ppm)	(Hz)	Height
23	3.42	1368.3	0.3992
24	3.44	1374.7	0.3116
25	3.45	1381.1	0.1125
26	3.75	1497.5	0.2979
27	3.76	1501.6	0.3073
28	4.73	1892.7	0.0316
29	4.89	1955.9	0.1151
30	11.27	4505.3	0.0461
31	11.33	4530.0	0.0327

No.	Annotation	(ppm)
1	11,11,11	[1.05 .. 1.14]
2	18eq,20eq,22eq,18ax,20ax,22ax,3ax	[1.60 .. 1.68]
3	16eq,23eq,24eq,16ax,23ax,24ax,5ax	[1.77 .. 1.85]
4	17,19,21,3eq,5eq	[1.89 .. 2.25]
5	2eq,6eq,2ax,6ax	[2.99 .. 3.28]
6	7,7,10,10	[3.38 .. 3.46]
7	8,8	3.76
8	4	[4.69 .. 4.93]
9	25	[11.20 .. 11.42]

No.	(ppm)	Value	Absolute Value
1	[11.18 .. 11.44]	0.954	7.65989e+0
2	[4.66 .. 4.95]	1.008	8.09403e+0
3	[3.67 .. 3.82]	2.243	1.80130e+1
4	[1.01 .. 1.15]	3.194	2.56464e+1
5	[2.95 .. 3.22]	3.873	3.11005e+1
6	[3.30 .. 3.51]	4.266	3.42500e+1
7	[1.88 .. 2.27]	5.269	4.23075e+1
8	[1.54 .. 1.70]	6.572	5.27716e+1
9	[1.70 .. 1.88]	7.657	6.14818e+1

Fig. S21. ^{1}H NMR spectra of compound **4d** (in DMSO)

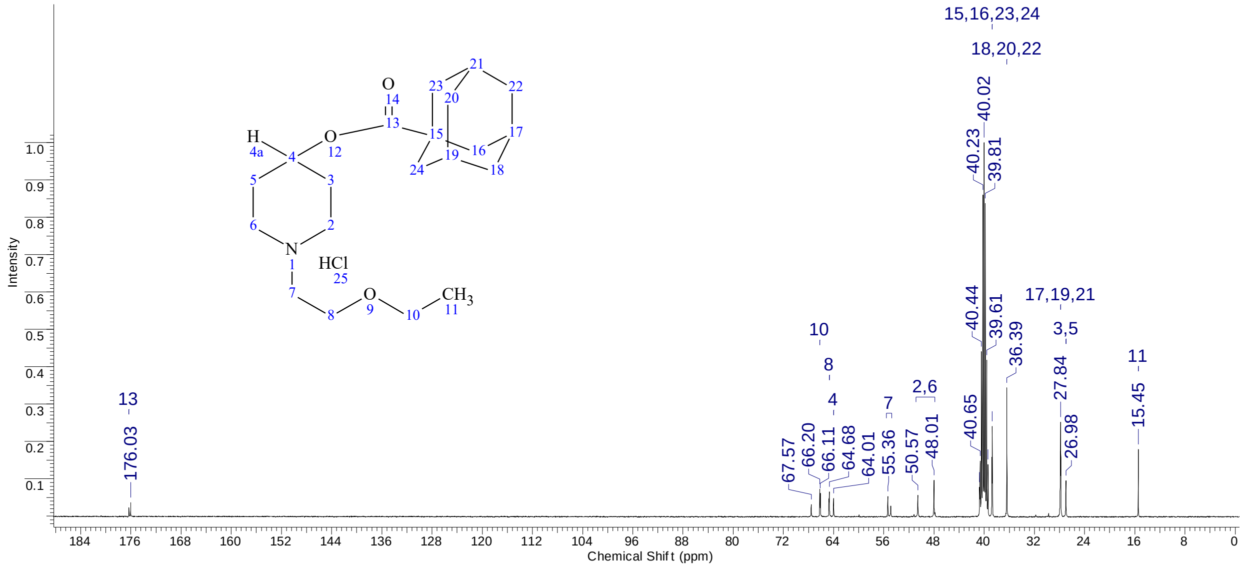

No.	(ppm)	(Hz)	Height
1	15.45	1552.8	0.1783
2	26.98	2712.6	0.0952
3	27.79	2793.1	0.1732
4	27.84	2798.9	0.2516
5	36.39	3658.6	0.3439
6	38.70	3890.5	0.2401
7	38.78	3898.2	0.1575
8	39.40	3960.5	0.1381
9	39.61	3981.6	0.4173
10	39.81	4001.7	0.8373
11	40.02	4022.8	1.0000
12	40.23	4043.9	0.8597

No.	(ppm)	(Hz)	Height
13	40.44	4065.0	0.4404
14	40.65	4086.1	0.1480
15	40.73	4094.7	0.0780
16	48.01	4826.0	0.0966
17	50.57	5083.8	0.0560
18	55.36	5565.0	0.0532
19	64.01	6434.3	0.0480
20	64.68	6502.4	0.0656
21	66.11	6646.1	0.0609
22	66.20	6654.8	0.0722
23	67.57	6792.8	0.0313
24	176.03	17695.3	0.0369

No.	Annotation	(ppm)
1	11	15.45
2	3,5	26.98
3	17,19,21	27.84
4	18,20,22	36.39
5	15,16,23,24	38.70
6	2,6	[47.86 .. 50.87]
7	7	[54.75 .. 55.62]
8	4	64.01
9	8	64.68
10	10	66.20
11	13	176.31

Fig. S22. ^{13}C NMR spectra of compound **4d** (in DMSO)

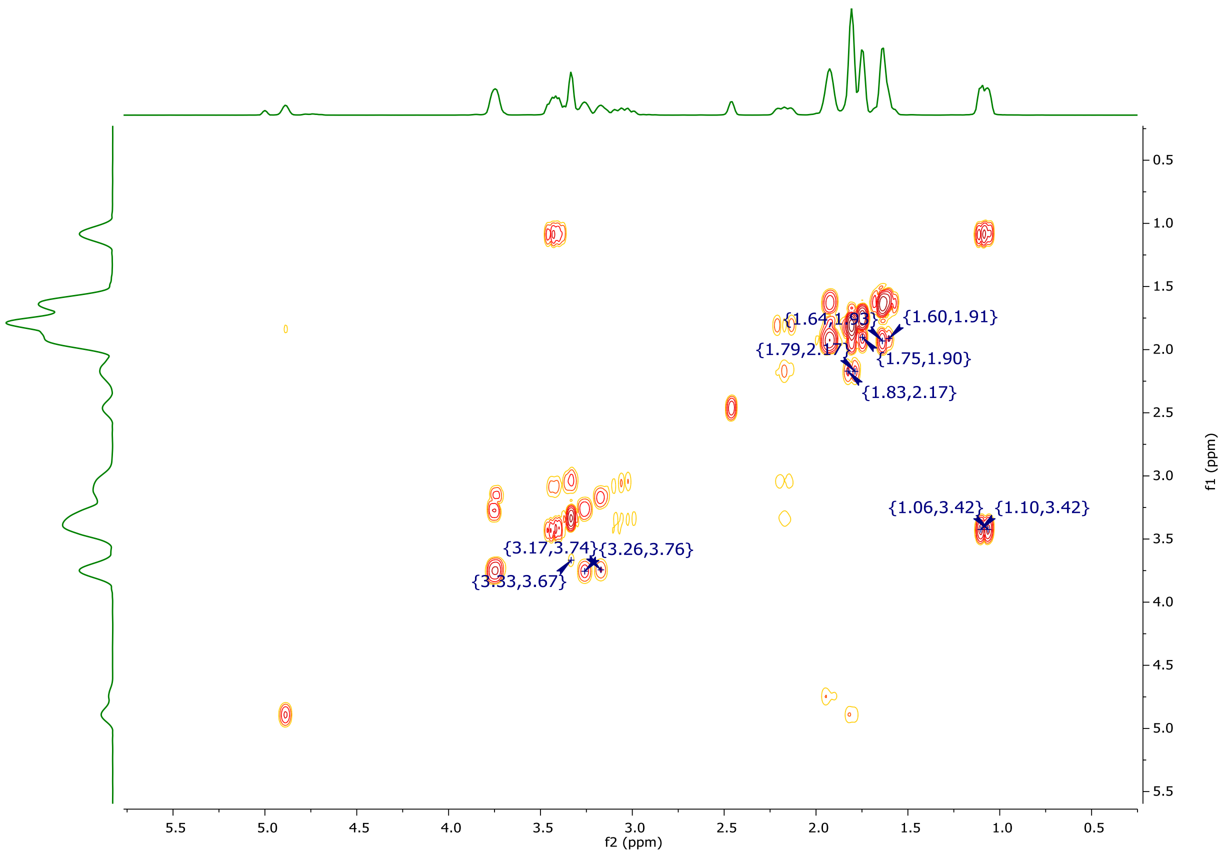

Fig. S23. COSY of compound **4d** (in DMSO)

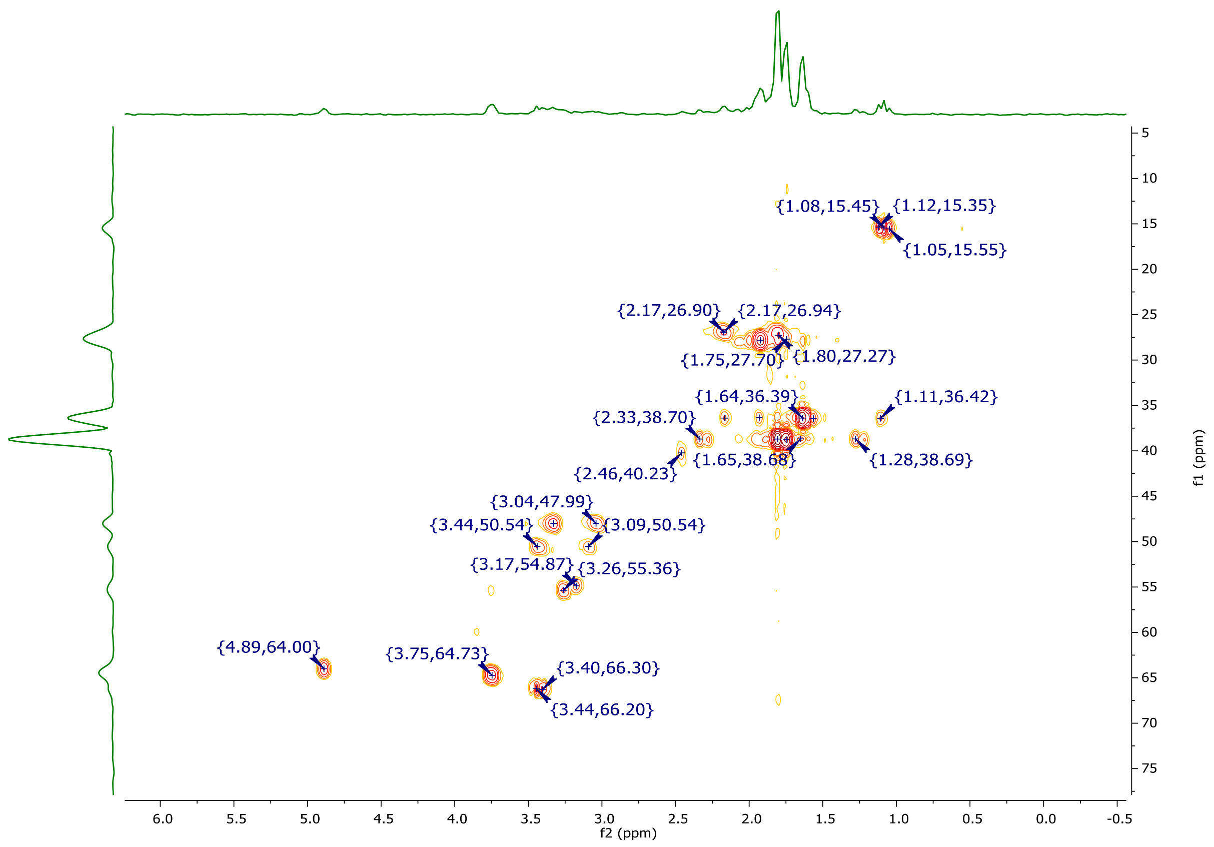

Fig. S24. HMQC of compound **4d** (in DMSO)

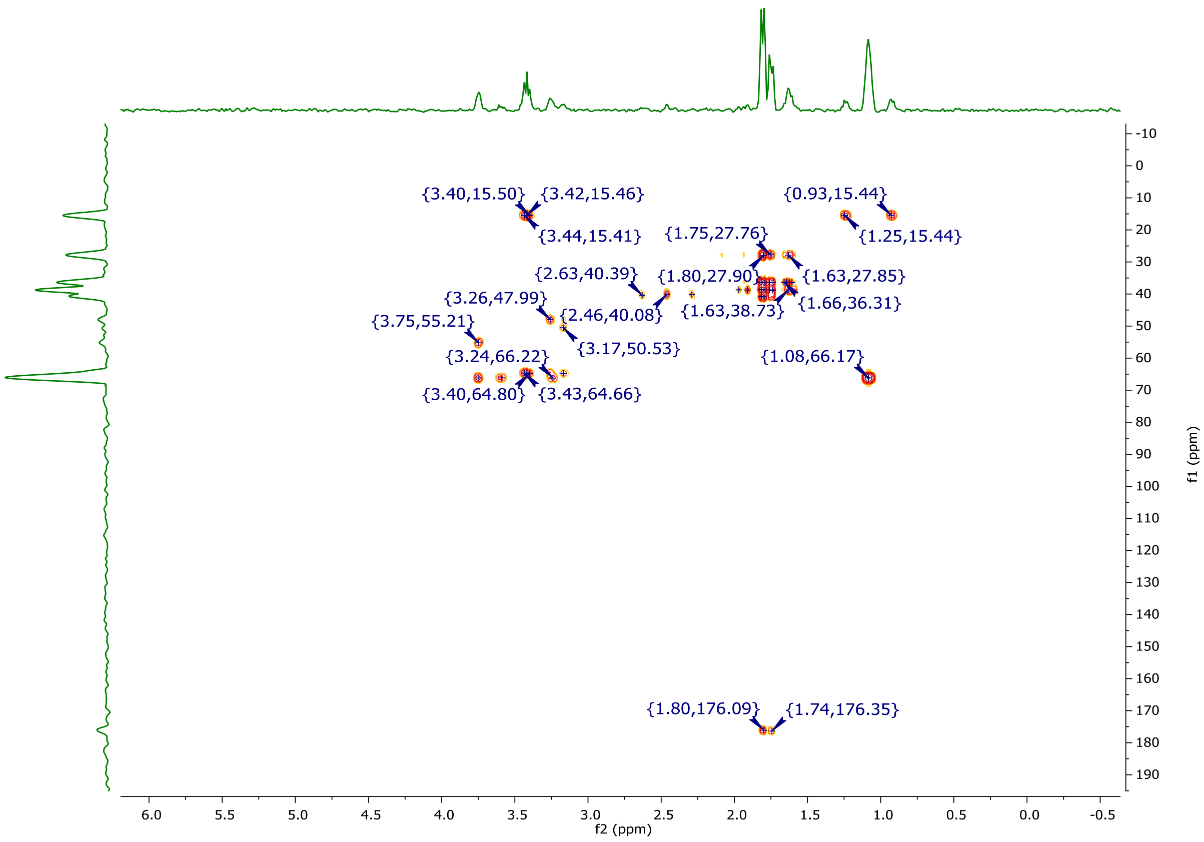

Fig. S25. HMBC of compound **4d** (in DMSO)

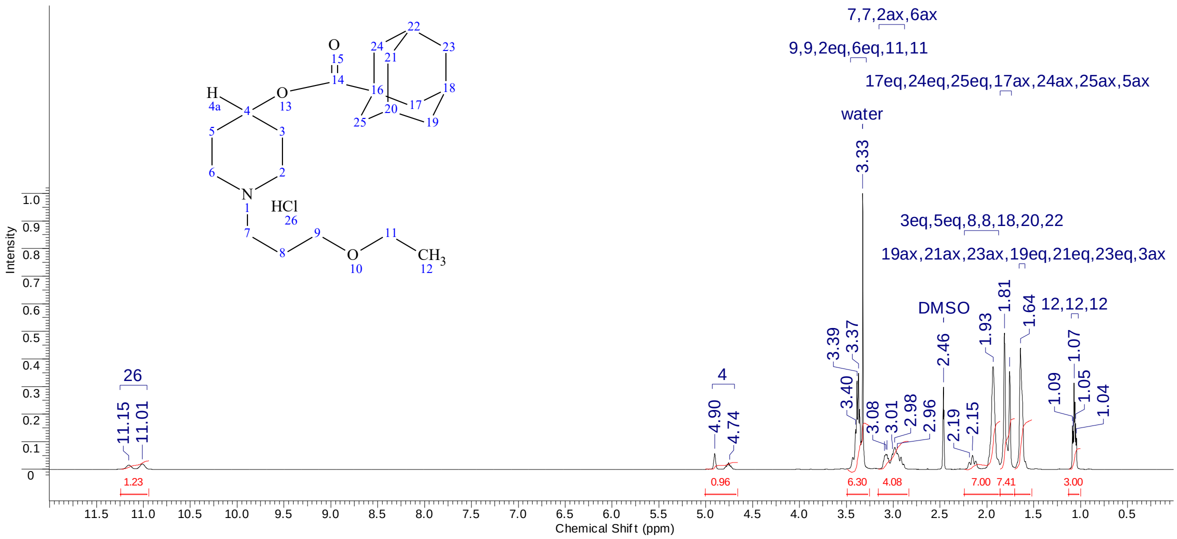

No.	(ppm)	(Hz)	Height	No.	(ppm)	(Hz)	Height
1	1.04	417.6	0.1134	15	2.98	1192.9	0.0800
2	1.05	420.8	0.1665	16	3.01	1203.0	0.0559
3	1.06	424.5	0.2437	17	3.07	1225.9	0.0534
4	1.07	427.7	0.3131	18	3.08	1230.5	0.0545
5	1.08	431.4	0.1384	19	3.09	1236.4	0.0501
6	1.09	435.0	0.1582	20	3.33	1329.4	1.0000
7	1.64	656.7	0.4395	21	3.36	1342.7	0.2180
8	1.76	702.9	0.3550	22	3.37	1347.3	0.3488
9	1.81	725.4	0.4940	23	3.39	1354.1	0.3204
10	1.93	773.4	0.3716	24	3.40	1359.6	0.1448
11	2.15	860.9	0.0506	25	4.74	1896.3	0.0180
12	2.19	874.7	0.0259	26	4.90	1960.9	0.0574
13	2.46	985.0	0.2982	27	11.01	4401.8	0.0196
14	2.96	1181.9	0.0584	28	11.15	4459.5	0.0152

No.	Annotation	(ppm)
1	12,12,12	[1.03 .. 1.10]
2	19ax,21ax,23ax,19eq,21eq,23eq,3ax	[1.59 .. 1.66]
3	17eq,24eq,25eq,17ax,24ax,25ax,5ax	[1.74 .. 1.86]
4	3eq,5eq,8,8,18,20,22	[1.88 .. 2.24]
5	DMSO	2.46
6	7,7,2ax,6ax	[2.88 .. 3.15]
7	9,9,2eq,6eq,11,11	[3.29 .. 3.45]
8	water	3.33
9	4	[4.69 .. 4.93]
10	26	[10.96 .. 11.25]

No.	(ppm)	Value	Absolute Value
1	[4.66 .. 5.00]	0.965	7.84460e+0
2	[10.94 .. 11.24]	1.232	1.00194e+1
3	[1.00 .. 1.13]	3.000	2.43887e+1
4	[2.83 .. 3.16]	4.078	3.31510e+1
5	[3.25 .. 3.49]	6.304	5.12521e+1
6	[1.86 .. 2.24]	6.999	5.69012e+1
7	[1.52 .. 1.70]	7.232	5.87967e+1
8	[1.70 .. 1.86]	7.406	6.02102e+1

Fig. S26. ^{1}H NMR spectra of compound **4e** (in DMSO)

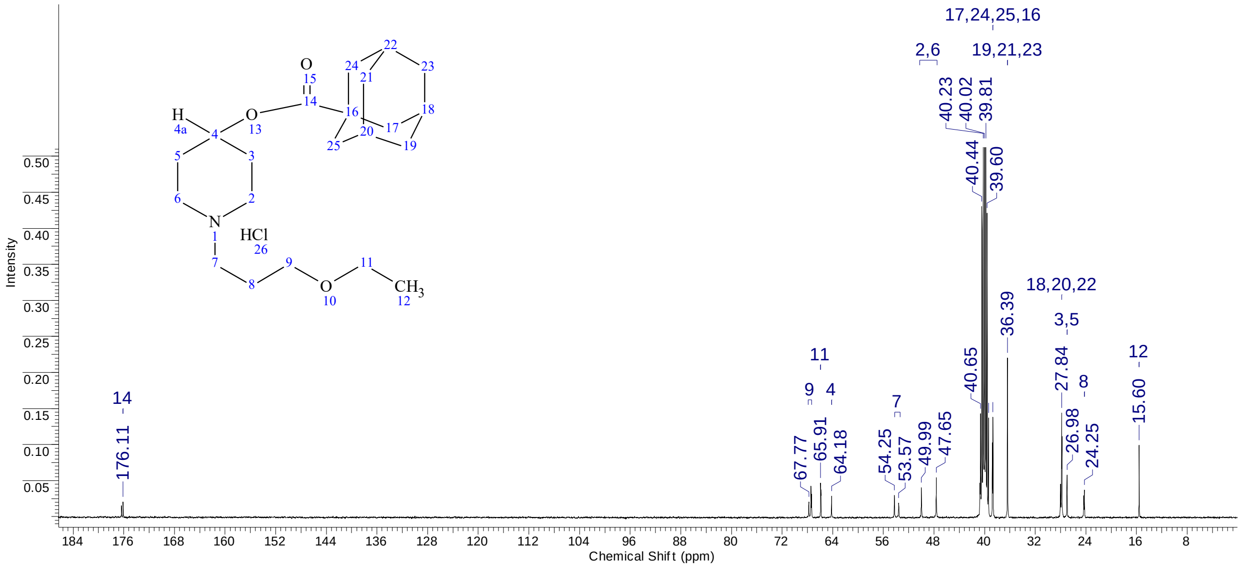

No.	(ppm)	(Hz)	Height
1	15.60	1568.2	0.0992
2	24.25	2437.5	0.0372
3	26.98	2712.6	0.0579
4	27.78	2792.1	0.1112
5	27.84	2798.9	0.1441
6	36.39	3658.6	0.2203
7	38.67	3887.7	0.1380
8	38.78	3898.2	0.1030
9	39.39	3959.6	0.1368
10	39.60	3980.6	0.4214
11	39.81	4001.7	0.8473
12	40.02	4022.8	1.0000

No.	(ppm)	(Hz)	Height
13	40.23	4043.9	0.8515
14	40.44	4065.0	0.4304
15	40.65	4086.1	0.1429
16	47.65	4789.6	0.0541
17	49.99	5025.4	0.0400
18	53.57	5384.8	0.0191
19	54.25	5453.8	0.0297
20	64.18	6451.6	0.0287
21	65.91	6626.0	0.0470
22	67.77	6812.9	0.0203
23	176.11	17703.0	0.0206

No.	Annotation	(ppm)
1	12	15.60
2	8	24.25
3	3,5	26.98
4	18,20,22	27.84
5	19,21,23	36.39
6	17,24,25,16	38.67
7	2,6	[47.54 .. 50.25]
8	7	[53.38 .. 54.22]
9	4	64.18
10	11	65.91
11	9	[67.31 .. 67.88]
12	14	176.11

Fig. S27. ^{13}C NMR spectra of compound **4e** (in DMSO)

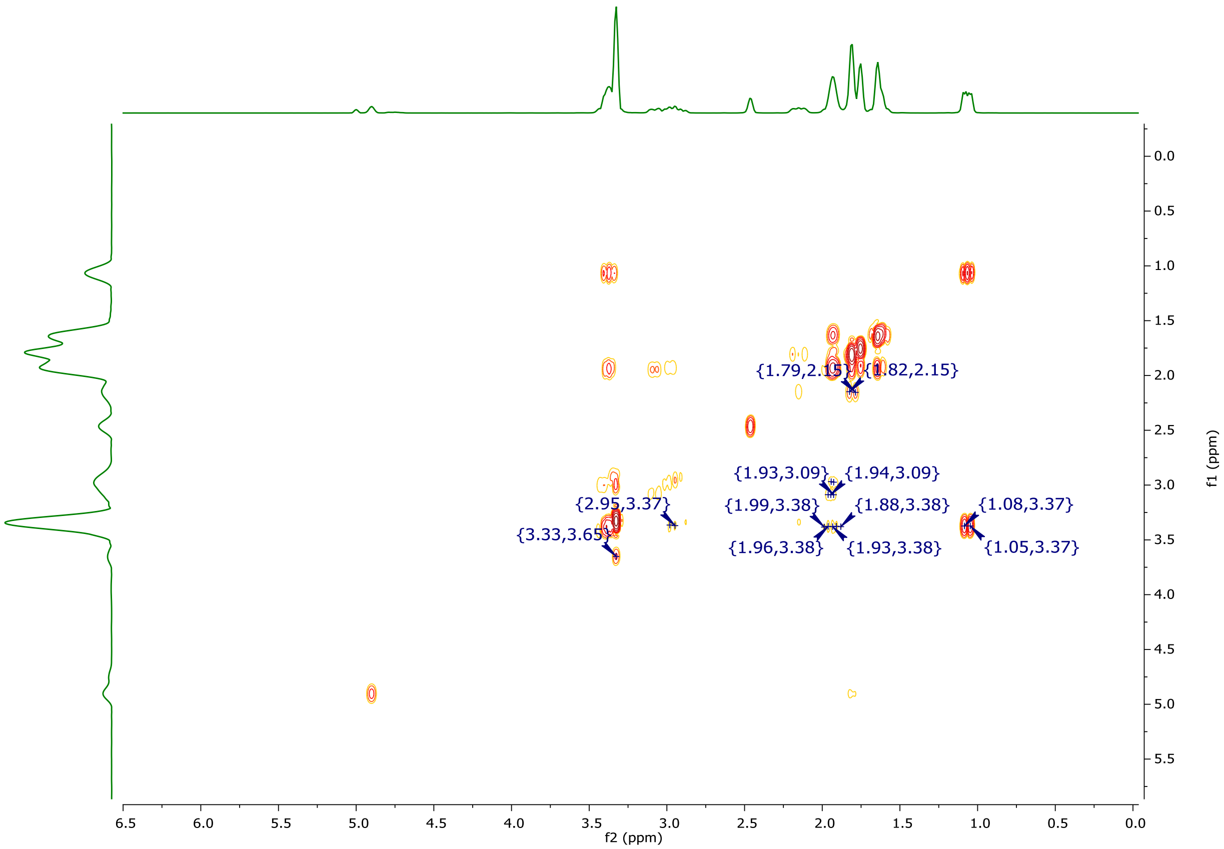

Fig. S28. COSY of compound **4e** (in DMSO)

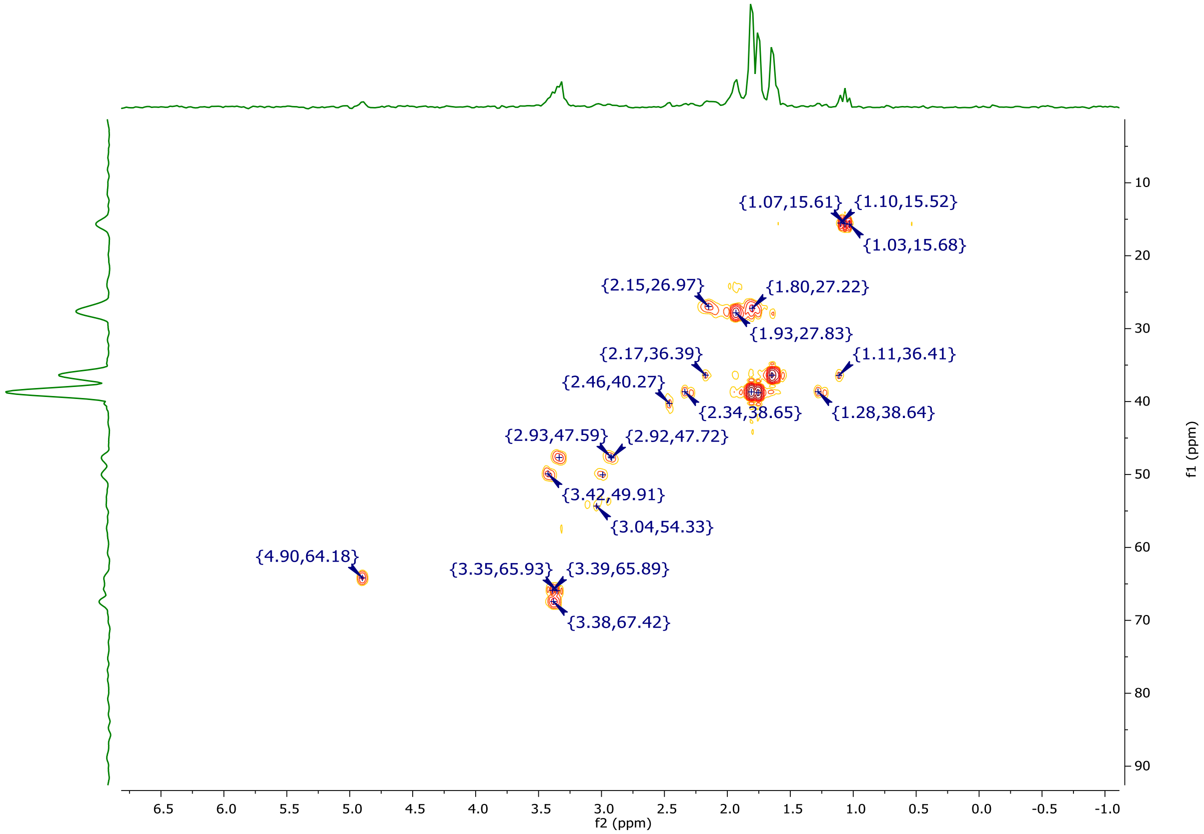

Fig. S29. HMQC of compound **4e** (in DMSO)

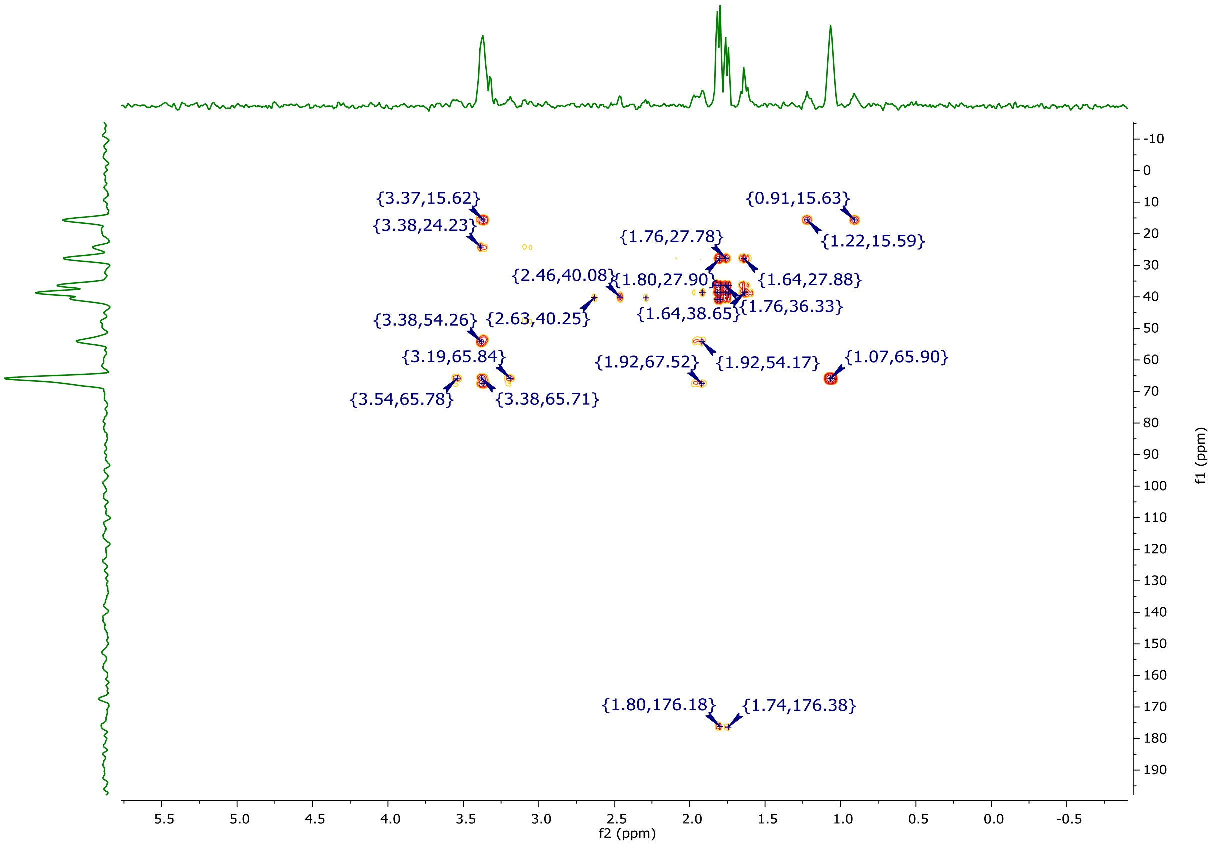

Fig. S30. HMBC of compound **4e** (in DMSO)

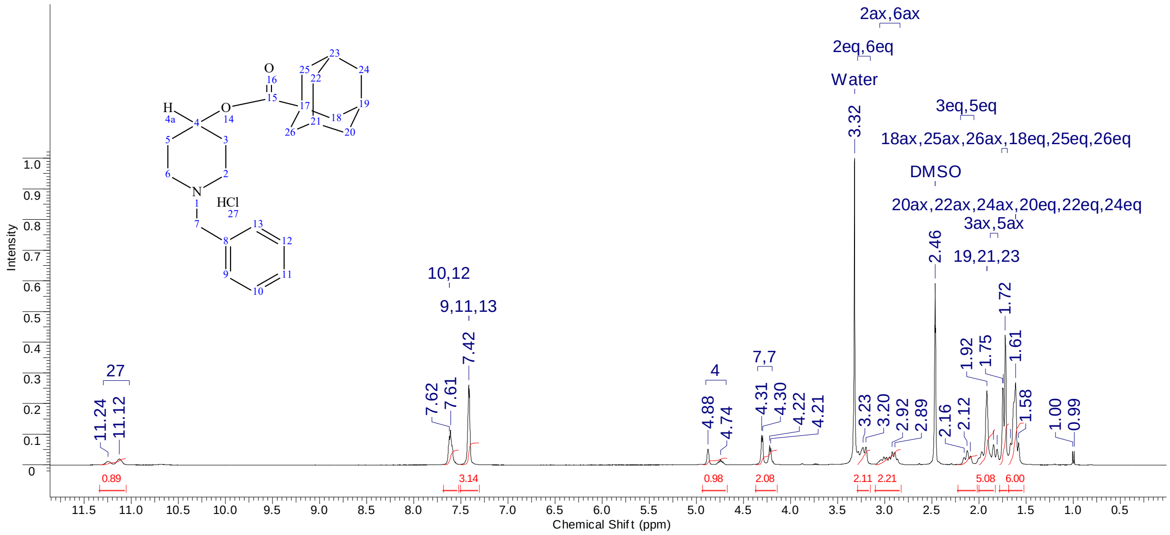

No.	(ppm)	(Hz)	Height
1	0.99	395.2	0.0446
2	1.00	401.1	0.0439
3	1.58	631.5	0.0725
4	1.61	643.8	0.2675
5	1.66	664.9	0.0716
6	1.72	687.8	0.4230
7	1.74	696.5	0.2505
8	1.75	698.3	0.2508
9	1.81	722.2	0.0512
10	1.84	737.3	0.0671
11	1.92	765.7	0.2418
12	2.09	835.3	0.0293
13	2.12	849.0	0.0466
14	2.16	862.3	0.0244
15	2.46	985.0	0.5918
16	2.89	1155.4	0.0417
17	2.92	1167.7	0.0428
18	3.20	1279.0	0.0582
19	3.23	1291.8	0.0569
20	3.32	1327.1	1.0000
21	4.21	1682.9	0.0550
22	4.22	1688.0	0.0630
23	4.30	1717.3	0.0954
24	4.31	1721.9	0.0967
25	4.74	1895.4	0.0173
26	4.88	1949.0	0.0514
27	7.41	2962.0	0.2482
28	7.42	2964.7	0.2603
29	7.61	3041.7	0.1146
30	7.62	3047.2	0.0934
31	11.12	4447.6	0.0195
32	11.24	4495.2	0.0113

No.	Annotation	(ppm)
1	20ax,22ax,24ax,20eq,22eq,24eq	1.61
2	18ax,25ax,26ax,18eq,25eq,26eq	[1.70 .. 1.75]
3	3ax,5ax	[1.80 .. 1.88]
4	19,21,23	1.92
5	3eq,5eq	[2.05 .. 2.20]
6	DMSO	2.46
7	2ax,6ax	[2.84 .. 3.05]
8	2eq,6eq	[3.15 .. 3.29]
9	Water	3.32
10	7,7	[4.20 .. 4.35]
11	4	[4.68 .. 4.90]
12	9,11,13	7.42
13	10,12	7.62
14	27	[11.02 .. 11.30]

No.	(ppm)	Value	Absolute Value
1	[11.05 .. 11.33]	0.886	5.97835e+0
2	[4.67 .. 4.93]	0.979	6.60377e+0
3	[2.02 .. 2.22]	1.360	9.17189e+0
4	[4.14 .. 4.37]	2.079	1.40248e+1
5	[7.52 .. 7.68]	2.100	1.41668e+1
6	[3.15 .. 3.29]	2.115	1.42650e+1
7	[2.83 .. 3.10]	2.214	1.49358e+1
8	[7.30 .. 7.50]	3.142	2.11932e+1
9	[1.83 .. 2.00]	5.082	3.42810e+1
10	[1.68 .. 1.78]	5.865	3.95604e+1
11	[1.52 .. 1.68]	6.000	4.04718e+1

Fig. S31. ^{1}H NMR spectra of compound **4f** (in DMSO)

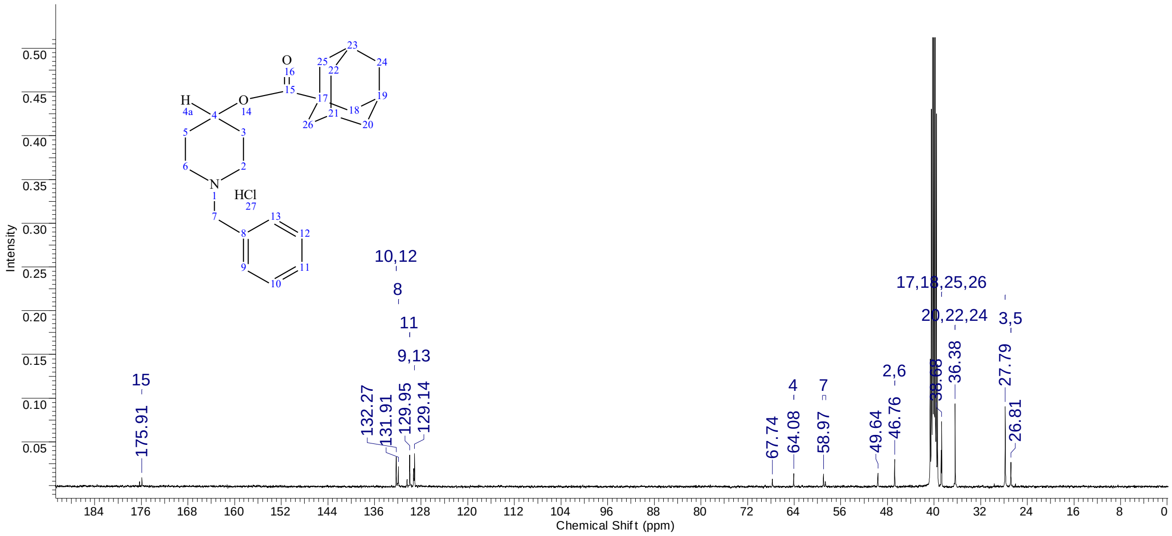

No.	(ppm)	(Hz)	Height
1	26.81	2695.3	0.0266
2	27.79	2794.1	0.0904
3	36.38	3656.7	0.0937
4	38.68	3888.6	0.0732
5	46.76	4700.5	0.0299
6	49.64	4989.9	0.0140
7	58.97	5928.2	0.0132
8	64.08	6442.0	0.0139
9	67.74	6810.0	0.0077
10	129.14	12981.6	0.0369
11	129.95	13063.1	0.0352
12	131.91	13260.5	0.0219
13	132.27	13296.9	0.0330
14	175.91	17683.8	0.0093

No.	Annotation	(ppm)
1	3,5	26.81
2	19,21,23	27.79
3	20,22,24	36.38
4	17,18,25,26	38.68
5	2,6	46.76
6	7	[58.57 .. 59.15]
7	4	64.08
8	9,13	129.14
9	11	129.95
10	8	131.91
11	10,12	132.27
12	15	175.91

Fig. S32. ^{13}C NMR spectra of compound **4f** (in DMSO)

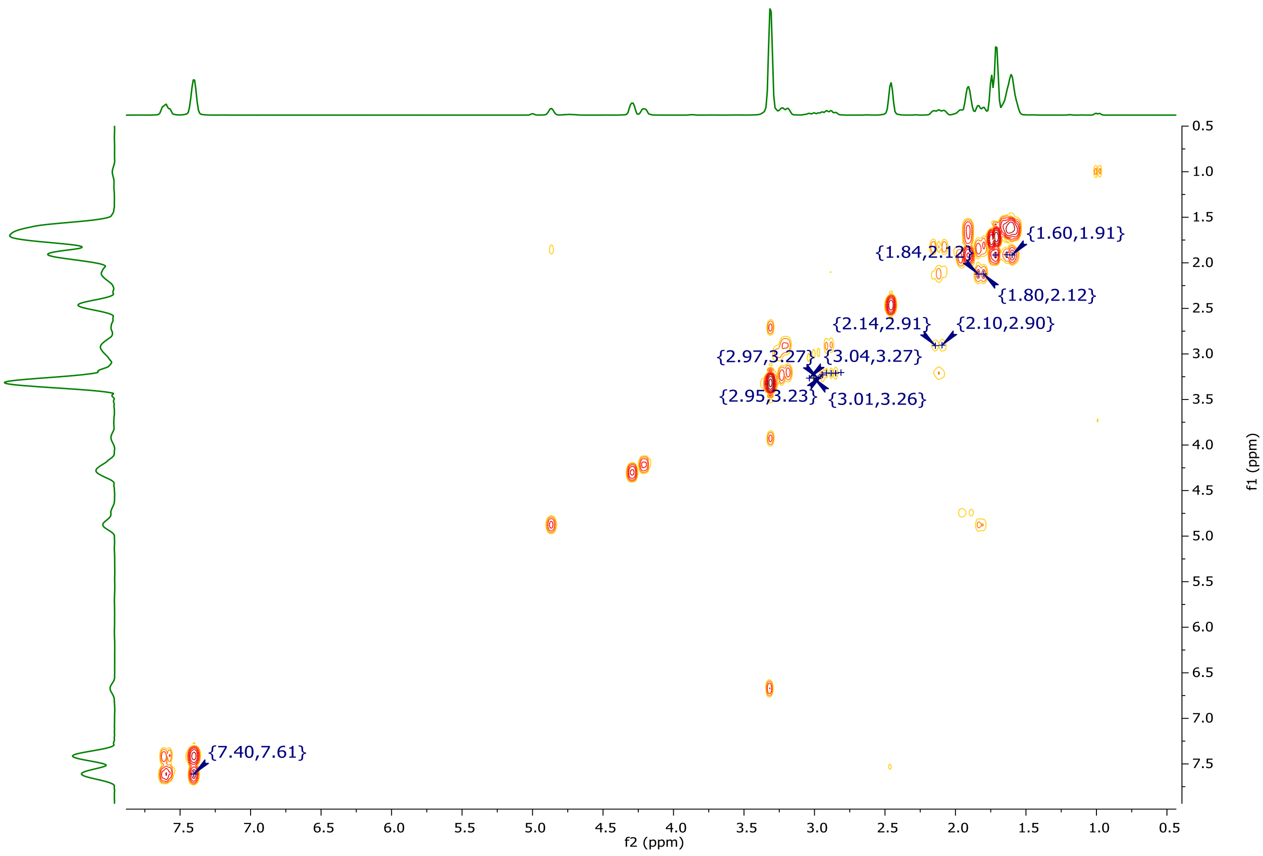

Fig. S33. COSY of compound **4f** (in DMSO)

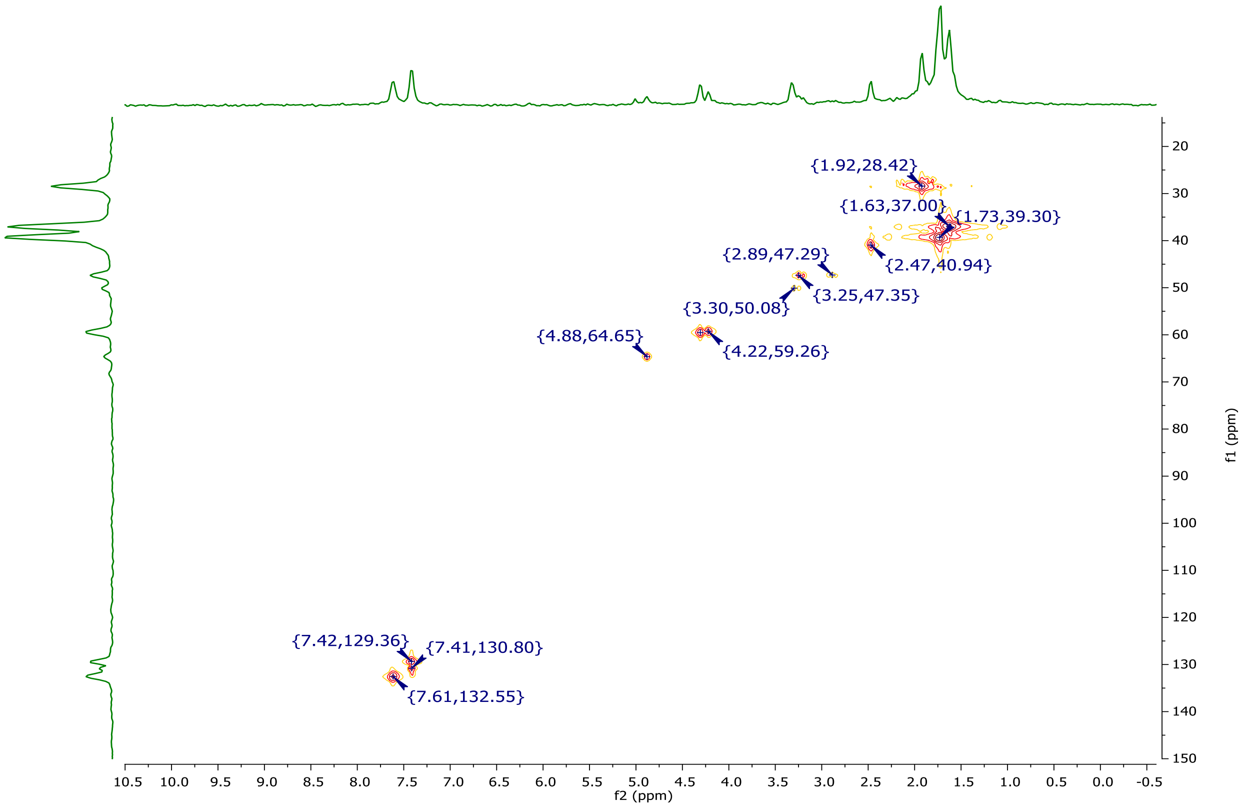

Fig. S34. HMQC of compound **4f** (in DMSO)

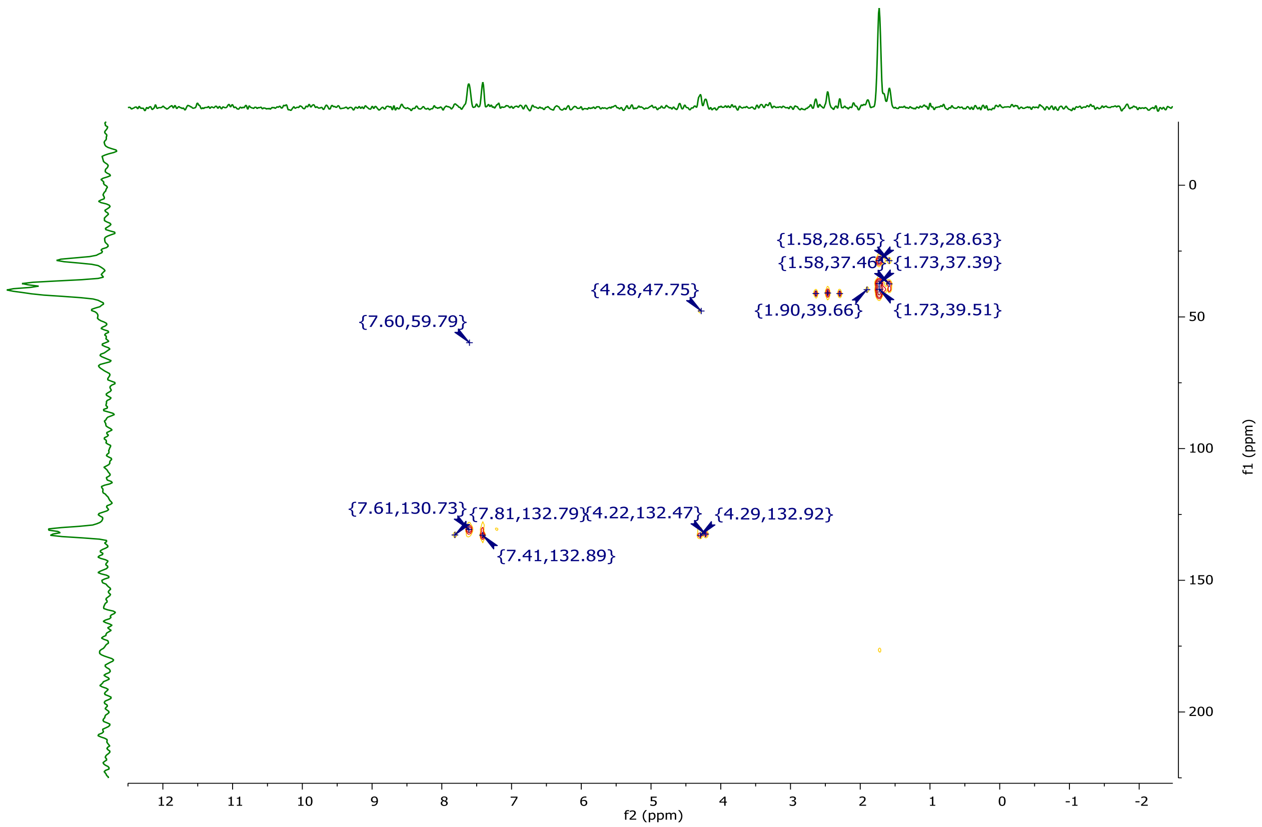

Fig. S35. HMBC of compound **4f** (in DMSO)
